# Asperversins A and B, Two Novel Meroterpenoids with an Unusual 5/6/6/6 Ring from the Marine-Derived Fungus *Aspergillus versicolor*

**DOI:** 10.3390/md16060177

**Published:** 2018-05-23

**Authors:** Huaqiang Li, Weiguang Sun, Mengyi Deng, Changxing Qi, Chunmei Chen, Hucheng Zhu, Zengwei Luo, Jianping Wang, Yongbo Xue, Yonghui Zhang

**Affiliations:** Hubei Key Laboratory of Natural Medicinal Chemistry and Resource Evaluation, Tongji Medical College, Huazhong University of Science and Technology, Wuhan 430030, China; lihuaqiang2009@126.com (H.L.); weiguang_s@hust.edu.cn (W.S.); DengMengyi222@163.com (M.D.); kingqcx@sina.cn (C.Q.); chenchunmei@hust.edu.cn (C.C.); zhuhucheng@hust.edu.cn (H.Z.); luozengwei@hust.edu.cn (Z.L.)

**Keywords:** *Aspergillus versicolor*, meroterpenoids, asperversins, acetylcholinesterase enzyme

## Abstract

Asperversins A (**1**) and B (**2**), two novel meroterpenoids featuring an uncommon 5/6/6/6 ring system, along with five new analogues (**3**–**7**) and a known compound asperdemin (**8**), were obtained from the marine-derived fungus *Aspergillus versicolor*. Their structures and absolute configurations were confirmed by extensive spectroscopic analyses, single-crystal X-ray diffraction studies, and electronic circular dichroism (ECD) calculation. All new compounds were tested for their acetylcholinesterase enzyme (AChE) inhibitory activities and cytotoxic activities, of which compound **7** displayed moderate inhibitory activity against the AChE with an IC_50_ value of 13.6 μM.

## 1. Introduction 

Meroterpenoids are hybrid secondary metabolites that partially derive from the terpenoid pathways [1]. Naturally occurring meroterpenoids have been isolated from a variety of sources including animals, plants, bacteria, and fungi, [2] and are exemplified by ubiquinone-10 (coenzyme Q10) [3], α-tocopherol (vitamin E) [4], vinblastine [5], merochlorin A [6,7], and teleocidin B-4 [8].

Biosynthetically, the complex structures of fungal meroterpenoids are mostly derived from simple precursors like a linear isoprenoid or the C-2 carbon unit acetyl-CoA, via a series of chemical transformations catalyzed by two enzyme families, terpene cyclases and polyketide synthases (PKSs) [9,10]. In addition to the enormous structural diversity, fungal meroterpenoids have attracted wide interest from the scientific community due to their broad spectrum of pharmacological activities [11,12,13,14].

During our investigation of bioactive metabolites from fungi [15,16,17,18,19], many novel meroterpenoids were isolated from various fungi. Asperterpene A, obtained from *Aspergillus terreus*, represents a potential lead compound and a versatile scaffold for the development of drugs for the treatment of Alzheimer’s disease [15]. Apiroaspertrione A, bearing a unique spiro[bicyclo[3.2.2]nonane-2,1′-cyclohexane] carbocyclic skeleton, shows an effective activity against methicillin-resistant *Staphylococcus aureus* (MRSA) [16]. The metabolites of the fungus *Aspergillus versicolor*, that were obtained from mud from the South China Sea, are reported here. This endeavor resulted in the isolation of seven undescribed meroterpenoids, called as asperversins A–G (**1**–**7**), and a structurally related known compound, asperdemin (**8**) [20]. Notably, asperversins A (**1**) and B (**2**) have a unique 5/6/6/6 ring system that features a tetrahydrofuran ring. Herein, the isolation, structure elucidation, plausible biosynthetic pathway, and the biological evaluations of compounds **1**–**7** are presented.

## 2. Results and Discussion

Asperversin A (**1**), a white amorphous powder, was established to have a molecular formula of C_24_H_32_O_8_ by HRESIMS ion at *m/z* 449.2173 ([M + H]^+^, calcd. for C_24_H_33_O_8_, 449.2175), indicating 9 degrees of unsaturation. The strong IR absorptions at 1741 and 1703 cm^−1^ suggested the presence of ester and unsaturated ester groups. The ^1^H NMR data (in methanol-*d*_4_, Table 1) displayed an olefinic proton at *δ*_H_ 5.91 (d, *J* = 1.0 Hz, H-15), a methoxy group at *δ*_H_ 3.70 (s, OMe-24) and six methyl groups at *δ*_H_ 2.20 (d, *J* = 1.0 Hz, H_3_-17), 2.07 (s, H_3_-23), 1.43 (H_3_-18), 1.31 (H_3_-19), 1.26 (H_3_-20), and 1.18 (H_3_-21). The ^13^C and DEPT spectra revealed the presence of three carbonyl carbons (*δ*_C_ 173.6, 171.6, and 167.1) and two enol systems (*δ*_C_ 165.1, 162.2, 102.2, and 98.5), three quaternary sp^3^ carbons (two oxygenated), four methines (two oxygenated), three methylene carbons, and seven methyl groups.

**Figure 1:**
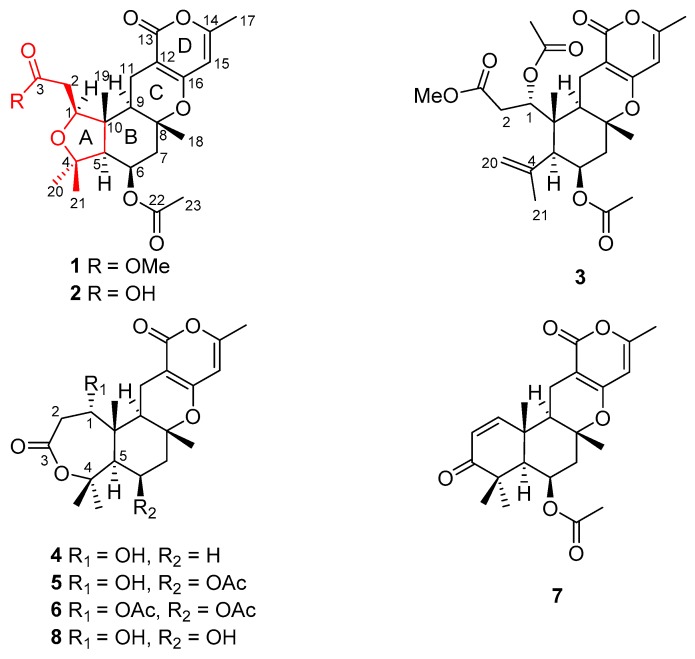
The structures of compounds **1**–**8**.

Comparison of the ^1^H and ^13^C NMR data (Table 1 and Table 2) of **1** with the known compound asperdemin (**8**) [20] revealed their close similarities, except for the presence of an additional methoxy group (*δ*_H_ 3.70, *δ*_C_ 52.3) and an acetoxyl unit. Further analyses of the ^1^H–^1^H COSY and HMBC spectra of **1** indicated that **1** shared the same B/C/D ring system as that of **8** (Figure 1). The key HMBC correlation from H-6 to C-22 (*δ*_C_ 171.6) indicated the acetoxyl group at C-6. Furthermore, the ^1^H–^1^H COSY cross-peaks of H-1/H_2_-2 and the HMBC connections from H-1 to C-3, from H_2_-2 to C-3, and from OMe to C-3 confirmed the existence of a methyl ester group in **1**, rather than a seven-membered lactone. Although there was no obvious HMBC correlation to connect C-1 and C-4, the absence of hydroxyl at C-1 confirmed by the 1D and 2D NMR data in DMSO-*d*_6_ and the chemical shifts of C-1 (*δ*_C_ 81.9 for **1**; 68.0 for **8**) and C-4 (*δ*_C_ 77.7 for **1**; 84.8 for **8**) indicated that C-1 and C-4 were connected with an ether bond to establish a tetrahydrofuran ring, which was consistent with the unsaturation degrees deduced by HRESIMS. Thus, the unusual tetrahydrofuran A ring replaced the seven-membered lactone ring, which distinguished compound **1** from other metabolites reported in this compound class.

**Figure 2:**
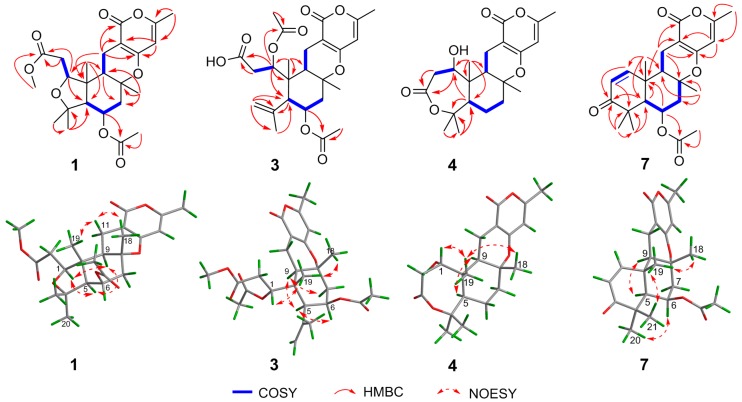
Key 2D correlations of compounds **1**, **3**, **4** and **7**.

**Figure 3:**
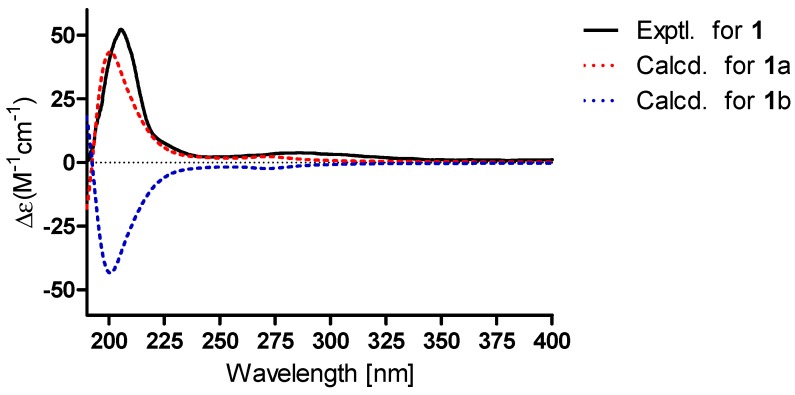
The experimental and calculated ECD of **1**.

The relative configuration of **1** was partially elucidated by the examination of its Nuclear Overhauser Effect SpectrocopY (NOESY) spectrum (Figure 2). The configuration of Me-19 was arbitrarily assigned as same as that of **8** in a *β*-orientation. Furthermore, the NOESY correlations of H_3_-18/H-11b and H-11b/H_3_-19 suggested their cofacial and *β*-orientation. In contrast, H-9 should be *α*-oriented. The NOESY correlations of H-1/H-9, H-9/H-5, and H-1/H-5 indicated their *α*-orientation. The spin-coupling constant (*J* = 2.3 Hz) of H-5 and H-6 suggested H-6 was equatorial. To confirmed the absolute configuration, the ECD calculation of **1** [1S,5S,6R,8R,9R,10R-1 (1a) and 1R,5R,6S,8S,9S,10S-1 (1b)] was conducted in MeOH using the time-dependent density functional theory (TD-DFT) method at the B3LYP/6-311+g (d, p) level. The experimental ECD of **1** matched well with the calculated ECD curve of **1a**, indicating the absolute configuration of **1** as 1*S*,5*S*,6*R*,8*R*,9*R*,10*R* (Figure 3).

**Figure 4:**
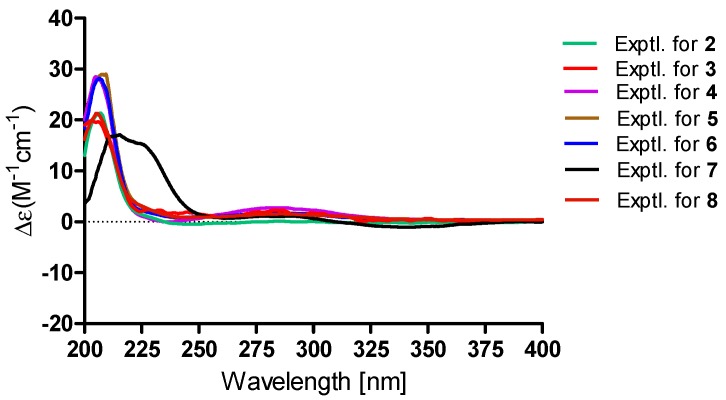
The experimental ECD of **2**–**8**.

The HRESIMS spectrum suggested a molecular formula of C_23_H_30_O_8_ for compound **2**, indicating a molecular weight of 14 mass units lower than **1**. The ^1^H and ^13^C NMR data of **2** (Table 1 and Table 2) closely resembled those of compound **1**, except for the difference of chemical shift of carbonyl (C-3: *δ*_C_ 176.7 for **2**; 173.6 for **1**), and the absence of the methoxy group. Compound **2** was speculated to be the carboxylic acid analogue of **1**. The structure of **2** was confirmed by extensive analyses of its ^1^H–^1^H COSY and HMBC spectra. Compound **2** was confirmed to have the same relative and absolute configurations as **1** by the NOESY spectra and their similar ECD curves (Figure 1, Figure 3 and Figure 4).

Asperversin C (**3**), a white amorphous powder, has a molecular formula of C_26_H_34_O_9_, which was determined on the basis of the HRESIMS ion peak at *m*/*z* 491.2269 ([M + H]^+^, calcd. for C_26_H_35_O_9_, 491.2281). The similar IR, UV, 1D, and 2D NMR spectra of **3** and **1** suggested both compounds shared the same B/C/D rings. A comparison of their 1D and 2D NMR data indicated the presence of a double bond (*δ*_H_ 5.05 and 5.08, *δ*_C_ 117.7, CH_2_-20; *δ*_C_ 144.4, C-4) and the absence of a methyl group in compound **3**. The location of the double bond between C-4 and C-20 was confirmed by the HMBC correlations from H_2_-20 to C-4, C-5, and C-21, and from H_3_-21 to C-4 and C-20. Moreover, the HMBC correlations from H-1 to C-24 and H_3_-25 to C-24 defined the location of an additional acetoxyl unit at C-1. Finally, the planar structure of **3** with the A ring opened was confirmed as shown (Figure 1). The NOE correlations from H-1/H_3_-19, H_3_-18/H_3_-19 suggested H-1, Me-18, and Me-19 were *β*-oriented. In contrary, H-5, H-6 and H-9 were *α*-oriented based on the correlations of H-5/H-6 and H-5/H-9. In consideration of the similar specific rotation and ECD spectra (Figure 4), the absolute configuration of **3** was assigned as 1*R*,5*S*,6*R*,8*R*,9*R*,10*R*.

Asperversin D (**4**) possesses a molecular formula of C_21_H_28_O_6_ with 16 units less than that of compound **8** on the basis of HRESIMS data. Comparison of the ^1^H and ^13^C NMR data of **4** with those of **8** revealed the presence of an additional methylene group and the absence of an oxygenated methine group in **4**. Inspection of the 2D NMR of **4** indicated this methylene group (*δ*_C_ 23.3; *δ*_H_ 1.67 and 1.92) should be assigned to C-6 by the ^1^H–^1^H COSY correlations of H-5/H_2_-6/H-7. The gross structure of **4** and its relative configuration were then confirmed by comprehensive analyses of the ^1^H–^1^H COSY, HMBC, and NOESY spectra. Finally, the absolute configuration of **4** was ascertained as 1*R*,5*S*,8*R*,9*R*,10*R* by a similar ECD spectrum to that of **1** (Figure 4).

**Figure 5:**
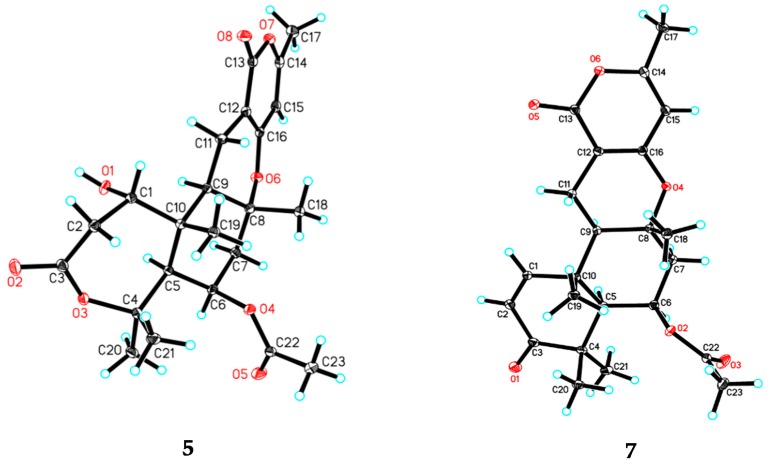
ORTEP drawings of compounds **5** and **7**.

Asperversin E (**5**), colorless crystals, exhibits a molecular formula of C_23_H_30_O_8_ with nine degrees of unsaturation from a HRESIMS ion peak at *m/z* 457.1830 ([M + Na]^+^, calcd. for 457.1838). Close similarities were observed in the 13C NMR data (Table 2) between compounds **5** and **4**. Inspection of the 1D NMR spectra, HSQC, and HMBC spectra revealed that **5** contained an additional acetoxyl group (*δ*_H_ 2.06, *δ*_C_ 21.4, and 170.2). Detailed HMBC correlations from H_3_-23 (*δ*_H_ 2.06) to C-22 (*δ*_C_ 170.2) and from H-6 (*δ*_H_ 5.52) to C-22 confirmed the location. The X-ray crystallographic diffraction (Figure 5) revealed the configuration of **5** was 1*R*,5*S*,6*R*,8*R*,9*R*,10*R*.

A protonated molecular ion peak at *m/z* 477.2098 ([M + H]^+^, calcd. 477.2125) was obtained from the HRESIMS experiment and indicated asperversin F (**6**) has the molecular formula C_25_H_32_O_9_. The ^1^H and ^13^C NMR spectra of **6** showed there were two more acetoxyl groups (*δ*_H_ 2.14, *δ*_C_ 21.3, and 171.2; *δ*_H_ 1.99, *δ*_C_ 20.6, and 171.1) than **4**. The locations of two acetoxyls were assigned to C-1 and C-6 by the HMBC correlations from H-1 (*δ*_H_ 4.96) to C-24 and from H-6 (*δ*_H_ 5.64) to C-22, respectively. The relative configuration and absolute configuration of **6** were determined by analyzing the NOESY spectrum and by comparing its ECD spectrum with that of **5** (Figure 4).

Asperversin G (**7**), colorless cubic crystals, has a molecular formula of C_23_H_28_O_6_ based on the HRESIMS spectrum. The ^1^H and ^13^C NMR data (Table 1 and Table 2) of **7** resembled those of **5**, with the main difference being the presence of an *α*,*β-*unsaturated ketone group (*δ*_H_ 7.08, d, *J* = 10.2 Hz and 5.87, d, *J* = 10.2 Hz; *δ*_C_ 203.2, 155.1, and 125.1). The *α*,*β-*unsaturated ketone further constructed an 2-cyclohexen-1-one (ring A), as ascertained by the HMBC correlations from H_3_-19 to C-1, C-5, C-9, and C-10, from H-1 to C-3, from H-2 to C-3 and C-4, and from H_3_-20 and H_3_-21 to C-3, C-4, and C-5. The remainder of **7** was identical to **5** as elucidated by 2D NMR spectra. The absolute configuration of **7** was established unambiguously as 5*S*,6*R*,8*R*,9*R*,10*R* by a single-crystal X-ray diffraction (Figure 5).

**Scheme 1:**
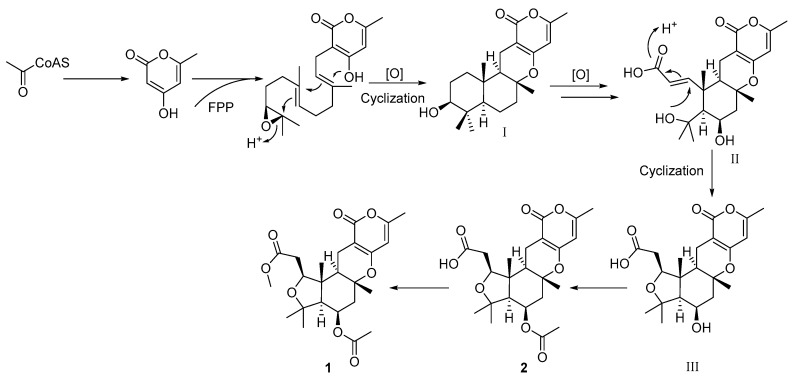
Postulated biosynthetic pathway of compounds **1** and **2**.

Asperversins A (**1**) and B (**2**) were found to be the first examples of pyrone meroterpenoids featuring an exclusive 5/6/6/6 ring system compared to all known meroterpenoids [2]. Therefore, a plausible biosynthetic pathway is proposed to illustrate the generation of **1** and **2** (Scheme 1). Asperversins A (**1**) and B (**2**) are probably biosynthesized via a polyketide and mevalonate hybrid biogenetic pathway [2,21,22]. After a series of oxidations, the A ring of **I** was opened, and then, followed by the key electrophilic addition of OH-4 with the *α*,*β-*unsaturated carboxylic acid bond, the intermediate **III**, featuring a tetrahydrofuran ring, was derived from **II**. Compounds **2** and **1** were produced subsequently by an additional acetylation and methylation.

All compounds (**1**–**8**) were inactive for cytotoxicity against A549, MCF-7, HepG2, and HL-60 cells, and did not have any antimicrobial effects against methicillin-resistant *Staphylococcus aureus* (MRSA), methicillin-sensitive *Staphylococcus aureus* (MSSA), *Escherichia coli*, or *Pseudomonas aeruginosa*. The acetylcholinesterase (AChE) inhibition effects of these compounds (**1**–**8**) were assessed by Ellman’s spectrophotometric method [23] using human recombinant AChE with galanthamine as control compound (Table 3). Compound **7** exhibited the strongest inhibition to AChE with an IC_50_ value of 13.6 μM, and compounds **1**–**6** and **8** showed no activities up to a concentration of 40 μM.

**Figure 6:**
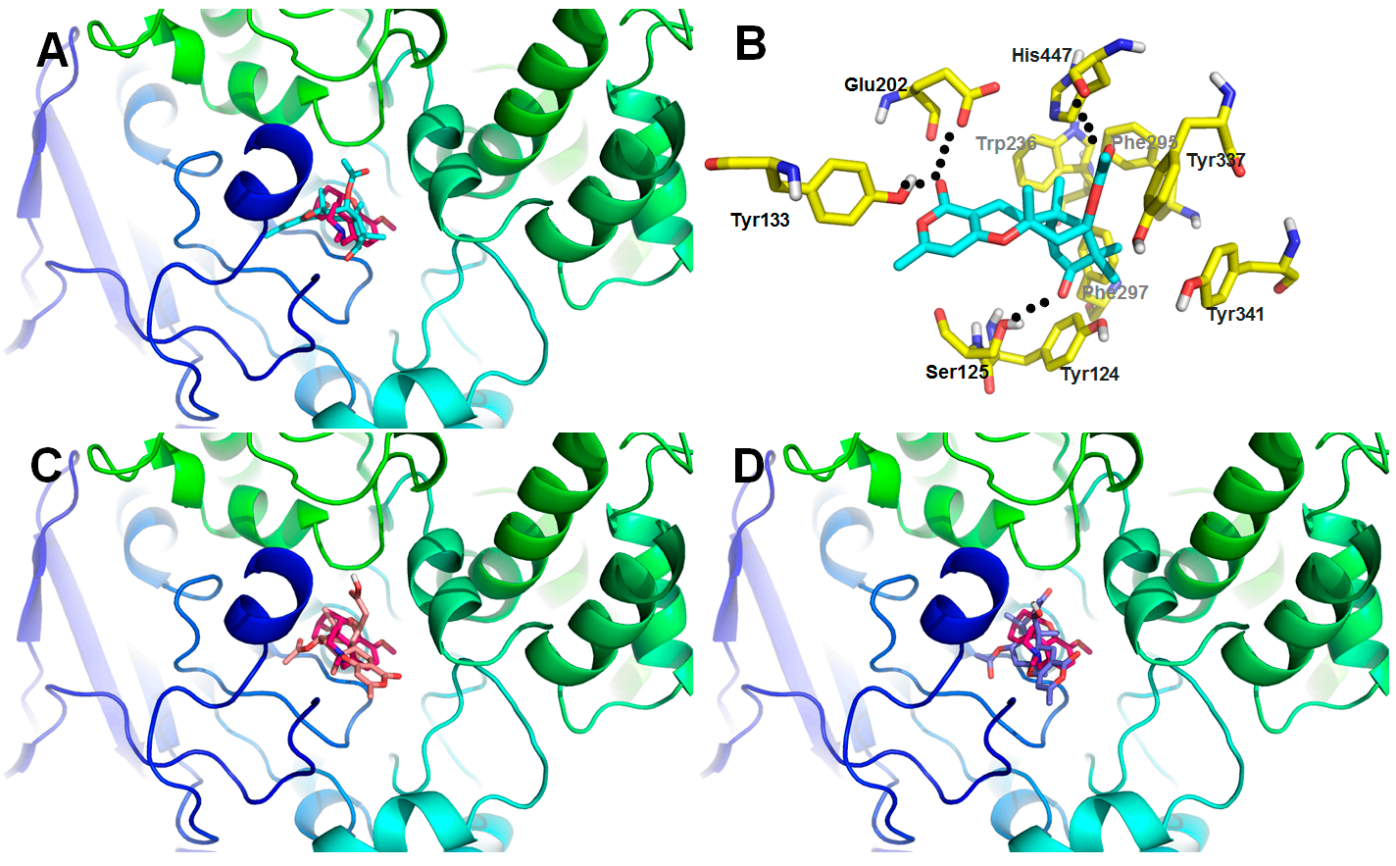
Low-energy binding conformations of compounds bound to AChE generated by virtual ligand docking. (**A**) Compound **7** was observed to occupy the active site with significant scores and adopted a conformation similar to that of galanthamine. (**B**) Compound **7** had the ability to form key hydrogen bonding interaction with residues Ser125, Tyr133, Glu202, and His447. (**C**,**D**) Compounds **2** (**C**) and **5** (**D**) were observed to occupy the active site with pretty low scores comparing with that of galanthamine.

The molecular docking studies were conducted in order to get an insight into the binding pattern and extent of binding of compound **7** with the target enzyme (Figure 6). This was mainly attributed to the basic skeleton of compound **7** which provided better binding prospects with the formation of hydrogen bonding interactions to the amino acid residues Glu 291 and Tyr 341 of the protein. So we speculated the *α*,*β-*unsaturated ketone group was the key pharmacophore for the acetylcholinesterase (AChE) inhibition effect.

## 3. Experimental Section

### 3.1. General Experimental Procedures

The NMR spectra were recorded on Bruker AM-400 and 600 spectrometers (Bruker, Karlsruhe, Germany). The ^1^H and ^13^C NMR chemical shifts were referenced to the solvent or solvent impurity peaks for methanol-*d*_4_ (*δ*_H_ 3.31 and *δ*_C_ 49.0), and acetone-*d*_6_ (*δ*_H_ 2.05 and *δ*_C_ 206.3 and 29.8). The FT-IR spectra were determined with a Bruker Vertex 70 instrument (Bruker, Karlsruhe, Germany). The UV and ECD spectra were measured using a Perkin Elmer Lambda 35 UV spectrophotometer (PerkinElmer, Inc., Fremont, CA, USA) and a JASCO-810 ECD spectrometer (JASCO Co., Ltd., Tokyo, Japan), respectively. Optical rotations were measured by an AUTOPOL IV-T Automatic polarimeter (Rudolph Research Analytical, Hackettstown, NJ, USA). HRESIMS data were obtained in the positive ion mode on a Thermo Fisher LTQ XL spectrometer (Thermo Fisher, Palo Alto, CA, USA). Crystal X-ray diffraction data were measured on a Rigaku XtaLAB PRO MM007HF (Rigaku, Tokyo, Japan). Semipreparative HPLC was carried out using an Agilent 1200 quaternary system with a UV detector using a reversed-phase C_18_ column (5 μm, 10 × 250 mm, Welch Ultimate XB-C18). Column chromatography (CC) was performed using silica gel (100–200 and 200–300 mesh; Qingdao Marine Chemical Inc., Qingdao, China), ODS (50 μm, YMC, Kyoto, Japan), and Sephadex LH-20 (GE Healthcare Bio-Sciences AB, Uppsala, Sweden). Thin-layer chromatography (TLC) was performed on silica gel 60 F254 (Yantai Chemical Industry Research Institute, Yantai, China).

### 3.2. Fungi Material

The fungus *Aspergillus versicolor* was isolated from mud of the South China Sea. The sequence data for this strain have been submitted to DDBJ/EMBL/GenBank under accession no. 2081031. A voucher sample has been deposited in the culture collection of Tongji Medical College, Huazhong University of Science and Technology, P. R. China.

### 3.3. Fermentation and Extraction

The *Aspergillus versicolor* strain was cultured on potato dextrose agar (PDA) at 28 °C for 7 days to prepare the seed culture and inoculated into 100 sterilized Erlenmeyer flasks (1 L), which contained 200.0 g of rice and 160.0 mL of distilled water, then incubated at 28 °C for 28 days. Following incubation, the rice was soaked with 95% EtOH many times until the solvent was near-colorless at room temperature. Finally, solvent removal afforded a brown extract that was suspended in water (2 L) and extracted with EtOAc (1:1, *v*/*v*) three times. After removal of EtOAc, a dark brown gum (170 g) was obtained.

### 3.4. Isolation

The extracts (170.0 g) were subjected to a silica gel chromatography column (CC) eluting with PE/ EtOAc (20:1–0:1) progressively to obtain six fractions (Fr. 1–Fr. 6). Fr.3 was further separated with silica gel CC to yield four subfractions (Fr. 3.1–Fr. 3.4). The subfraction Fr. 3.1 was subjected to a Sephadex LH-20 (MeOH) to afford three parts (Fr. 3.1a–Fr. 3.1c). After crude purification of Fr. 3.1a by ODS column (MeOH-H_2_O, 20:80–70:30, *v*/*v*), the second part of Fr. 3.1a (Fr. 3.1a-2, MeOH–H_2_O, 60:40, *v*/*v*) was purified by repeated semi-preparative HPLC (MeCN–H_2_O, 55:45, 2 mL/min) to yield **1** (9.3 mg, *t*_R_ 25.2 min), **2** (6.2 mg, *t*_R_ 28.5 min), **7** (23.6 mg, *t*_R_ 30.1 min).

Three subfractions (Frs. 3.2a–3.2c) were obtained from Fr. 3.2 by chromatography on an ODS column (MeOH–H_2_O, 20:80–70:30, *v*/*v*). Fr.3.2b was chromatographed on ODS (MeOH–H_2_O, 40:60–70:30) to afford a mixture (**4**, **5**, **6**, **8**) in 40% yield. The mixture was purified by semi-preparative HPLC (MeCN–H_2_O, 40:60–60:40, 2 mL/min) to yield **8** (16.3 mg, *t*_R_ 25.5 min, 45:55), **5** (200.0 mg, *t*_R_ 27.2 min, 45:55), **4** (3.7 mg, *t*_R_ 21.0 min, 40:60), and **6** (10.2 mg, *t*_R_ 21.0 min, 40:60). Fr. 3.2c was chromatographed on ODS (MeOH–H_2_O, 30:70–80:20, *v*/*v*) to yield four parts. Fr. 3.2c-2 (the second part of Fr. 3.2c, MeOH–H_2_O, 50:50–60:40, *v*/*v*) was purified by repeated semi-preparative HPLC (MeCN–H_2_O, 46:54, 2 mL/min) to yield **3** (2.78 mg, *t*_R_ 15.1 min).

#### 3.4.1. Asperversin A (**1**)

C_2__4_H_32_O_8_; a white amorphous powder; [*α*]^25^_D_ +46.7 (*c* 0.34, MeOH); UV (MeOH) *λ*_max_ (log *ε*) 205 (4.51) and 286 (3.84) nm; IR (KBr) *ν*_max_ 3445, 2958, 1741, 1710, 1654, 1588, 1444, 1403, 1384, 1237, 1194, 996 cm^−1^; ECD (MeOH) *λ*_max_ (Δ*ε*) = 205 (+25.02) and 286 (+1.86) nm; For ^1^H NMR (400 MHz) and ^13^C NMR (100 MHz) data, see Table 1 and Table 2; HRESIMS 449.2173 ([M + H]^+^, calcd. for C_24_H_33_O_8_, 449.2175).

#### 3.4.2. Asperversin B (**2**)

C_2__3_H_30_O_8_; a white amorphous powder; [*α*]^25^_D_ +100.3 (*c* 0.29, MeOH); UV (MeOH) *λ*_max_ (log *ε*) 202 (4.78) and 285 (4.14) nm; IR (KBr) *ν*_max_ 3433, 2963, 2929, 1740, 1710, 1652, 1584, 1446, 1407, 1235, 1194, 1028 cm^−1^; ECD (MeOH) *λ*_max_ (Δ*ε*) = 207 (+21.37) and 286 (+0.11) nm; For ^1^H NMR (400 MHz) and ^13^C NMR (100 MHz) data, see Table 1 and Table 2; HRESIMS *m/z* 457.1846 ([M + Na]^+^, calcd. for C_23_H_30_NaO_8_, 457.1838).

#### 3.4.3. Asperversin C (**3**)

C_2__5_H_32_O_9_; a white amorphous powder; [*α*]^25^_D_ +100.9 (*c* 0.23, MeOH); UV (MeOH) *λ*_max_ (log *ε*) 205 (4.51) and 284 (3.87) nm; IR (KBr) *ν*_max_ 3425, 2922, 1743, 1711, 1655, 1591, 1233, 1192, 1032 cm^−1^; ECD (MeOH) *λ*_max_ (Δ*ε*) = 203 (+19.88) and 286 (+2.34) nm; For ^1^H NMR (400 MHz) and ^13^C NMR (100 MHz) data, see Table 1 and Table 2; HRESIMS *m/z* 491.2269 ([M + H]^+^, calcd. for C_26_H_35_O_9_, 491.2281).

#### 3.4.4. Asperversin D (**4**)

C_2__1_H_28_O_6_; a white amorphous powder; [*α*]^25^_D_ +98.4 (*c* 0.25, MeOH); UV (MeOH) *λ*_max_ (log *ε*) 205 (4.77) and 286 (3.93) nm; IR (KBr) *ν*_max_ 3429, 2928, 1702, 1653, 1586, 1449, 1408, 1386, 1313, 1274, 1154, 1113 cm^−1^; ECD (MeOH) *λ*_max_ (Δ*ε*) = 205 (+23.13) and 285 (+2.24) nm; For ^1^H NMR (600 MHz) and ^13^C NMR (150 MHz) data, see Table 1 and Table 2; HRESIMS 377.1919 ([M + H]^+^, calcd. for C_22_H_29_O_8_, 377.1964).

#### 3.4.5. Asperversin E (**5**)

C_2__3_H_30_O_8_; colorless crystals; [*α*]^25^_D_ +75.5 (*c* 0.20, MeOH); UV (MeOH) *λ*_max_ (log *ε*) 205 (4.57) and 286 (3.89) nm; IR (KBr) *ν*_max_ 3431, 2988, 2931, 1714, 1653, 1588, 1447, 1402, 1233, 1128, 1086 cm^−1^; ECD (MeOH) *λ*_max_ (Δ*ε*) = 208 (+28.96) and 281 (+1.86) nm; For ^1^H NMR (600 MHz) and ^13^C NMR (150 MHz) data, see Table 1 and Table 2; HRESIMS 457.1830 ([M + Na]^+^, calcd. for C_2__3_H_30_O_8_Na, 457.1838).

#### 3.4.6. Asperversin F (**6**)

C_2__5_H_32_O_9_; a white amorphous powder; [*α*]^25^_D_ +100.6 (*c* 0.48, MeOH); UV (MeOH) *λ*_max_ (log *ε*) 205 (4.61) and 286 (3.77) nm; IR (KBr) *ν*_max_ 3462, 2992, 2934, 1736, 1655, 1590, 1448, 1409, 1374, 1230, 1202, 1131, 1024 cm^−1^; ECD (MeOH) *λ*_max_ (Δ*ε*) = 215 (+23.68) and 282 (+0.34) nm; For ^1^H NMR (400 MHz) and ^13^C NMR (100 MHz) data, see Table 1 and Table 2; HRESIMS 477.2143 ([M + H]^+^, calcd. for C_2__5_H_33_O_9_, 477.2098).

#### 3.4.7. Asperversin G (**7**)

C_2__3_H_28_O_6_; colorless cubic crystals; [*α*]^25^_D_ +110.2 (*c* 0.21, MeOH); UV (MeOH) *λ*_max_ (log *ε*) 205 (4.60), 228 (4.18) and 285 (3.88) nm; IR (KBr) *ν*_max_ 3434, 2975, 2927, 2862, 1793, 1706, 1674, 1587, 1448, 1408, 1257, 1232, 1141 cm^−1^; ECD (MeOH) *λ*_max_ (Δ*ε*) = 215 (+16.60), 291 (+1.11) and 342 (–1.01) nm; For ^1^H NMR (400 MHz) and ^13^C NMR (100 MHz) data, see Table 1 and Table 2; HRESIMS [M + H]^+^ *m/z* 401.1977 (calcd. for C_2__3_H_29_O_6_, 401.1964).

### 3.5. X-ray Crystal Structure Analysis

The crystallographic data for **5** (deposition no. CCDC 1816743), and **7** (deposition no. CCDC 1813401) have been deposited in the Cambridge Crystallographic Data Centre. Copies of the data can be obtained free of charge from the Cambridge Crystallographic Data Centre.

#### 3.5.1. Crystal Data for Asperversin E (**5**)

C_23_H_30_O_8_∙H_2_O (*M* = 452.48 g/mol), orthorhombic, space group *P2_1_2_1_2_1_* (no. 19), *a* = 9.95215(4) Å, *b* = 12.89469(4) Å, *c* = 17.07108(6) Å, *V* = 2190.729(13) Å^3^, *Z* = 4, *T* = 100.01(10) K, *μ* (CuK*α*) = 0.881 mm^−1^, *Dcalc* = 1.372 g/cm^3^, 23,143 reflections measured (8.594 ≤ 2*Θ* ≤ 147.672), 4396 unique (*R*_int_ = 0.0181, *R*_sigma_ = 0.0097) which were used in all calculations. The final *R*_1_ was 0.0274 (I > 2*σ*(I)) and *wR*_2_ was 0.0772. The Flack parameter was 0.039(16).

#### 3.5.2. Crystal Data for Asperversin G (**7**)

C_23_H_28_O_6_ (*M* = 400.45 g/mol), monoclinic, space group *P2_1_*, *a* = 8.77354(16) Å, *b* = 13.09891(17) Å, *c* = 9.64200(17) Å, *α* = 90.0°, *β* = 116.686(2)°, *γ* = 90.0°, *V* = 990.06(3) Å^3^, *Z* = 2, *T* = 100.01(10) K, *μ* (CuK*α*) = 0.790 mm^−1^, *Dcalc* = 1.541 g/cm^3^, 23,143 reflections measured (10.268 ≤ 2*Θ* ≤ 147.12), 3462 unique (*R*_int_ = 0.0195, *R*_sigma_ = 0.0162) which were used in all calculations. The final *R*_1_ was 0.0276 (I > 2*σ*(I)) and *wR*_2_ was 0.0738. The Flack parameter was 0.11(7).

### 3.6. Computational Methods

Monte Carlo conformational searches were carried out by the Spartan’s 10 software using the Merck Molecular Force Field (MMFF). The conformers with a Boltzmann-population of over 5% were chosen for ECD calculations, and then the conformers were initially optimized at B3LYP/6-31+g (d, p) level in MeOH using the CPCM polarizable conductor calculation model. The theoretical calculation of ECD was conducted in MeOH using Time-dependent Density functional theory (TD-DFT) at the B3LYP/6-311+g (d, p) level for all conformers of compounds **1a** and **1b**. Rotatory strengths for a total of 50 excited states were calculated. ECD spectra were generated using the program SpecDis 1.6 (University of Würzburg, Würzburg, Germany) and GraphPad Prism 5 (University of California San Diego, USA) from dipole-length rotational strengths by applying Gaussian band shapes with sigma = 0.3 eV [24].

### 3.7. Acetylcholinesterase Inhibitory Activities Assay

The inhibitory activities of compounds **1**–**8** against AChE were evaluated by Ellman’s method with slight modification [25]. Briefly, 20 µL AChE (0.05 U/mL) and 20 µL different concentrations of compounds (0.1 M phosphate buffer, pH 8.0) containing 0.1% Triton X-100) were pre-incubated at 37 °C for 30 min, then 160 µL of the assay solution which consisted of a 0.1 M phosphate buffer pH 8.0, with the addition of 600 µM 5,5’-dithio-bis (2-nitrobenzoic acid) and 550 µM acetylthiocholine iodide (ATCh) were added in the 96-well plates. The absorbance value at 425 nm was recorded for 15 min and enzyme activity was calculated from the slope of the obtained linear trend. The reaction rates were compared and the % inhibition due to the presence of tested inhibitors was calculated. Each concentration was analyzed in triplicate, and IC_50_ values were determined graphically from log concentration inhibition curves (Graphpad Prism 5, GraphPad Software Inc., San Diego, CA, USA). Galanthamine was used as a standard inhibitor.

### 3.8. Docking Studies

Cocrystal structure of human AChE with compound **1** (PDB code: 4EY7) was obtained from the Protein Data Bank for docking tests (http://www.rcsb.org) [26]. The virtual screening was implemented in the Surflex-Dock module of the Sybyl software. Molecules were built with Chemdraw and optimized at molecular mechanical and semiempirical level by using Open Babel GUI. The crystallographic ligand was extracted from the active site and the designed ligands were modelled. All the hydrogen atoms were added to define the correct ionization and tautomeric states, and the carboxylate, phosphonate and sulfonate groups were considered in their charged form. In the docking calculation, the default FlexX scoring function was used for exhaustive searching, solid body optimization, and interaction scoring. Finally, the ligands with the lowest-energy and the most favorable orientation were selected [27].

## 4. Conclusions

In conclusion, eight meroterpenoids were isolated from the marine derived fungus *Aspergillus versicolor*, including seven new ones, asperversins A–E (**1**–**7**), and a known one, asperdemin (**8**). Notably, asperversins A (**1**) and B (**2**) possess a unique 5/6/6/6 ring system with a constructed tetrahydrofuran ring. The absolute structures of all new compounds were confirmed by comprehensive spectroscopic analyses: compound **1** was elucidated by experimental and calculated ECD, and compounds **5** and **7** were ascertained by the X-ray crystallographic diffractions. All isolated meroterpenoids were tested for their acetylcholinesterase enzyme (AChE) inhibitory activities, and compound **7** displayed moderate inhibitory activity against the AChE with an IC_50_ value of 13.6 μM.

## Figures and Tables

**Table 1:** ^1^H NMR Data for Compounds **1**–**7** (*δ* in ppm, *J* in Hz).

*No.*	1 ^*a*^	2 ^*a*^	3 ^*a*^	4 ^*b*^	5 ^*b*^	6 ^*a*^	7 ^*a*^
1	3.98, dd (9.6, 2.3)	3.98, dd (9.7, 2.0)	5.42, dd (11.0, 1.8)	3.87, t (7.0)	3.79, dd (7.0, 5.2)	4.96, d (7.3)	7.08, d (10.2)
2	2.46, dd (15.7, 9.6) 2.68, m	2.39, m 2.63, dd (15.7, 2.0)	2.43, dd (14.8, 3.9) 2.88, dd (14.8, 1.8)	2.84, m 3.33, m	2.79, dd (16.0, 7.0) 3.32, d (16.0)	3.10, dd (16.2, 7.3) 3.55, d (16.2)	5.87, d (10.2)
5	2.13, d (2.3)	2.12, d (2.5)	2.49, d (3.0)	2.21, dd (12.1, 2.4)	2.38, d (2.3)	2.49, d (2.6)	1.98, d (1.8)
6	5.48, dd (4.5, 2.3)	5.48, m	5.27, m	1.67, m 1.92, m	5.52, dd (6.3, 3.0)	5.64, m	5.60, m
7	2.11, m 2.35, m	2.11, m 2.35, m	2.04, m 2.30, dd (14.4, 2.9)	1.72, m 2.06, m	1.92, dd, (14.6, 6.3) 2.26, dd, (14.6, 3.0)	2.05, m 2.40, dd (14.7, 3.0)	1.91, dd (14.7, 3.0) 2.42, m
9	1.97, dd (12.6, 5.2)	1.98, dd (11.3, 6.4)	1.90, dd (12.7, 4.6)	2.32, dd (12.5, 4.6)	2.34, m	2.15, m	1.81, dd (12.9, 4.3)
11	2.33, m 2.41, m	2.40, m 2.41, m	2.40, m 2.80, dd (17.4, 4.6)	2.08, m 2.43, dd (16.2, 4.6)	2.09, m 2.35, m	2.29, m 2.30, m	2.42, dd (16.5, 12.9) 2.76, dd (16.5, 4.3)
15	5.91, d (1.0)	5.92, d (1.1)	5.91, d (1.1)	5.81, d (1.1)	5.72, d (0.6)	5.91, d (1.1)	5.70, d (1.1)
17	2.20, d (1.0)	2.21, d (1.1)	2.21, d (1.1)	2.15, d (1.1)	2.05, d (0.6)	2.20, d (1.1)	2.18, d (1.1)
18	1.43, s	1.43, s	1.42, s	1.30, s	1.32, s	1.41, s	1.40, s
19	1.31, s	1.32, s	1.33, s	1.14, s	1.35, s	1.53, s	1.46, s
20	1.26, s	1.26, s	5.05, m 5.08, m	1.41, s	1.31, s	1.49, s	1.23, s
21	1.18, s	1.18, s	1.94, s	1.50, s	1.57, s	1.73, s	1.19, s
23	2.07, s	2.07, s	2.07, s		2.06, s	2.14, s	2.09, s
24	3.70, s		2.08, s				
25						1.99, s	

*^a^* Recorded at 400 MHz in methanol-*d*_4_; *^b^* recorded at 600 MHz in acetone-*d*_6_.

**Table 2:** ^13^C and DEPT NMR Data of Compounds **1**–**7** (*δ* in ppm, *J* in Hz).

*No.*	1 ^*a*^	2 ^*a*^	3 ^*a*^	4 ^*b*^	5 ^*b*^	6 ^*a*^	7 ^*a*^
1	84.0, CH	84.5, CH	77.2, CH	68.8, CH	69.3, CH	72.6, CH	155.1, CH
2	37.3, CH_2_	37.9, CH_2_	36.2, CH_2_	39.8, CH_2_	39.9, CH_2_	36.3, CH_2_	125.1, CH
3	173.6, C	176.7, C	173.2, C	171.2, C	171.0, C	172.9, C	203.2, C
4	79.7, C	79.6, C	144.4, C	84.8, C	84.1, C	86.7, C	45.1, C
5	61.4, CH	61.5, CH	51.3, CH	50.2, CH	49.5, CH	51.3, CH	53.0, CH
6	68.3, CH	68.3, CH	73.4, CH	23.3, CH_2_	72.6, CH	72.7, CH	68.8, CH
7	45.3, CH_2_	45.3, CH_2_	43.3, CH_2_	40.6, CH_2_	43.3, CH_2_	43.3, CH_2_	43.4, CH_2_
8	81.5, C	81.6, C	81.2, C	80.9, C	79.9, C	80.4, C	79.3, C
9	48.1, CH	48.1, CH	44.2, CH	44.8, CH	44.4, CH	45.1, CH	46.9, CH
10	46.5, C	46.4, C	45.6, C	45.3, C	45.4, C	45.1, C	39.4, C
11	19.4, CH_2_	19.4, CH_2_	19.7, CH_2_	17.5, CH_2_	17.2, CH_2_	17.6, CH_2_	16.9, CH_2_
12	98.5, C	98.6, C	99.0, C	98.2, C	98.3, C	98.3, C	97.3, C
13	167.1, C	167.2, C	167.1, C	164.5, C	164.5, C	164.6, C	164.8, C
14	162.2, C	162.2, C	162.2, C	160.8, C	161.0, C	162.3, C	160.5, C
15	102.2, CH	102.0, CH	101.8, CH	100.6, CH	100.6, CH	101.7, CH	100.3, CH
16	165.1, C	165.2, C	164.8, C	163.3, C	162.8, C	166.8, C	162.7, C
17	19.5, CH_3_	19.5, CH_3_	19.5, CH_3_	19.5, CH_3_	19.5, CH_3_	19.5, CH_3_	19.7, CH_3_
18	23.0, CH_3_	23.0, CH_3_	22.1, CH_3_	20.9, CH_3_	21.9, CH_3_	22.0, CH_3_	22.0, CH_3_
19	12.4, CH_3_	12.5, CH_3_	15.3, CH_3_	15.2, CH_3_	16.2, CH_3_	16.0, CH_3_	18.1, CH_3_
20	30.5, CH_3_	30.6, CH_3_	117.7, CH_2_	34.9, CH_3_	33.9, CH_3_	33.9, CH_3_	26.6, CH_3_
21	25.5, CH_3_	25.6, CH_3_	26.5, CH_3_	24.0, CH_3_	26.4, CH_3_	26.2, CH_3_	22.8, CH_3_
22	171.6, C	171.7, C	171.5, C		170.2, C	171.2, C	169.7, C
23	21.3, CH_3_	21.3, CH_3_	20.9, CH_3_		21.4, CH_3_	21.3, CH_3_	21.5, CH_3_
24	52.3, OCH_3_		172.2, C			171.1, C	
25			21.4, CH_3_			20.6, CH_3_	

*^a^* Recorded at 100 MHz in methanol-*d*_4_; *^b^* recorded at 150 MHz in acetone-*d*_6_.

**Table 3:** The inhibitory value and docking score of compounds **1**–**8** with AChE.

Compounds	Inhibitory Ratio at 100 μM	IC_50_ Value (μM) *^a^*	Docking Score *^b^*
**1**	35.7%	>40	3.25
**2**	36.2%	>40	3.89
**3**	27.6%	>40	2.76
**4**	50.2%	>40	5.32
**5**	48.7%	>40	4.98
**6**	62.9%	>40	5.87
**7**	92.4%	13.6	7.85
**8**	25.3%	>40	2.58
Galanthamine		3.57	

*^a^* IC_50_ value of compounds against AChE (μM); *^b^* Docking score/interaction potential of compounds with target protein.

## Supplementary Materials

The following are available online at http://www.mdpi.com/1660-3397/16/6/177/s1, HRESIMS, 1D and 2D NMR, IR, and UV spectra of **1**–**7**.
