# Supplementary material for: Asperversins A and B, Two Novel Meroterpenoids with an Unusual 5/6/6/6 Ring from the Marine-Derived Fungus Aspergillus versicolor
Source: Mar Drugs. 2018 May 23;16(6):177. doi: 10.3390/md16060177 (PMC6025407; doi:10.3390/md16060177)
Supplement: Supplementary file 1 [file marinedrugs-16-00177-s001.pdf]

Supporting information

Asperversins A and B, two novel meroterpenoids with an unusual 5/6/6/6 ring from the marine-derived fungus *Aspergillus versicolor*

Huaqiang Li [1], Weiguang Sun [1], Mengyi Deng [1], Changxing Qi [1], Chunmei Chen [1], Hucheng Zhu [1], Zengwei Luo [1], Jianping Wang [1,*], Yongbo Xue [1,*] and Yonghui Zhang[1,*]

1 Hubei Key Laboratory of Natural Medicinal Chemistry and Resource Evaluation, Tongji Medical College, Huazhong University of Science and Technology, Wuhan 430030, China;

* Correspondence: jpwang1001@163.com (J. W.); yongboxue@hust.edu.cn (Y. X.); zhangyh@mails.tjmu.edu.cn (Y. Z.)
Tel.: +86-027-83692892 (Y.Z.)

content

Cytotoxic Assay..3

Antifungal Bioassay..4

Figure S1−S9. HRESIMS, NMR, IR and UV spectra of asperversin A (**1**)..............................5

Figure S10−S15. NMR recorded in DMSO-d6 of asperversin A (**1**)..9

Figure S16−S24. HRESIMS, NMR, IR and UV spectra of asperversin B (**2**)........................12

Figure S25−S33. HRESIMS, NMR, IR and UV spectra of asperversin C (**3**)........................17

Figure S34−S42. HRESIMS, NMR, IR and UV spectra of asperversin D (**4**)........................21

Figure S43−S51. HRESIMS, NMR, IR and UV spectra of asperversin E (**5**)........................26

Figure S52−S59. HRESIMS, NMR and IR spectra of asperversin F (**6**)................................30

Figure S60−S68. HRESIMS, NMR, IR and UV spectra of asperversin G (**7**)........................34

Cytotoxic Assay

Four human cancer cell lines (A549, MCF-7, HepG2, and HL-60 cells), together with one noncancerous cell line, the human bronchial epithelial cells Beas-2B, were used in the cytotoxic activity assay. All cells were cultured in DMEM or RPMI- 1640 medium (HyClone, Logan, UT, USA), supplemented with 10% fetal bovine serum (HyClone) at 37 ℃ in a humidified atmosphere with 5% CO_2. The cell survival assay was performed using the MTT method. Briefly, 100 μL of a suspension of the adherent cells was seeded into each well of the 96-well culture plates and allowed to adhere for 12 h before addition of the test compounds. The suspended cells were seeded at an initial density of 1×10^5 cells/mL immediately before the addition of the drug. Each tumor cell line was exposed for 48 h in triplicate to the test compounds at concentrations of 0.0625, 0.32, 1.6, 8, and 40 μM, with DDP (cis-platin, Sigma) and paclitaxel as positive controls. After incubation, culture supernatants were removed and exchanged with medium containing 0.5 mg/mL MTT. Then, the cells were incubated for 4 h at 37 ^{o}C in darkness, followed by the removal of the medium and adding 100 μL dimethyl sulfoxide. The absorbance at 570 nm was detected and the data were expressed as the mean percentage of absorbance in treated vs. control cells. The value of the control was set at 100%.

Table S1. Cytotoxicities against Tumor Cells for **1**–**8** (IC_{50}, μM).

cells / No.	A549	MCF-7	HepG2	HL-60	Beas-2B
**1**	>40	>40	>40	>40	>40
**2**	>40	>40	>40	>40	>40
**3**	>40	>40	>40	>40	>40
**4**	>40	>40	>40	>40	>40
**5**	>40	>40	>40	>40	>40
**6**	>40	>40	>40	>40	>40
**7**	>40	>40	>40	>40	>40
**8**	>40	>40	>40	>40	>40
Adriamycin[a]	0.12	0.79	0.13	0.05	0.21

[a]Positive control.

Antifungal Bioassay

The tested pathogenic fungi, *Staphylococcus aureus* (MRSA), methicillin-sensitive *Staphylococcus aureus* (MSSA), *Escherichia coli* and *Pseudomonas aeruginosa*, were provided by Tongji Hospital of Tongji Medical College of Huazhong University of Science and Technology. All samples dissolved in MeOH were tested for antifungal activity in vitro by mycelial growth inhibitory rate method, a *Poison Food Technique*. Potato dextrose agar (PDA) medium was used as the medium for all test fungi.

The media (100 ml) incorporating test samples were inoculated at the center of agar discs of the test fungi (4 mm diameter). Three replicate plates for each fungus were incubated at 26 °C for all test fungi. Control plates containing media mixed with MeOH (1 ml) were included. After incubation for 24–48 h, the mycelial growth of fungi (mm) in both treated (T) and control (C) *Petri* dishes was measured diametrically in three different directions (decussation method) until the fungal growth in the control dishes was almost complete. The percentage of growth inhibition (*I*) was calculated using the following formula:

$$I\ [\%] = (C - T)/C \times 100$$

Table S2. Antifungal Effects of Compounds **1**−**8** (MIC, μg/ml).

fungi / No.	MRSA	MSSA	*E. coli*	*P. aeruginosa*
**1**	>100	>100	>100	>100
**2**	>100	>100	>100	>100
**3**	>100	>100	>100	>100
**4**	>100	>100	>100	>100
**5**	>100	>100	>100	>100
**6**	>100	>100	>100	>100
**7**	>100	>100	>100	>100
**8**	>100	>100	>100	>100
vancomycin[a]	1.2	0.5	0.5	0.8

[a]Positive control.

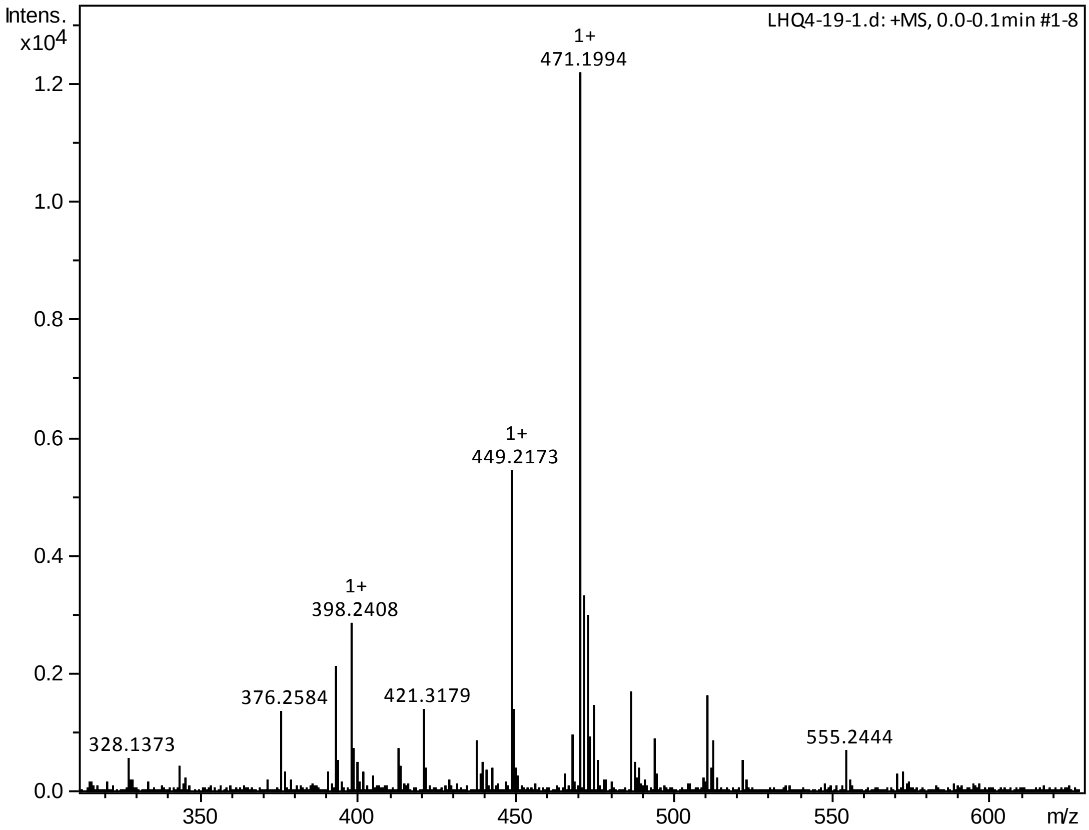

Figure S1. HRESIMS spectrum of asperversin A (**1**).

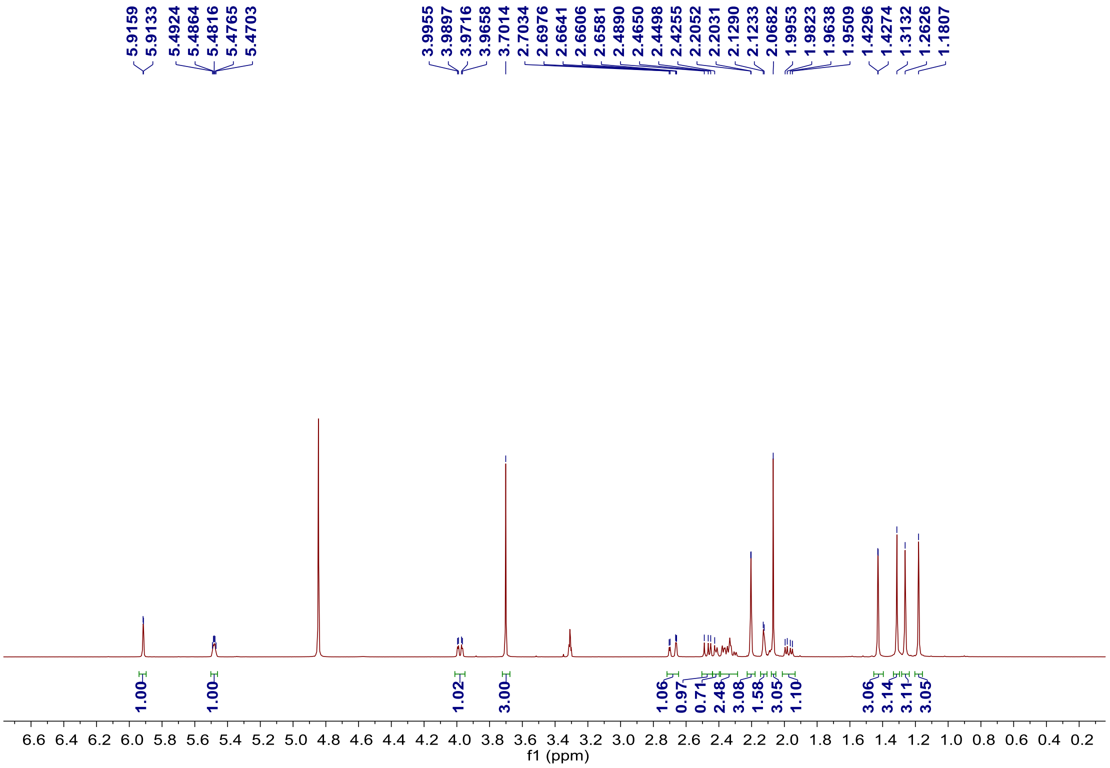

Figure S2. ^{1}H NMR spectrum of asperversin A (**1**) (400 MHz, methanol-d_4).

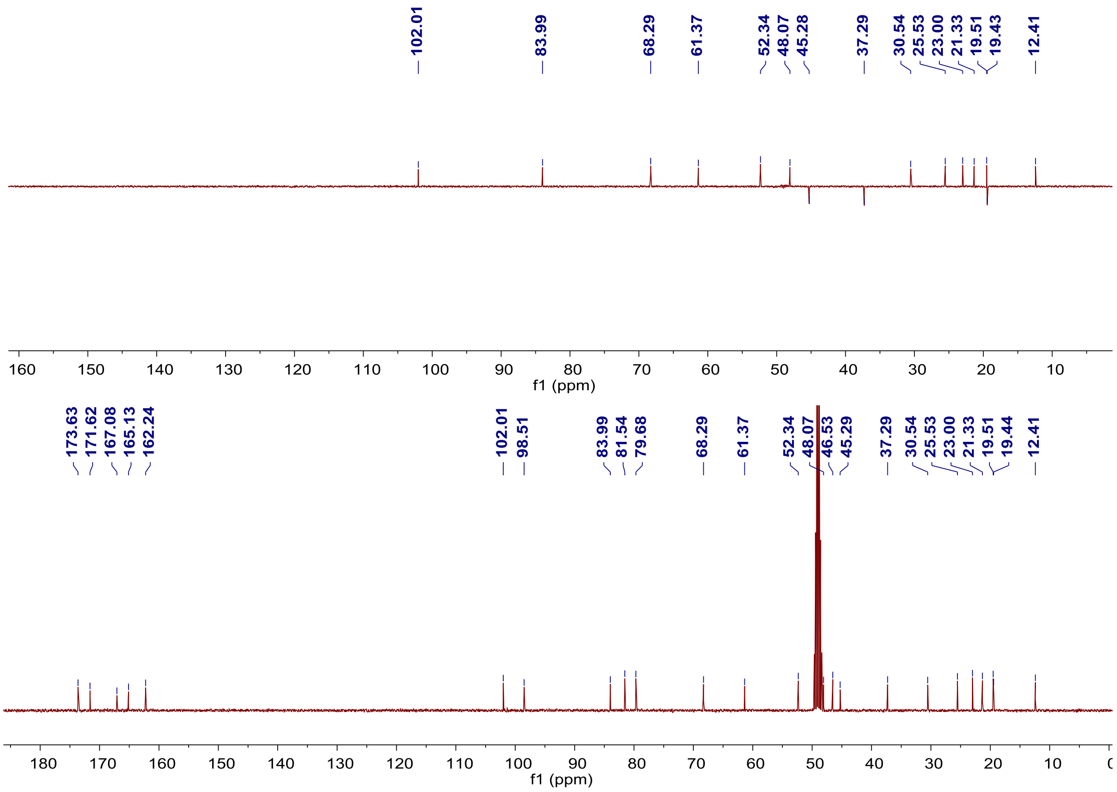

Figure S3. ^{13}C NMR and DEPT spectra of asperversin A (**1**) (100 MHz, methanol-d_4).

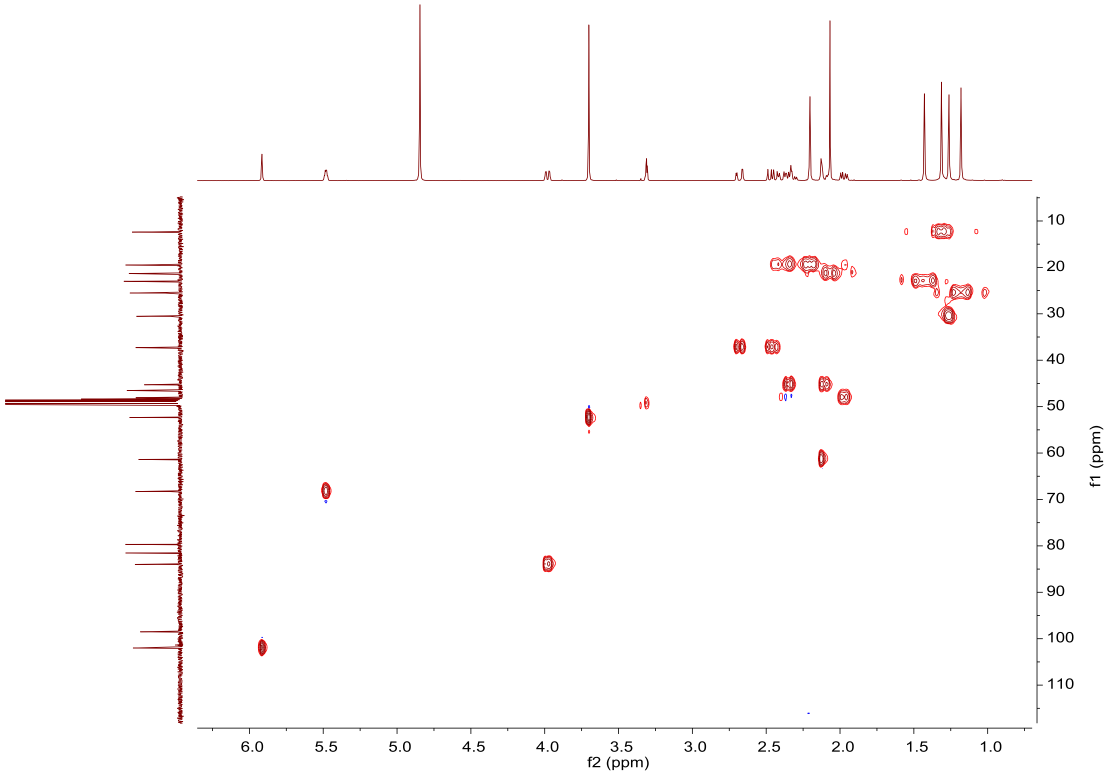

Figure S4. HSQC spectrum of asperversin A (**1**) (methanol-d_4).

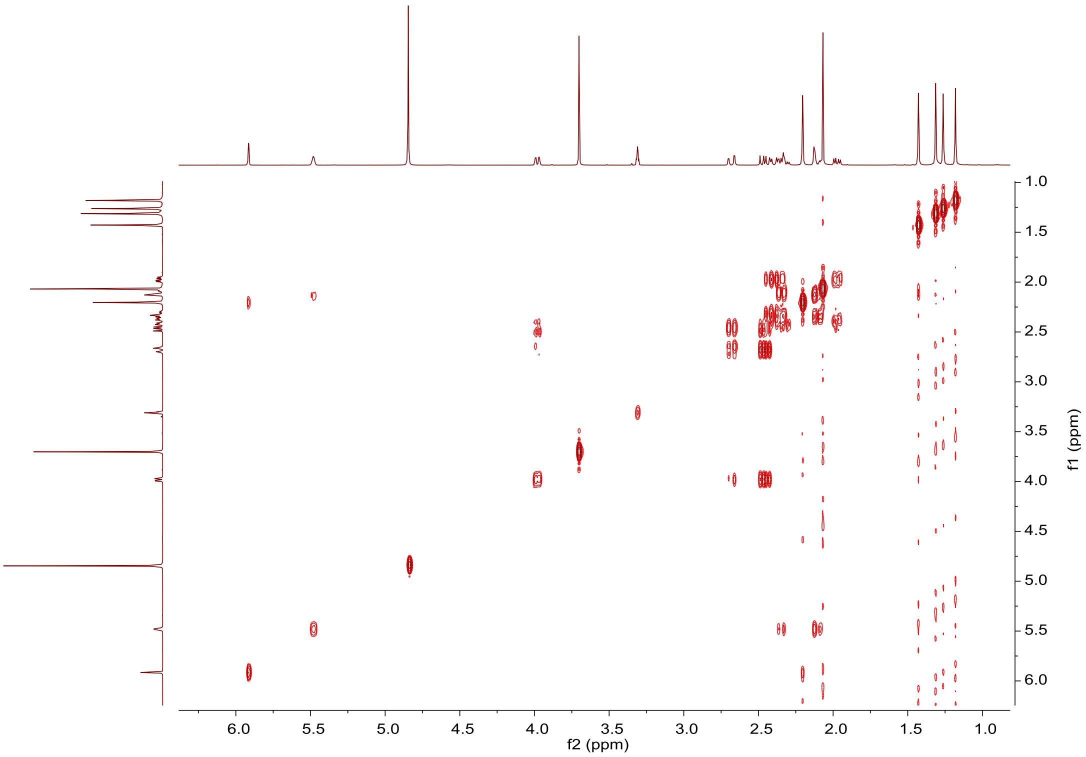

Figure S5. ^{1}H–^{1}H COSY spectrum of asperversin A (**1**) (methanol-*d*4).

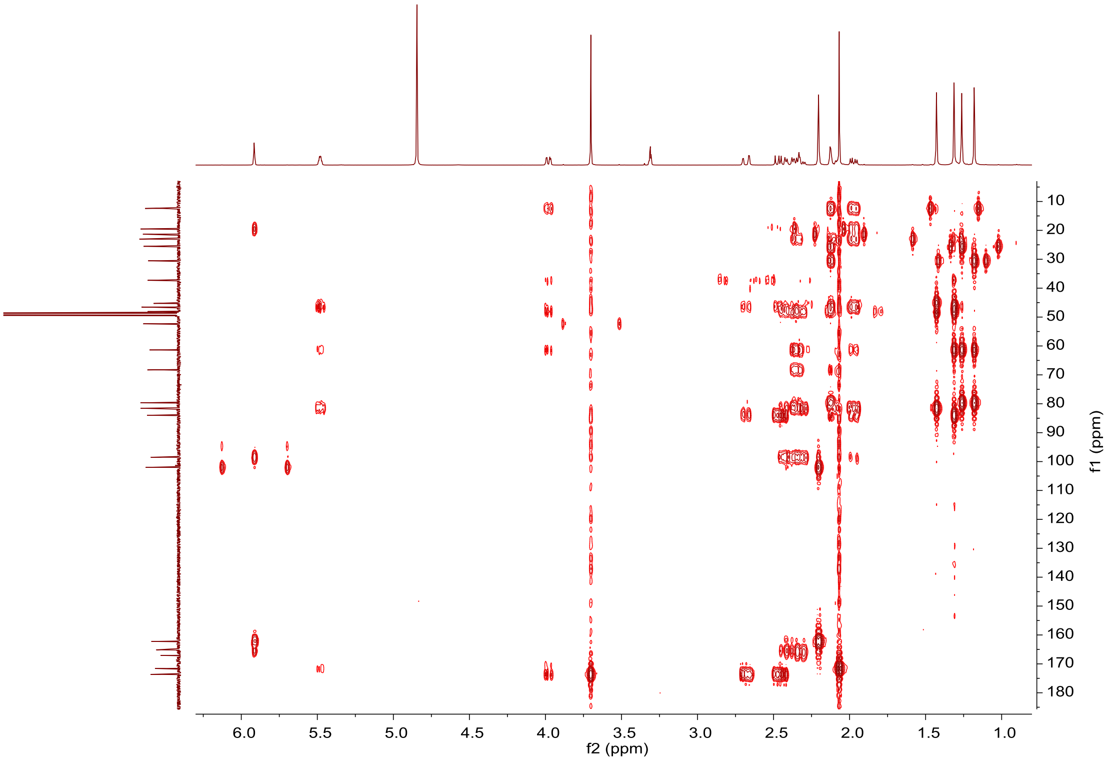

Figure S6. HMBC spectrum of asperversin A (**1**) (methanol-*d*4).

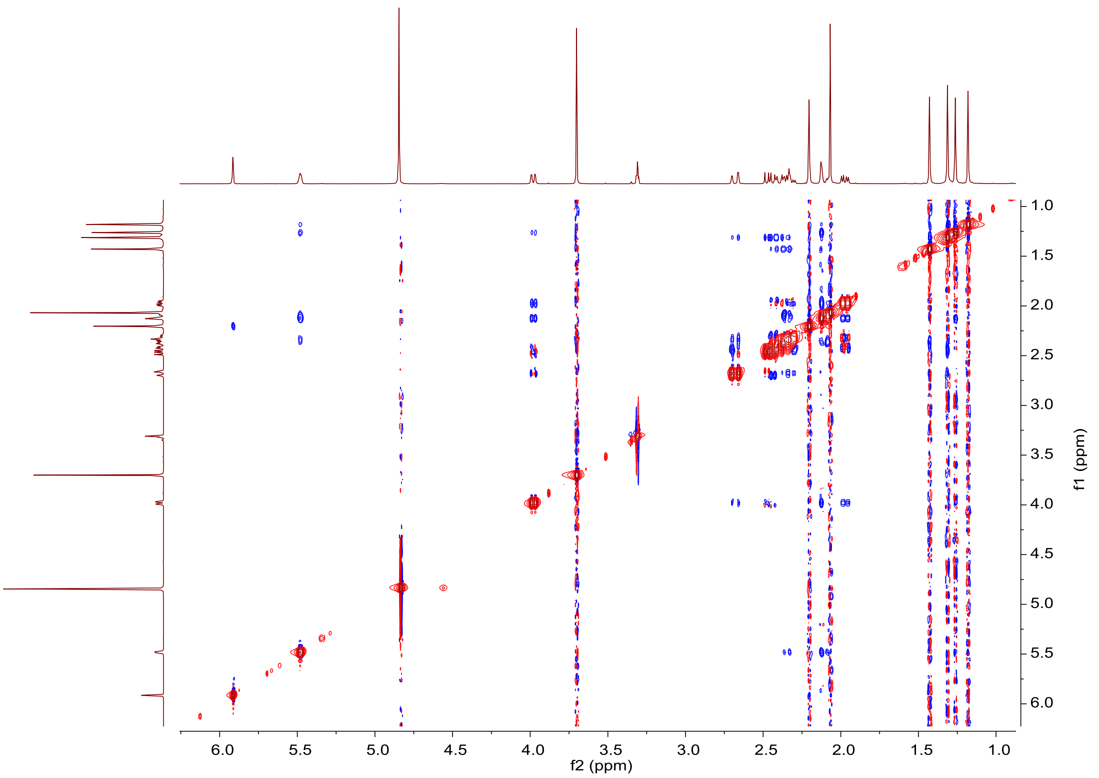

Figure S7. NOESY spectrum of asperversin A (**1**) (methanol-*d*4).

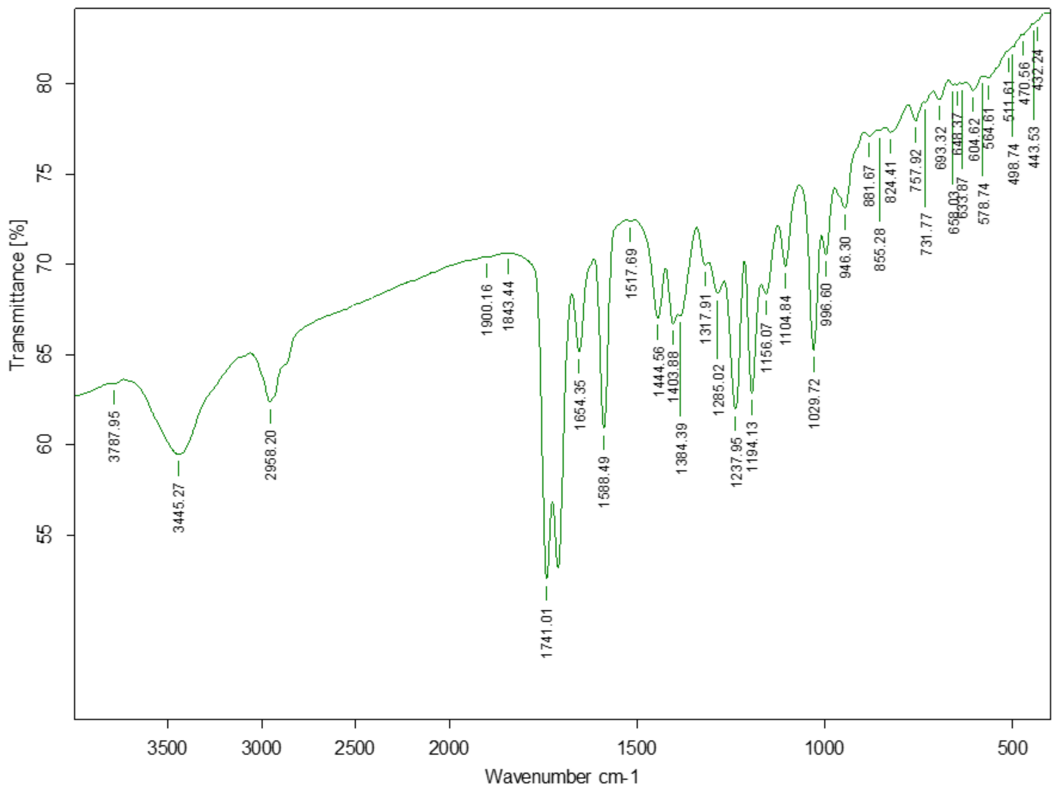

Figure S8. IR spectrum of asperversin A (**1**).

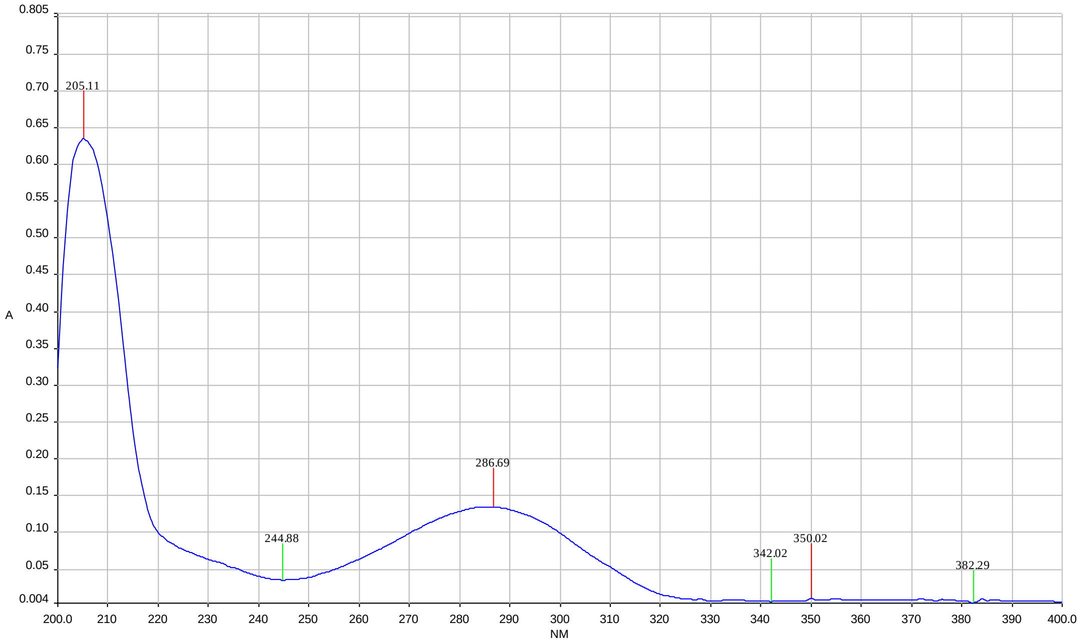

Figure S9. UV spectrum of asperversin A (**1**).

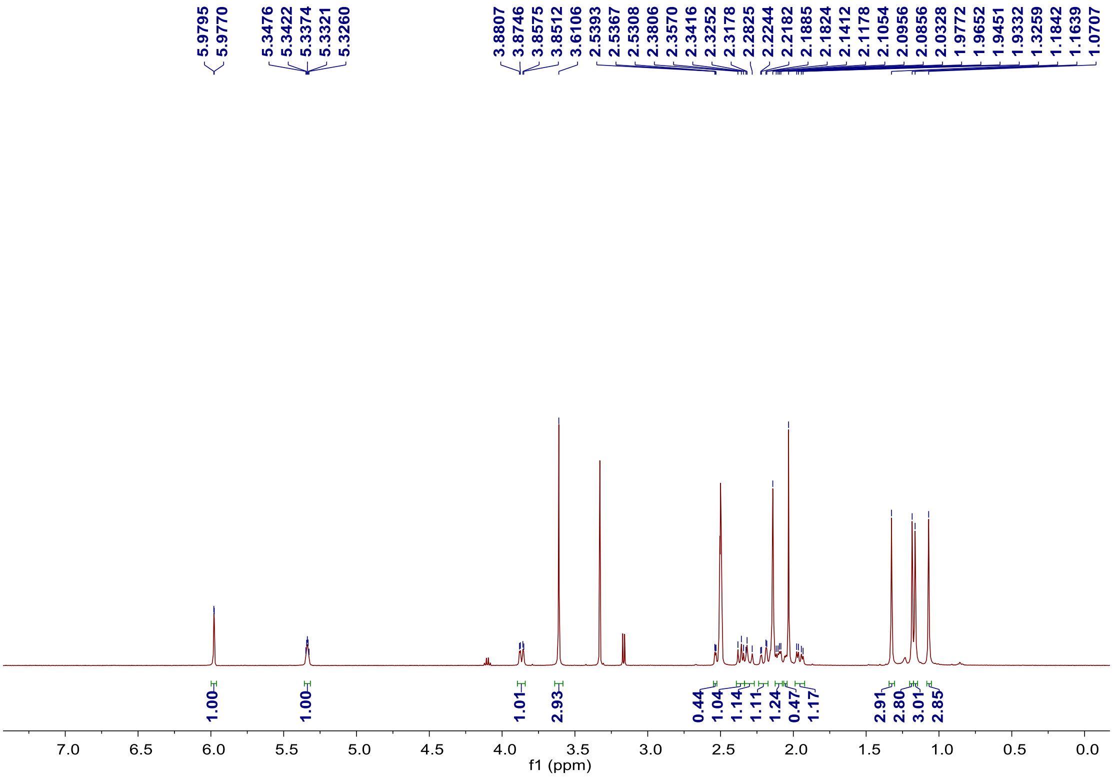

Figure S10. ^{1}H NMR spectrum of asperversin A (**1**) (400 MHz, DMSO-d_6).

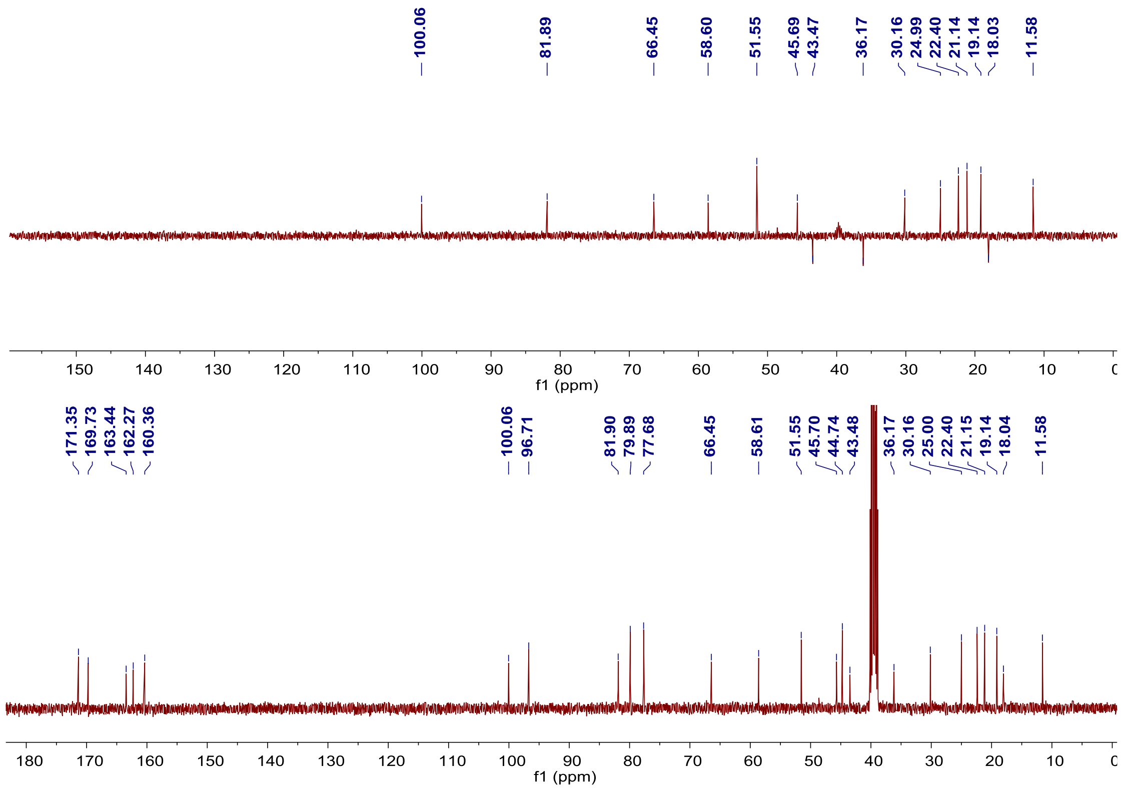

Figure S11. ^{13}C NMR and DEPT spectra of asperversin A (**1**) (100 MHz, DMSO-d_6).

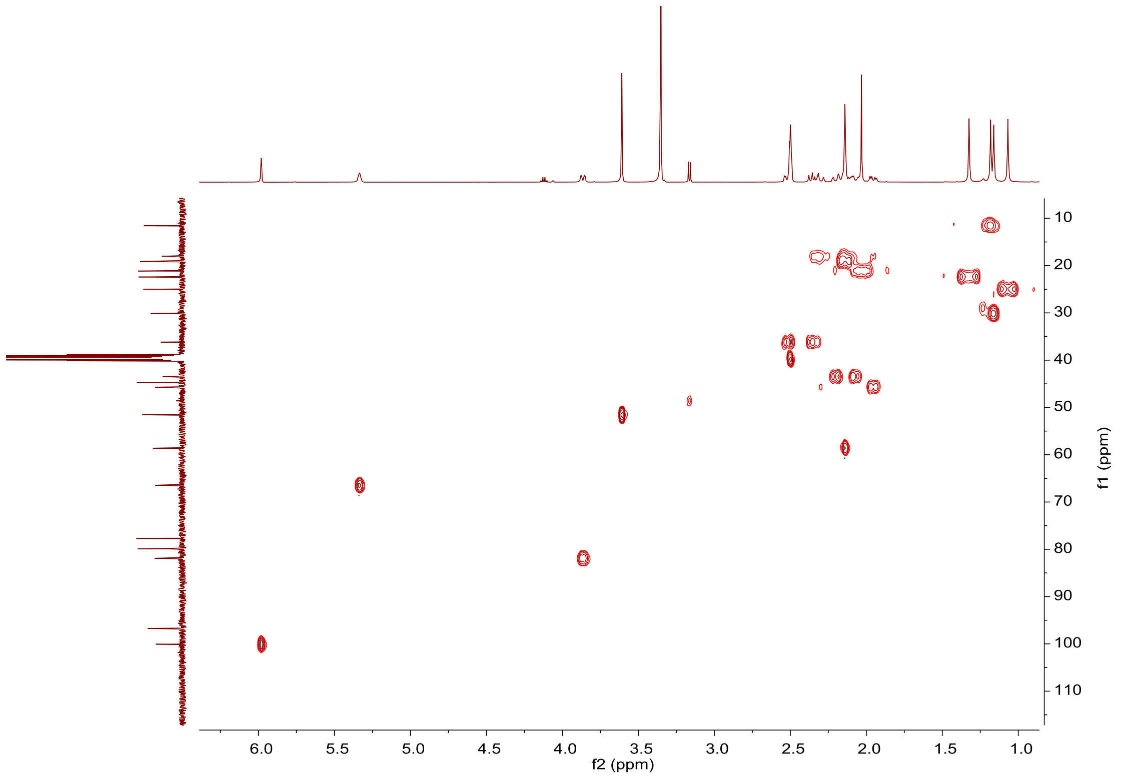

Figure S12. HSQC spectrum of asperversin A (**1**) (DMSO-d_6).

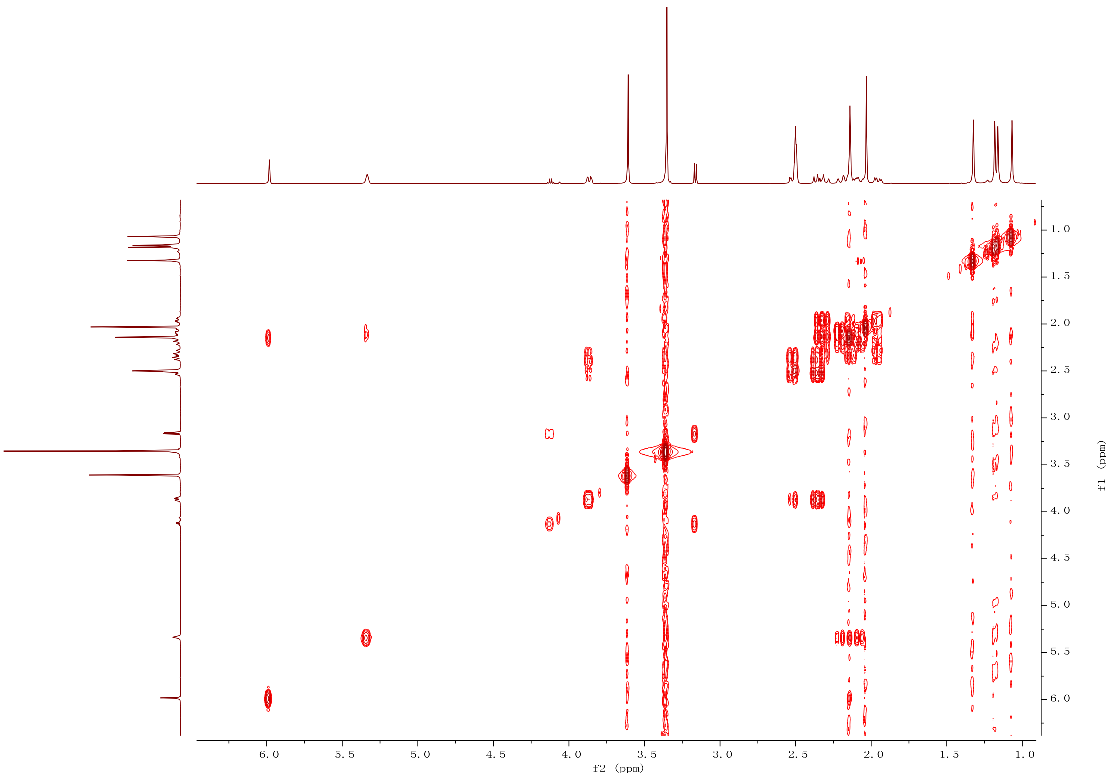

Figure S13. $^{1}H-^{1}H$ COSY spectrum of asperversin A (**1**) (DMSO-d_6).

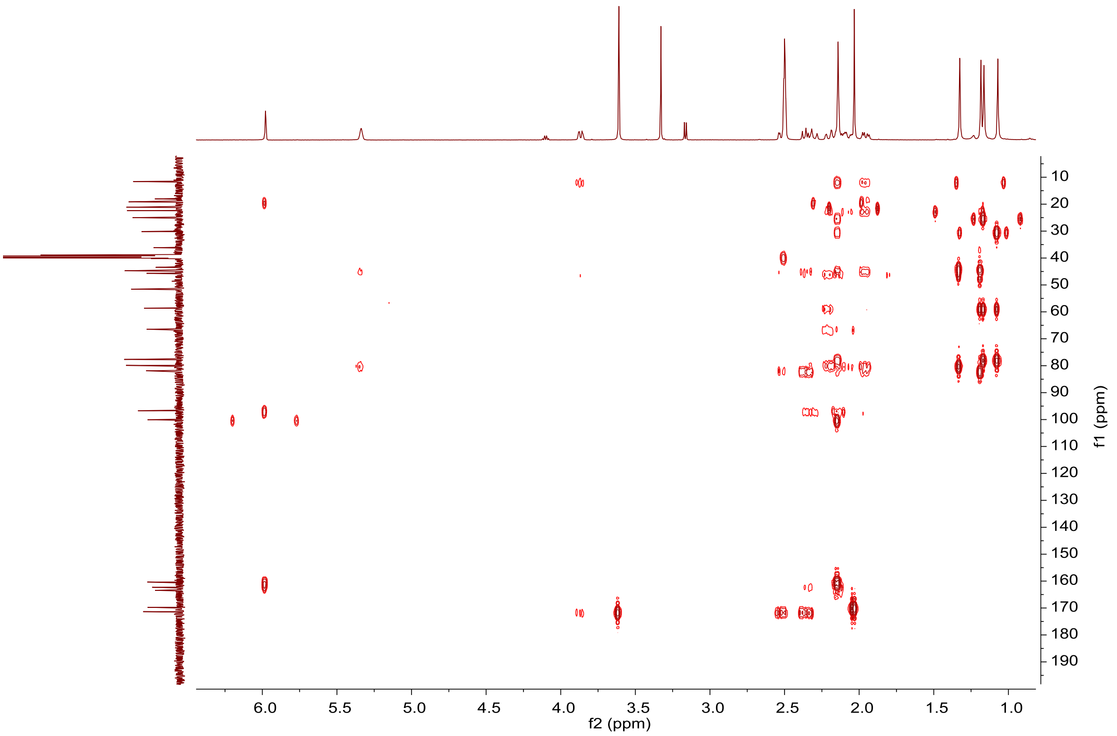

Figure S14. HMBC spectrum of asperversin A (**1**) (DMSO-d_6).

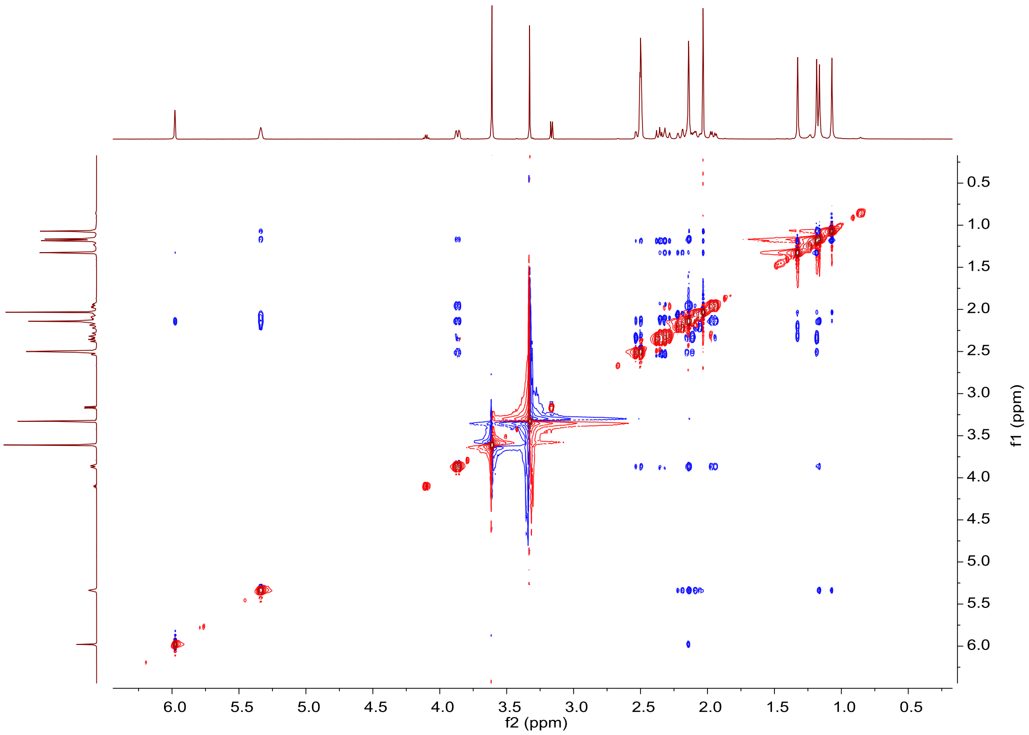

Figure S15. NOESY spectrum of asperversin A (**1**) (DMSO-d_6).

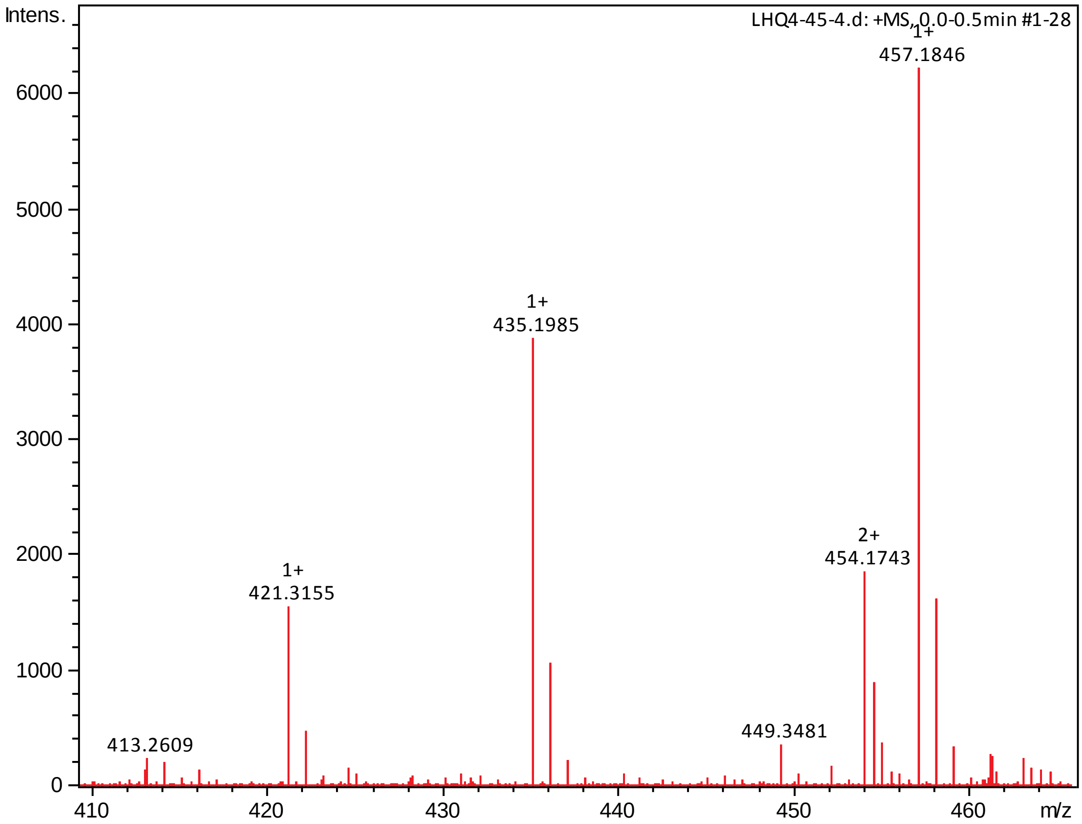

Figure S16. HRESIMS spectrum of asperversin B (**2**).

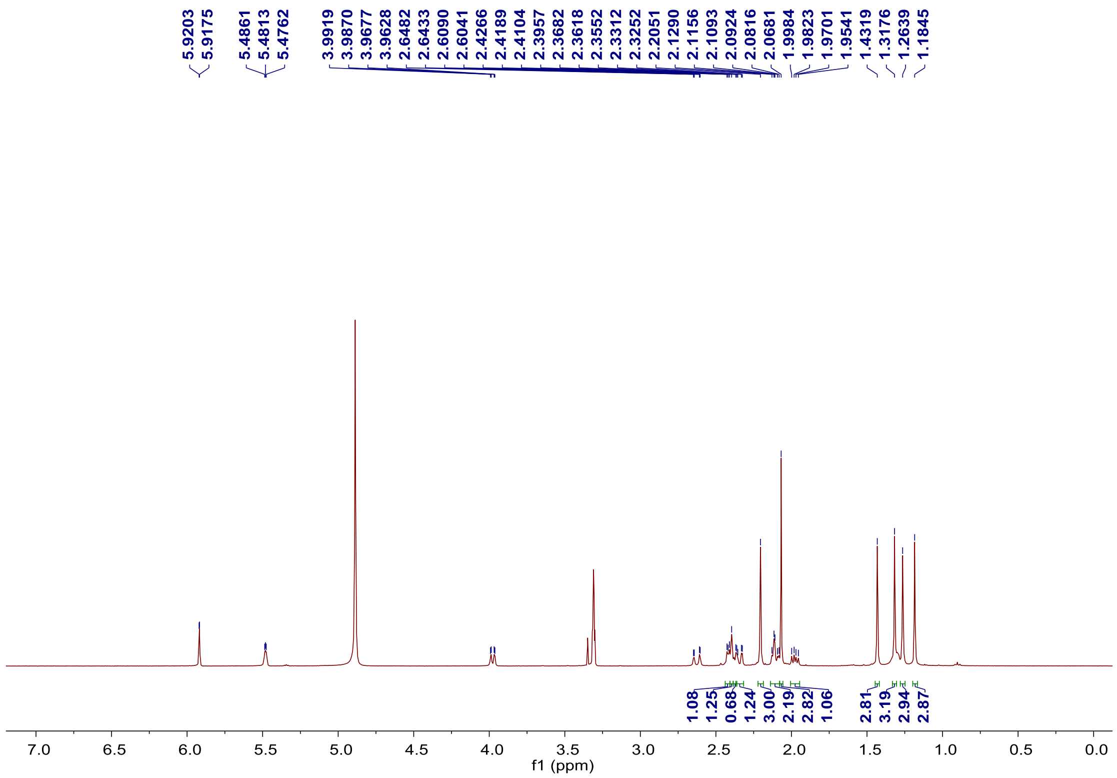

Figure S17. ^{1}H NMR spectrum of asperversin B (**2**) (400 MHz, methanol-d_4).

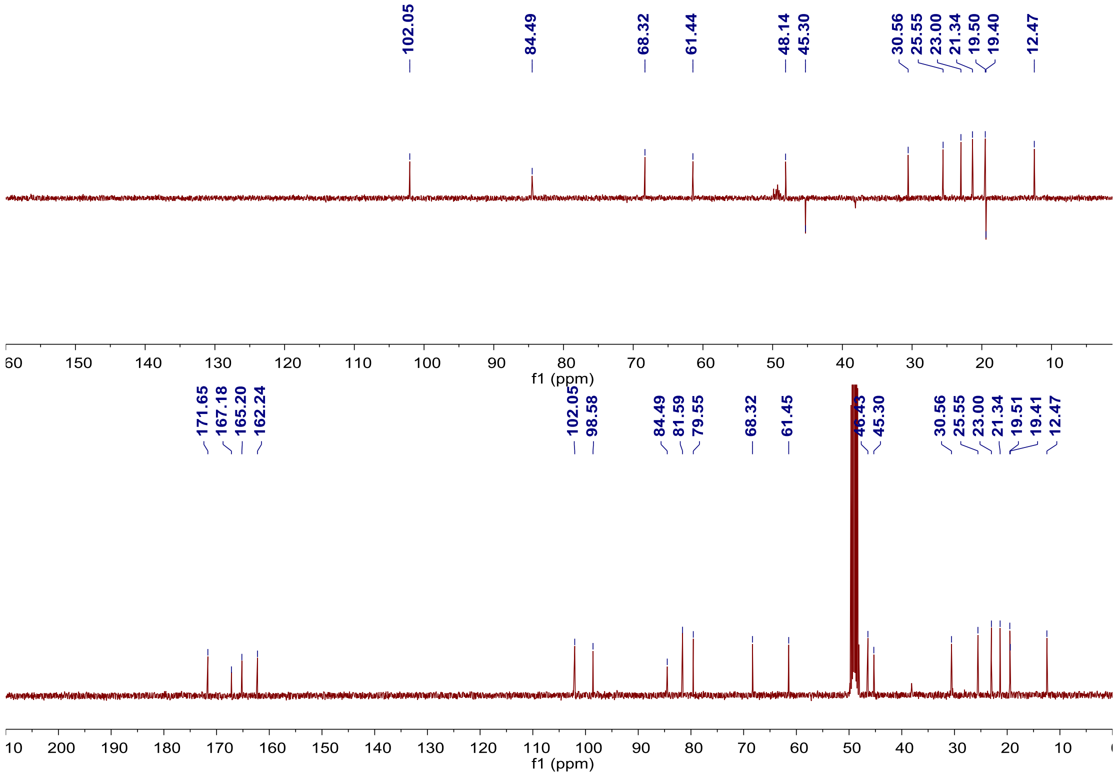

Figure S18. ^{13}C NMR and DEPT spectra of asperversin B (**2**) (100 MHz, methanol-d_4).

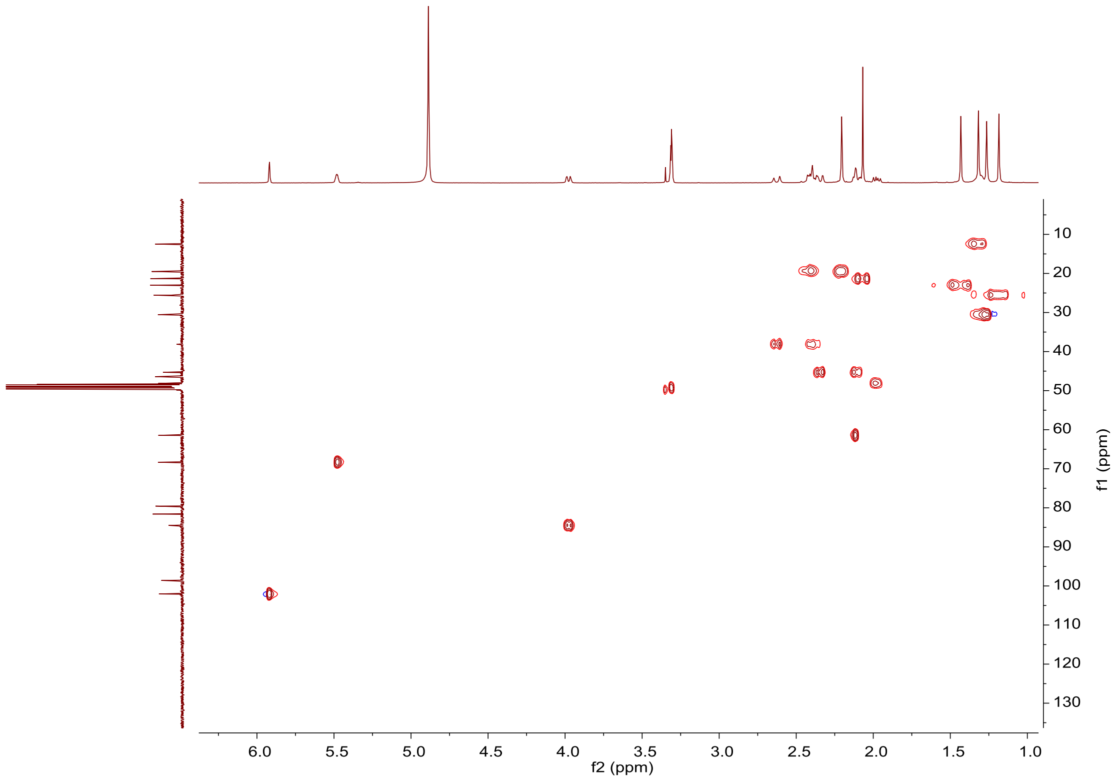

Figure S19. HSQC spectrum of asperversin B (**2**) (methanol-*d*4).

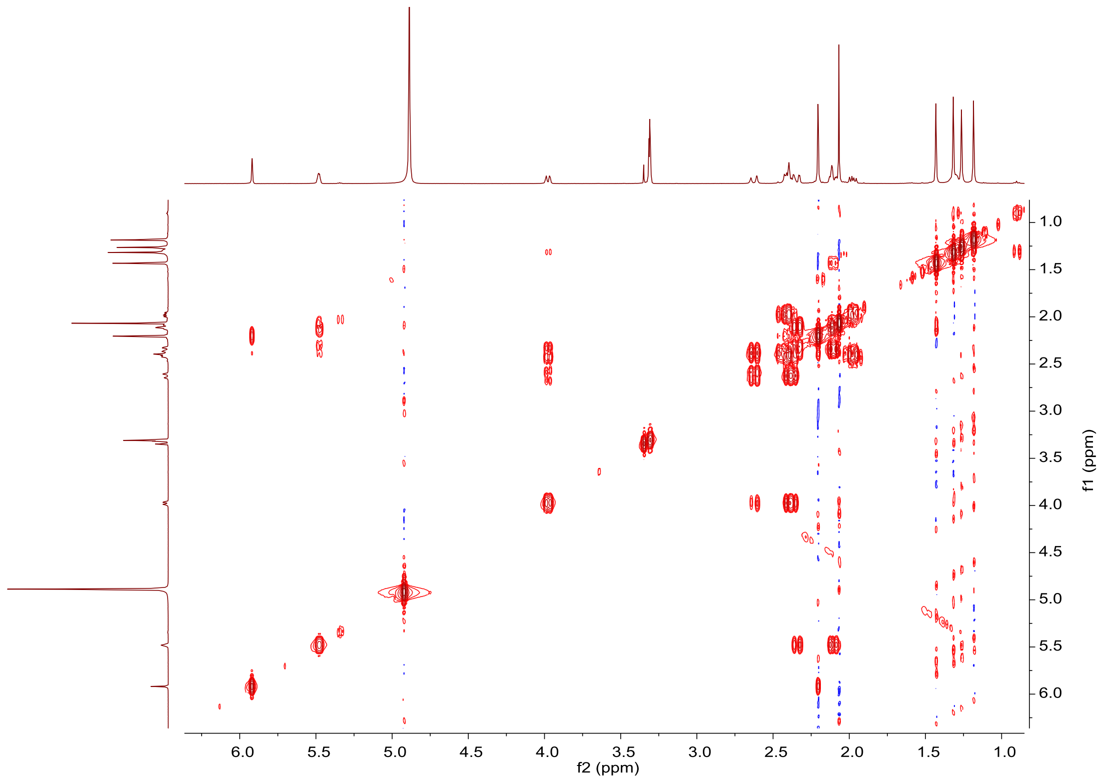

Figure S20. $^{1}H-^{1}H$ COSY spectrum of asperversin B (**2**) (methanol-*d*4).

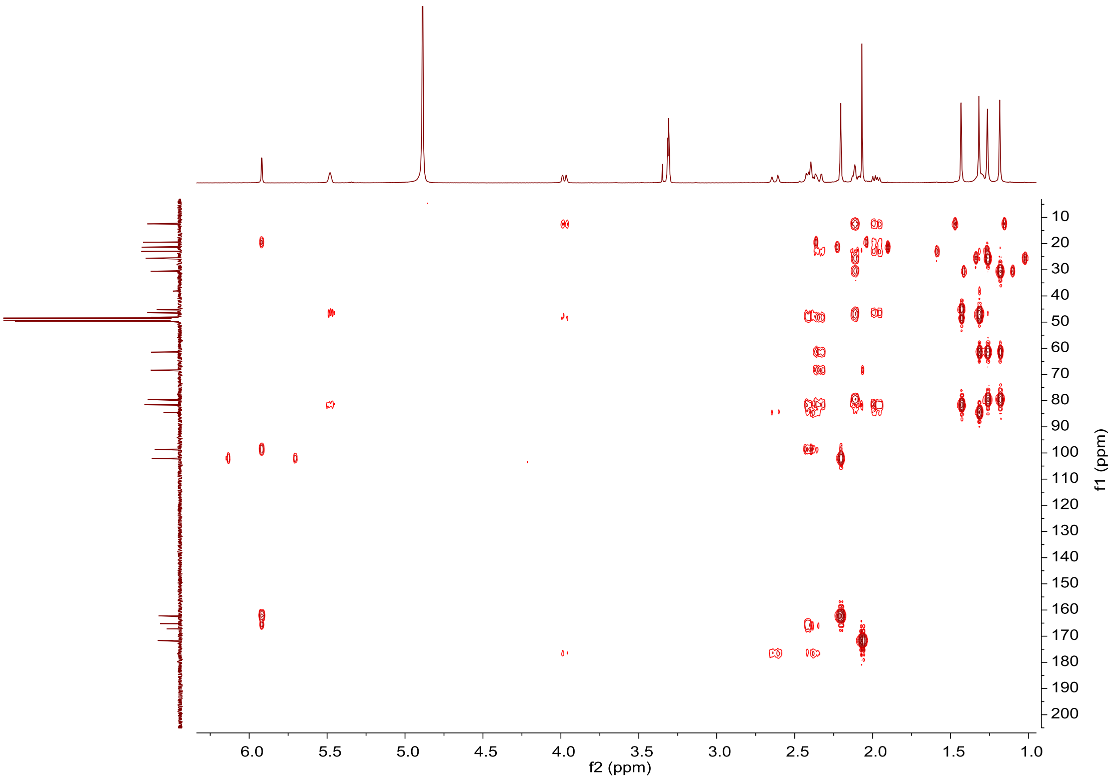

Figure S21. HMBC spectrum of asperversin B (**2**) (methanol-d_4).

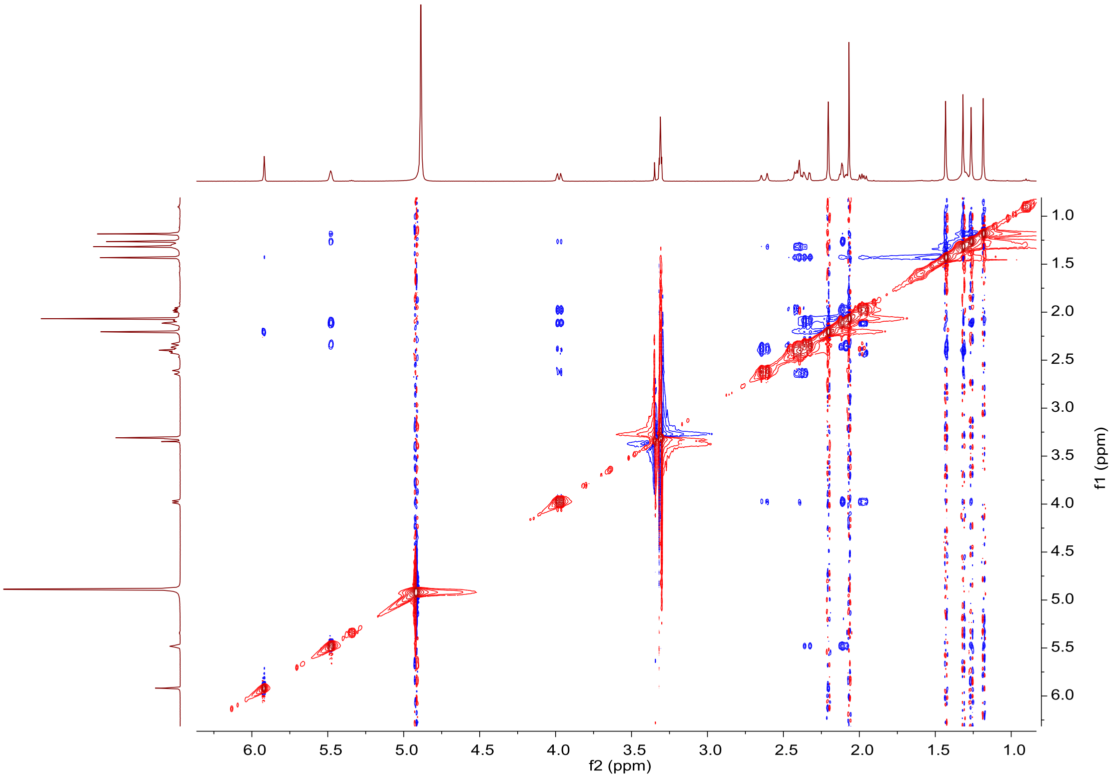

Figure S22. NOESY spectrum of asperversin B (**2**) (methanol-d_4).

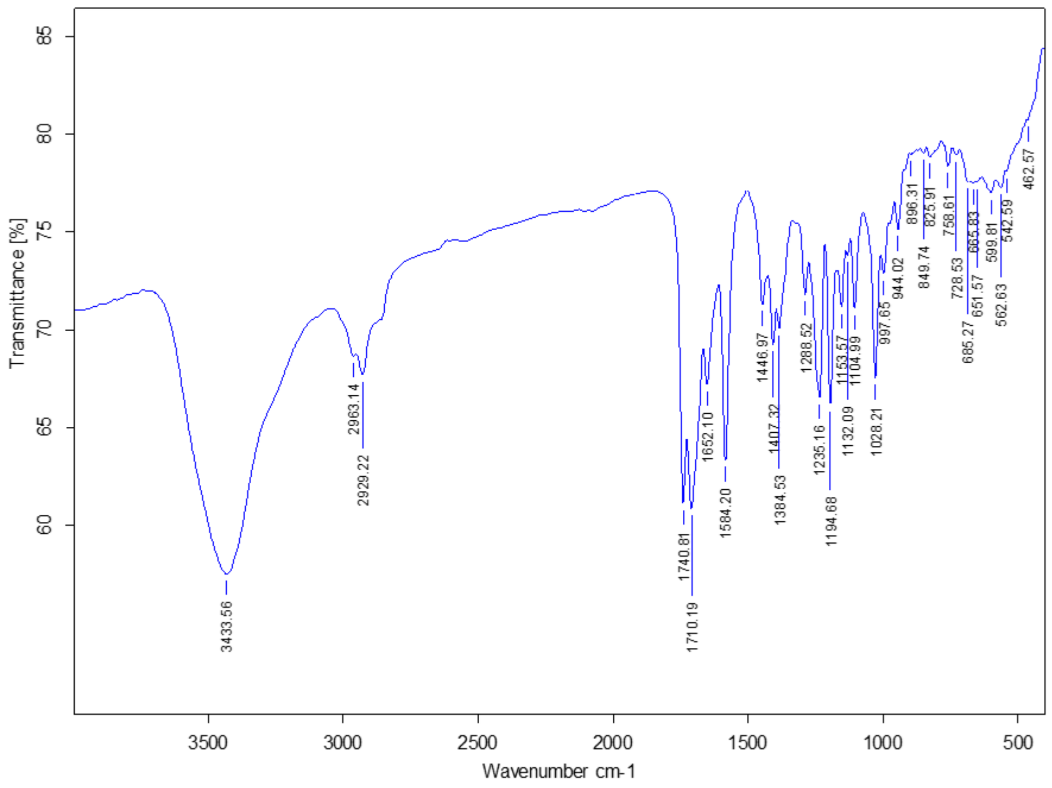

Figure S23. IR spectrum of asperversin B (**2**).

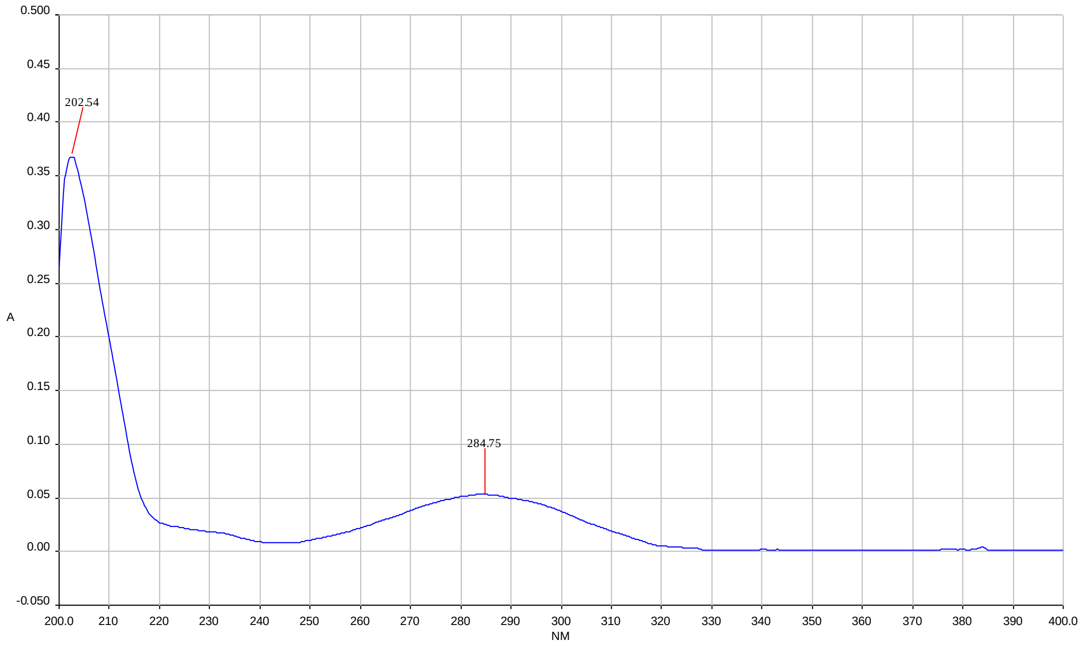

Figure S24. UV spectrum of asperversin B (**2**).

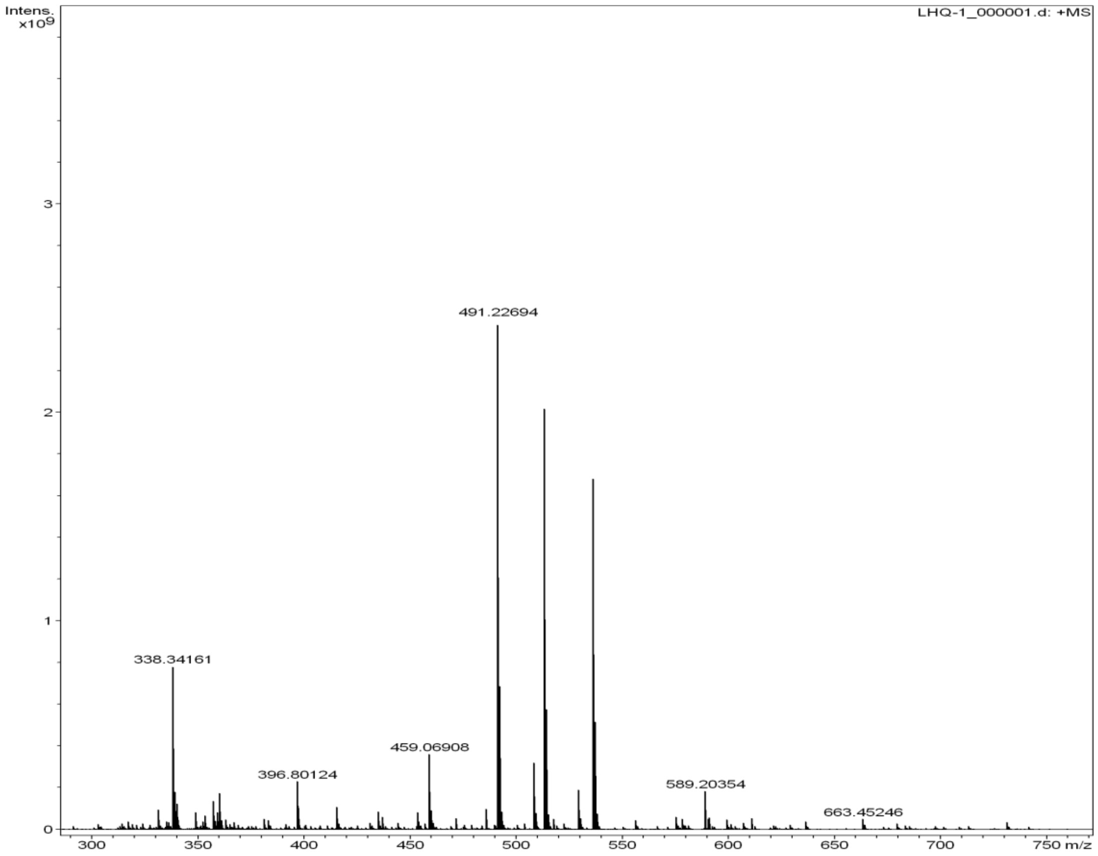

Figure S25. HRESIMS spectrum of asperversin C (3).

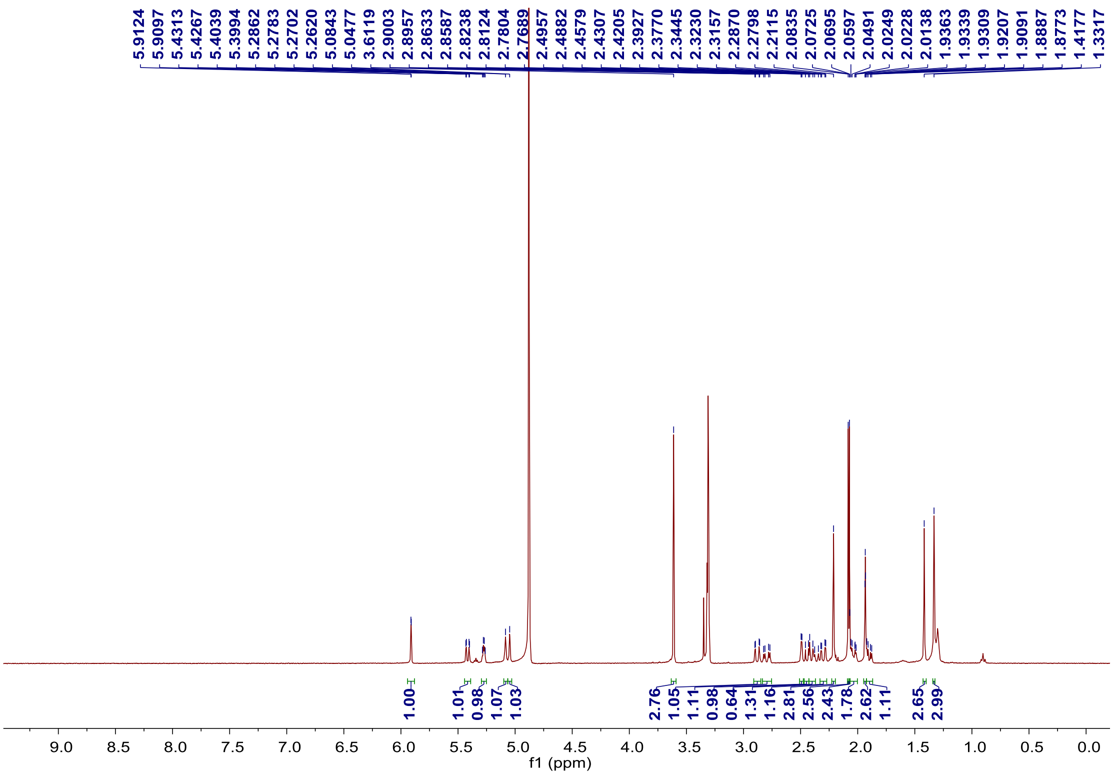

Figure S26. ^{1}H NMR spectrum of asperversin C (**3**) (400 MHz, methanol-d_4).

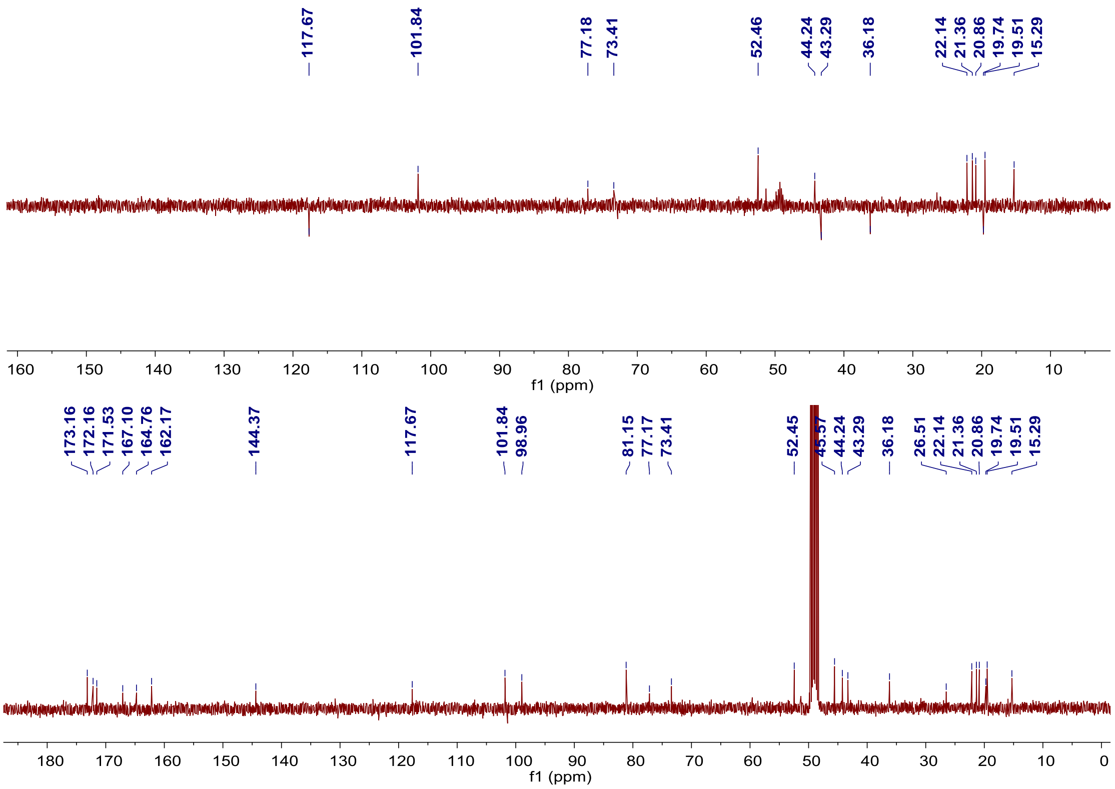

Figure S27. ^{13}C NMR and DEPT spectra of asperversin C (**3**) (100 MHz, methanol-d_4).

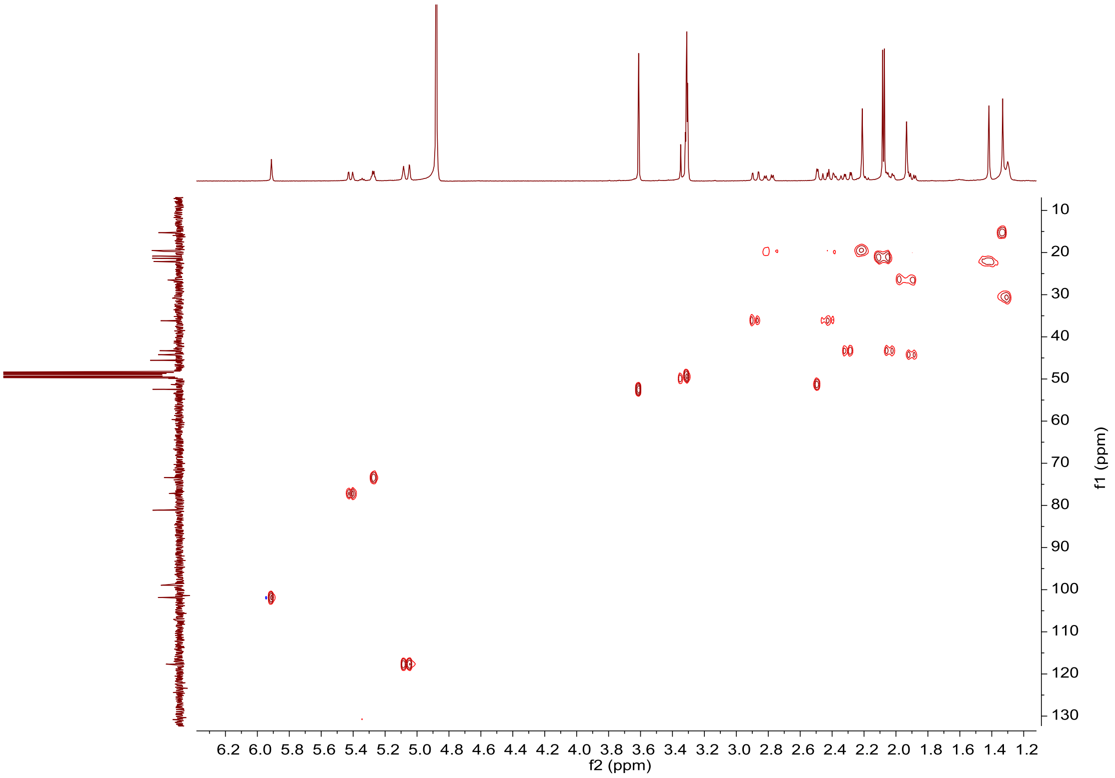

Figure S28. HSQC spectrum of asperversin C (**3**) (methanol-d_4).

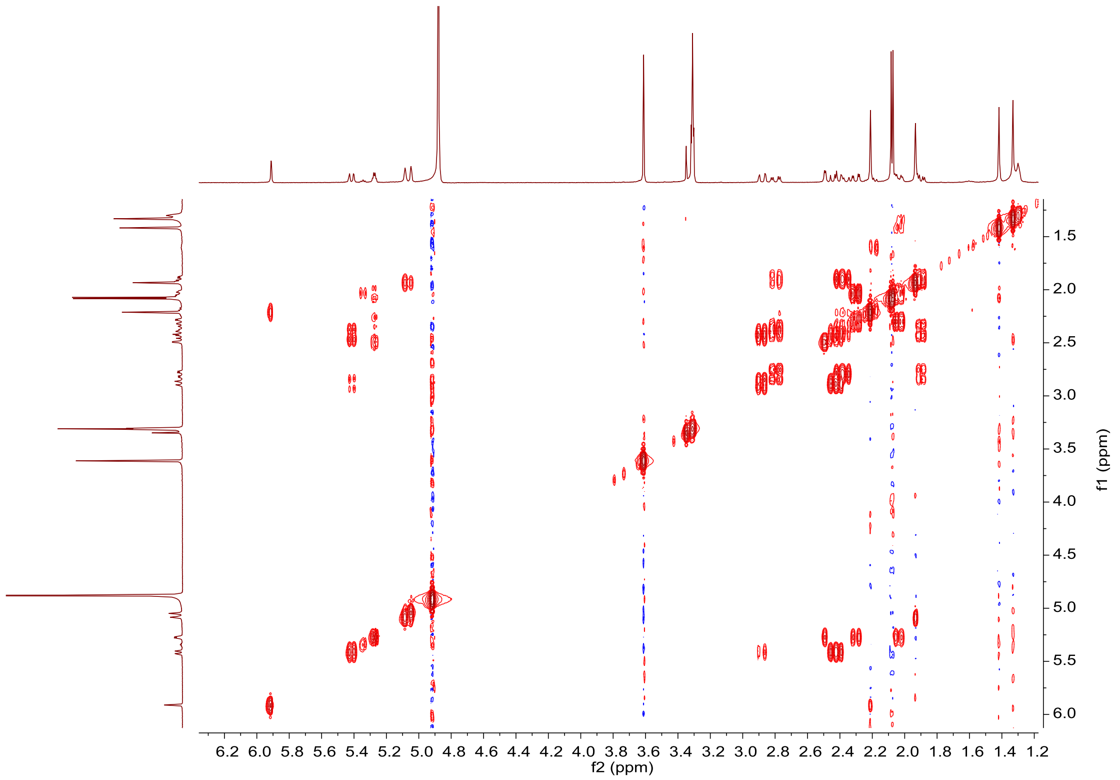

Figure S29. $^{1}H-^{1}H$ COSY spectrum of asperversin C (**3**) (methanol-d_4).

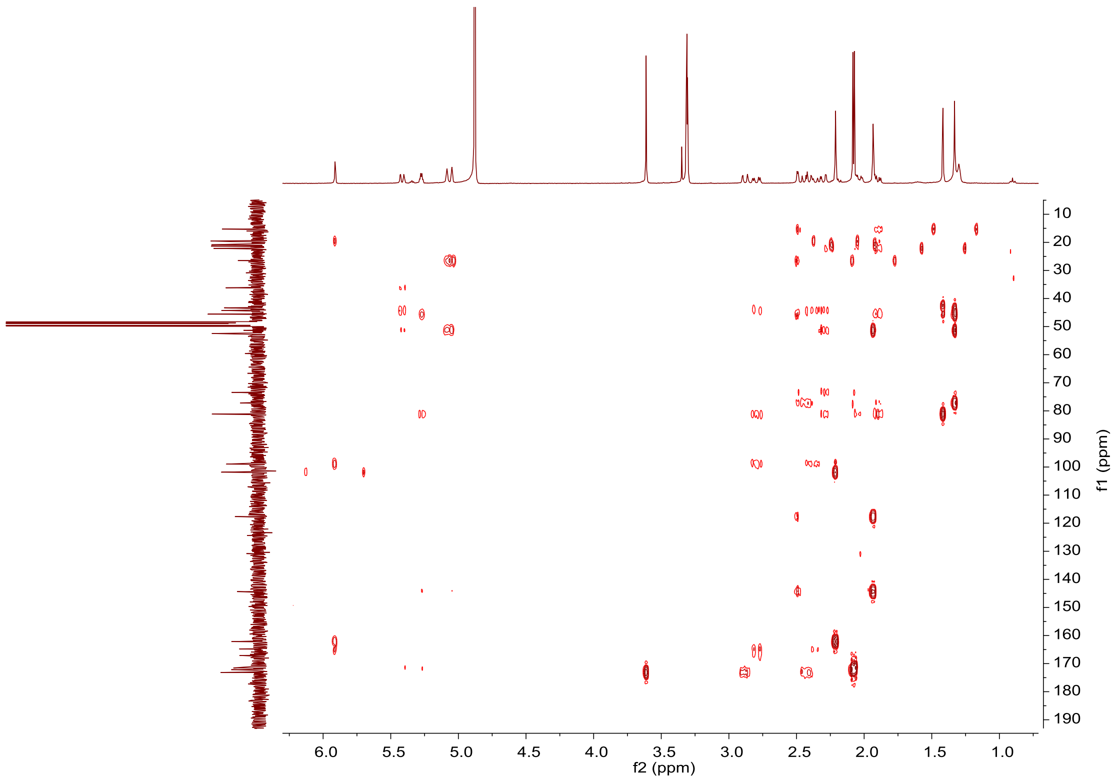

Figure S30. HMBC spectrum of asperversin C (**3**) (methanol-d_4).

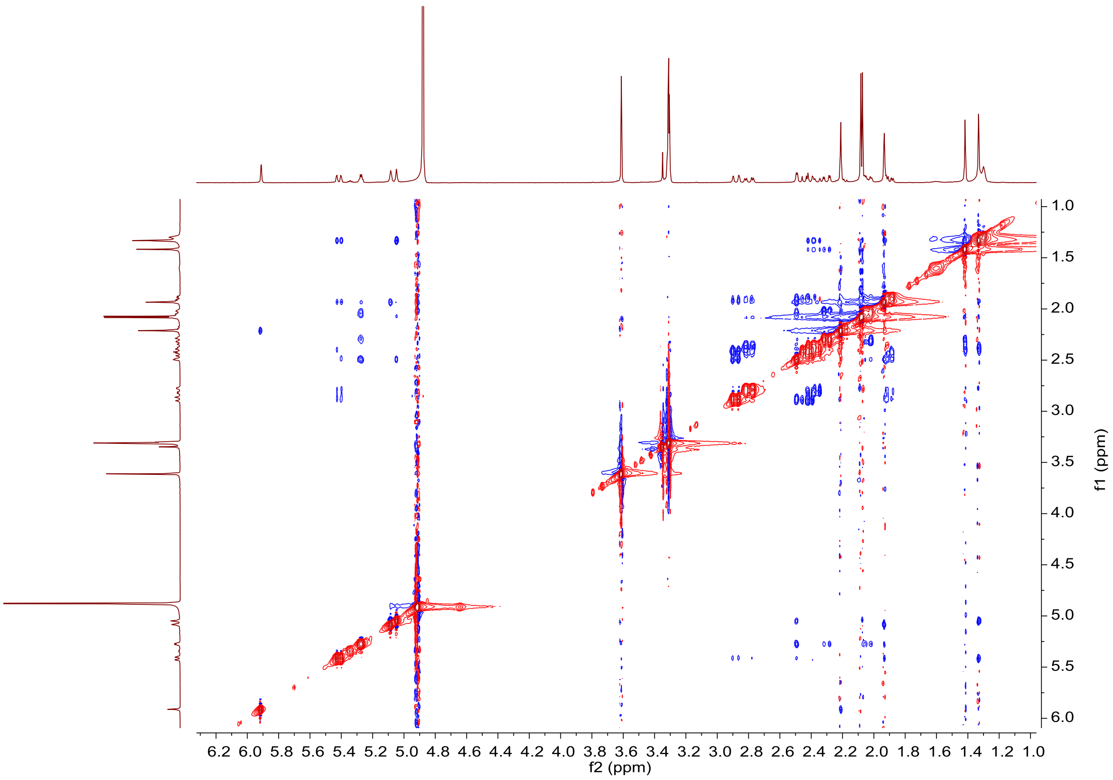

Figure S31. NOESY spectrum of asperversin C (**3**) (methanol-*d*4).

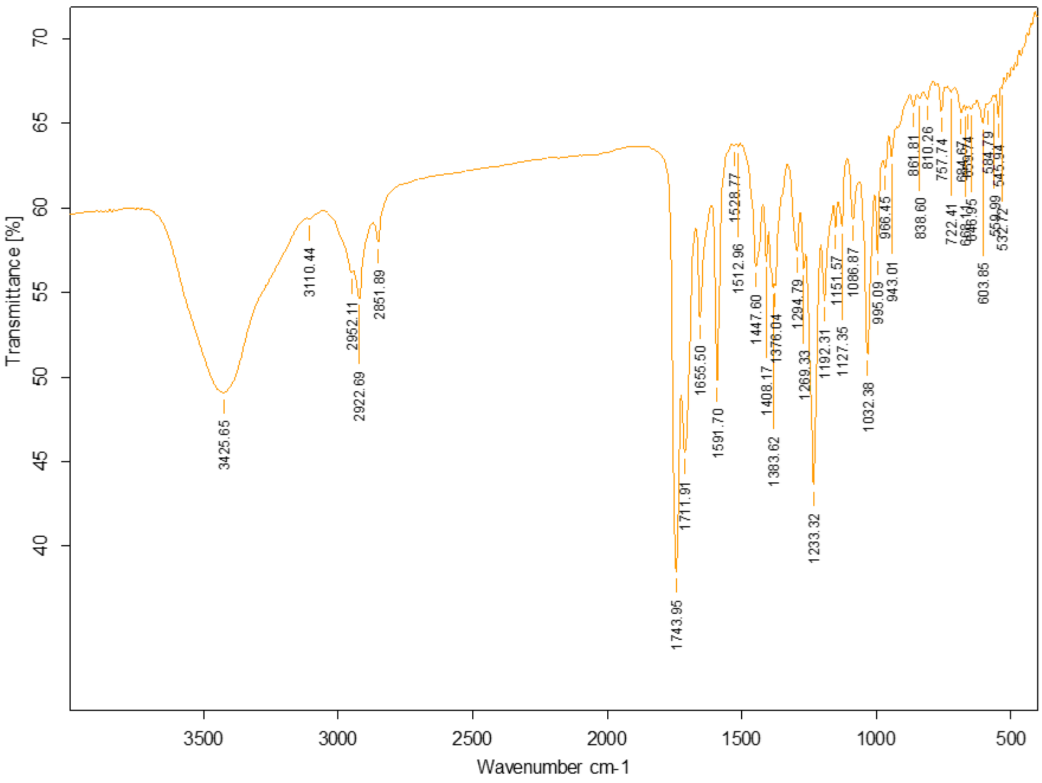

Figure S32. IR spectrum of asperversin C (**3**).

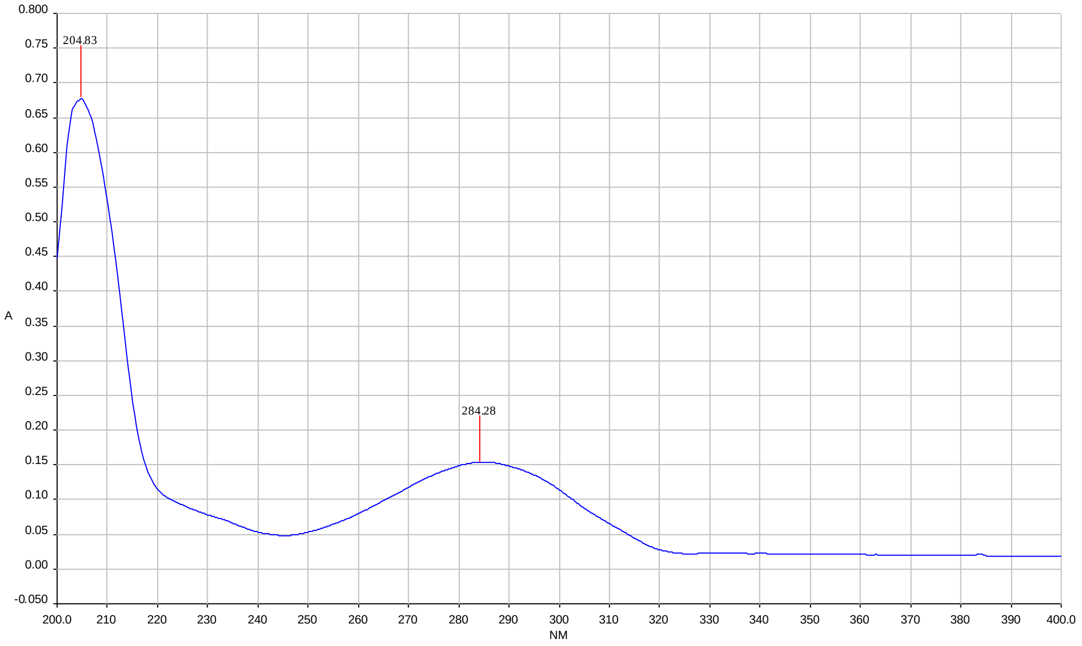

Figure S33. UV spectrum of asperversin C (**3**).

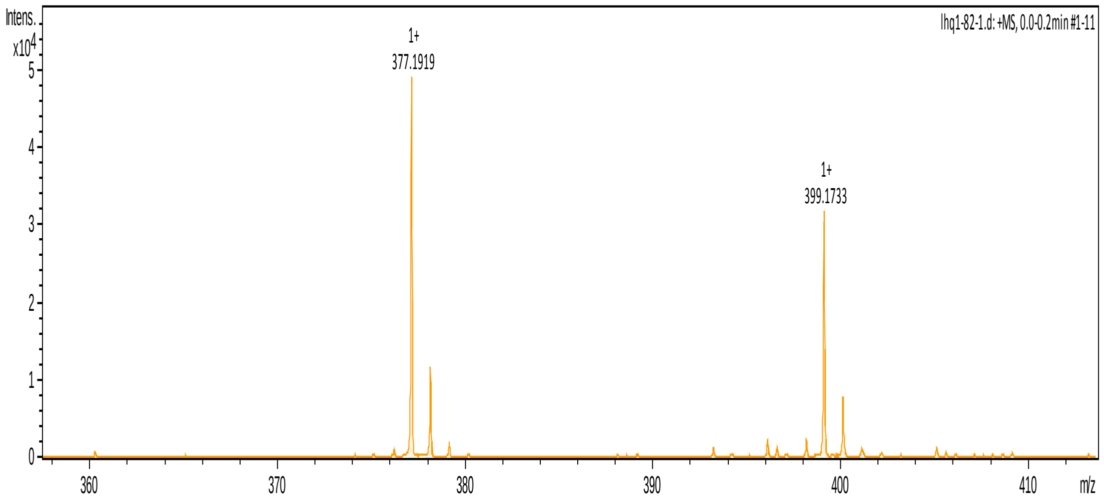

Figure S34. HRESIMS spectrum of asperversin D (**4**).

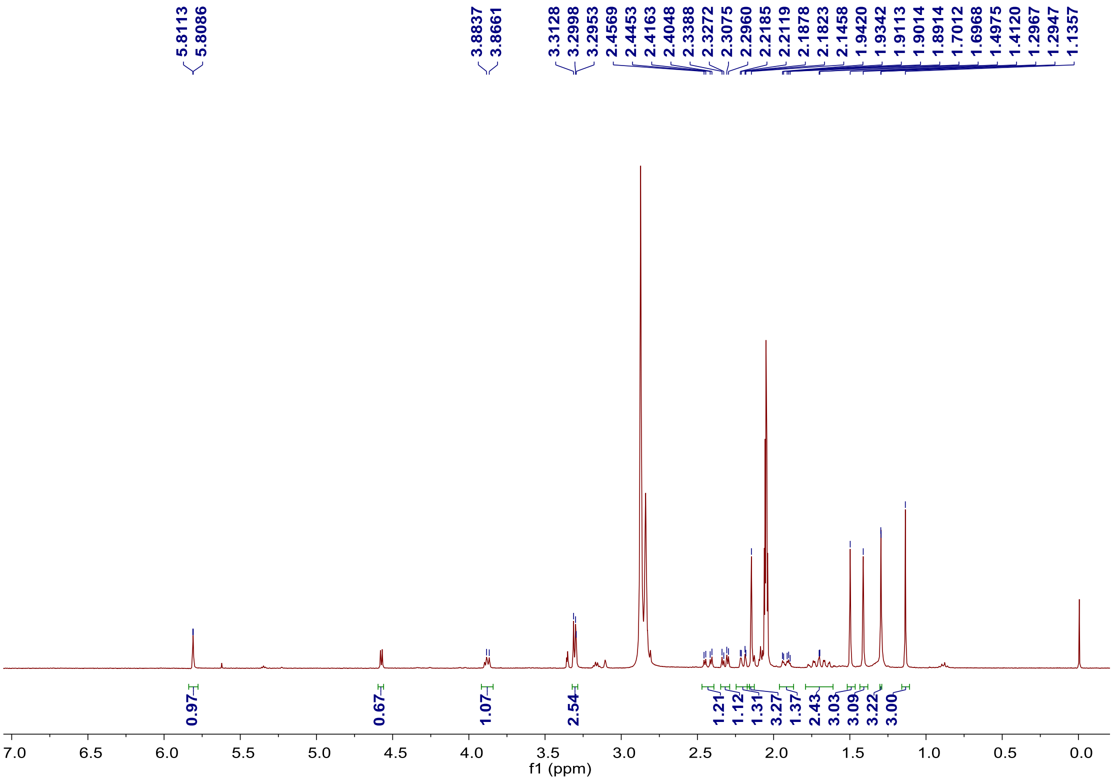

Figure S35. ^{1}H NMR spectrum of asperversin D (**4**) (400 MHz, acetone-d_6).

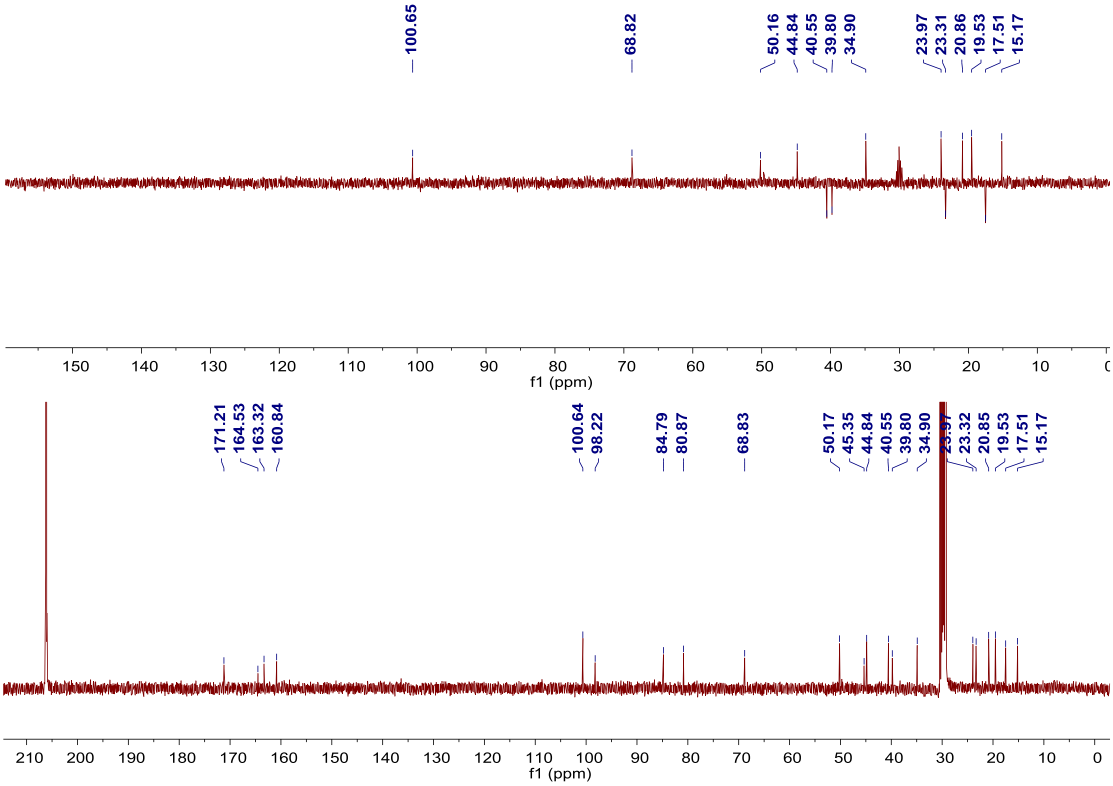

Figure S36. ^{13}C NMR and DEPT spectra of asperversin D (**4**) (100 MHz, acetone-d_6).

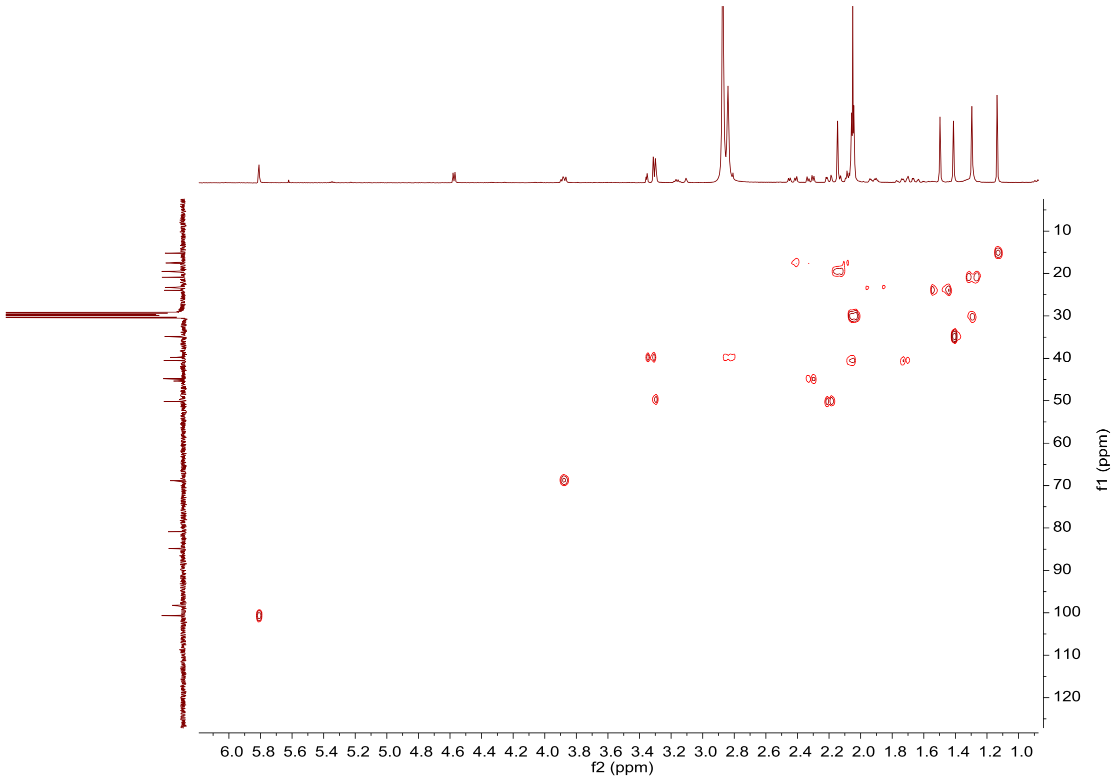

Figure S37. HSQC spectrum of asperversin D (**4**) (acetone-d_6).

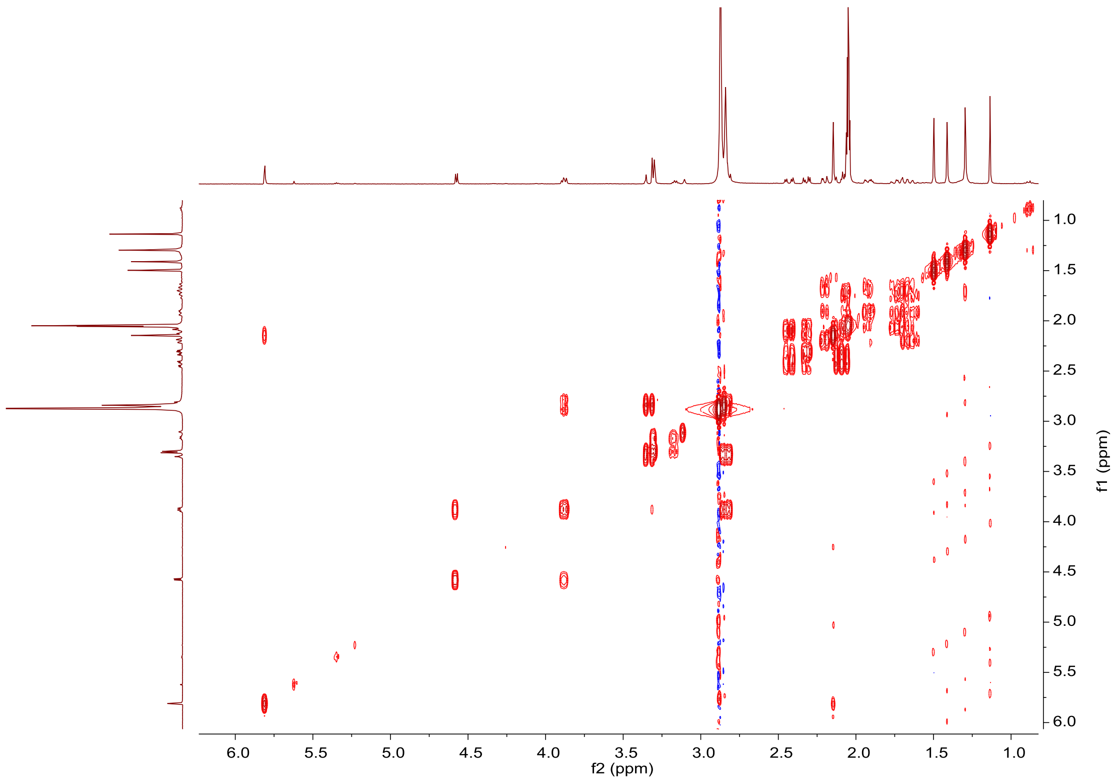

Figure S38. ^{1}H–^{1}H COSY spectrum of asperversin D (**4**) (acetone-d_6).

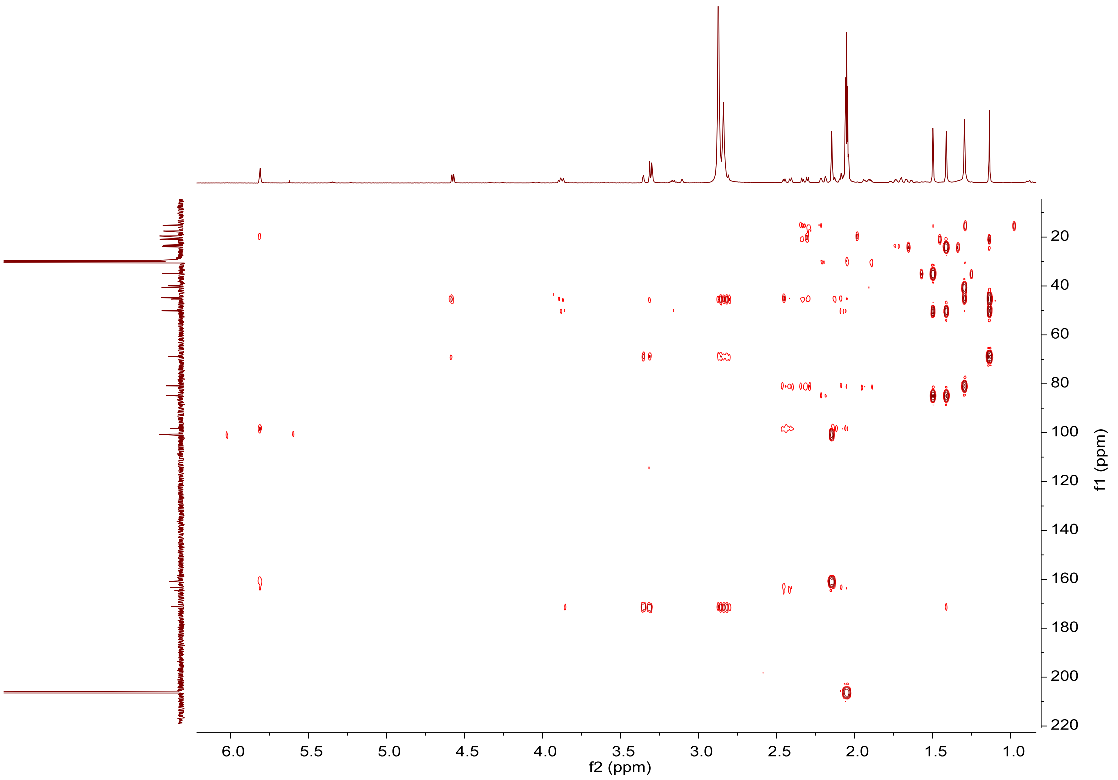

Figure S39. HMBC spectrum of asperversin D (**4**) (acetone-d_6).

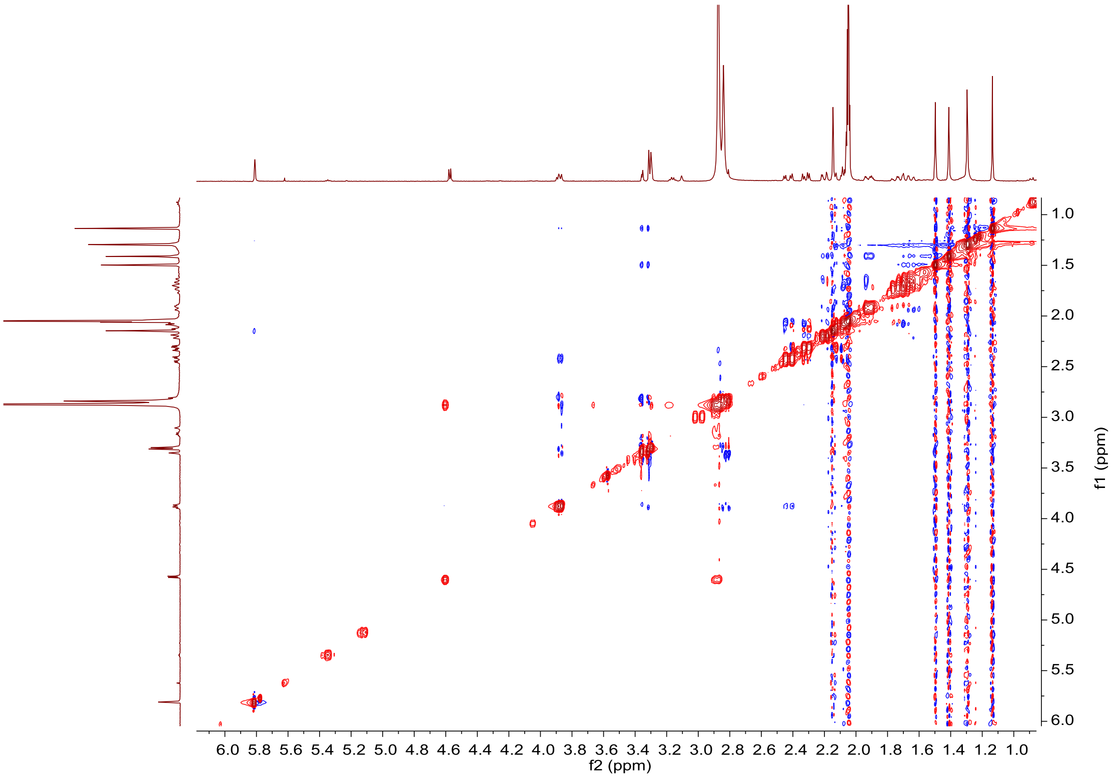

Figure S40. NOESY spectrum of asperversin D (**4**) (acetone-d_6).

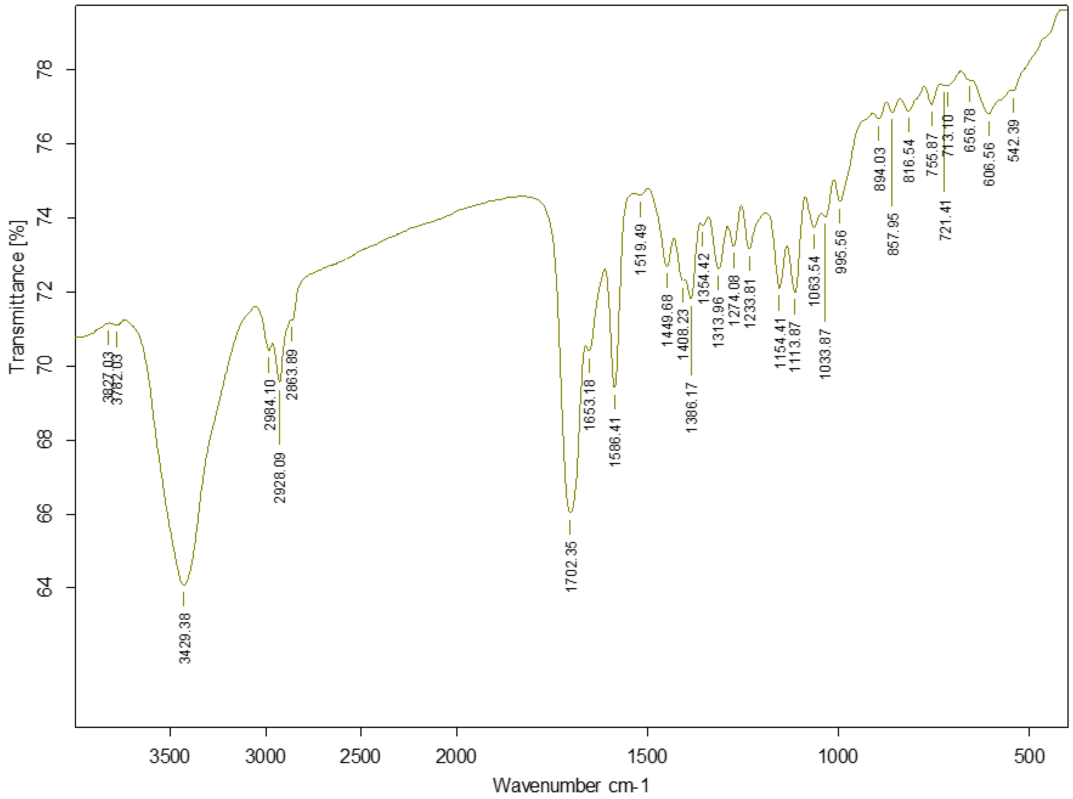

Figure S41. IR spectrum of asperversin D (**4**).

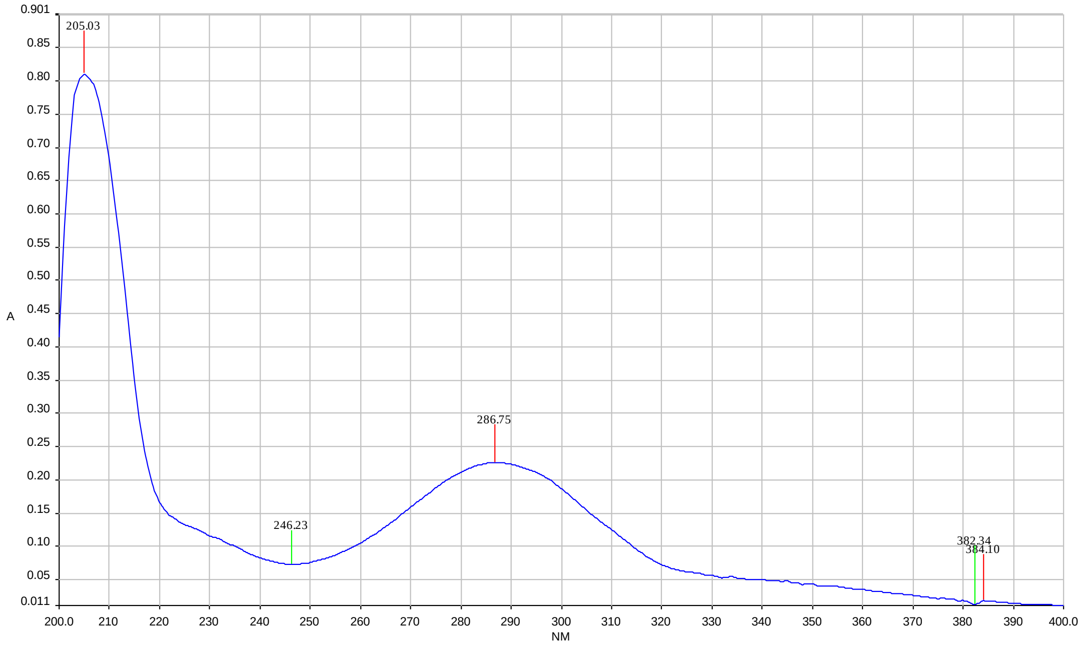

Figure S42. UV spectrum of asperversin D (**4**).

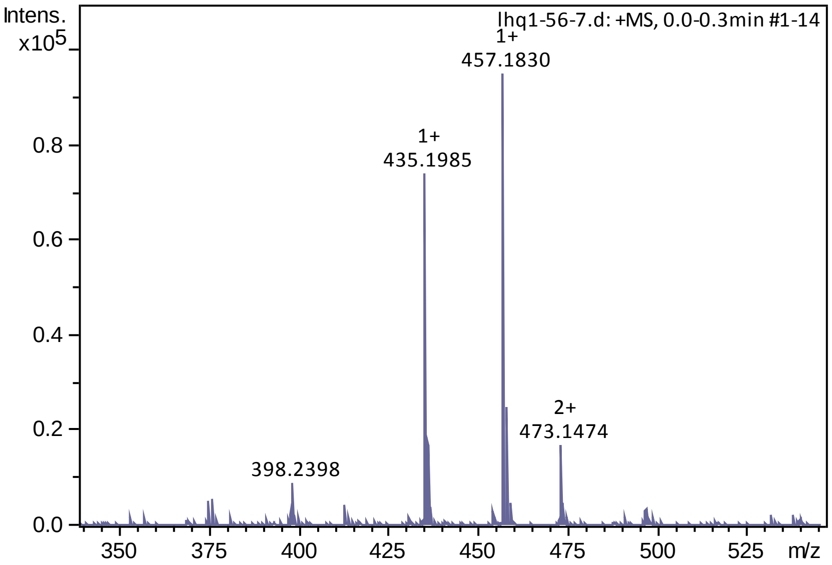

Figure S43. HRESIMS spectrum of asperversin E (**5**).

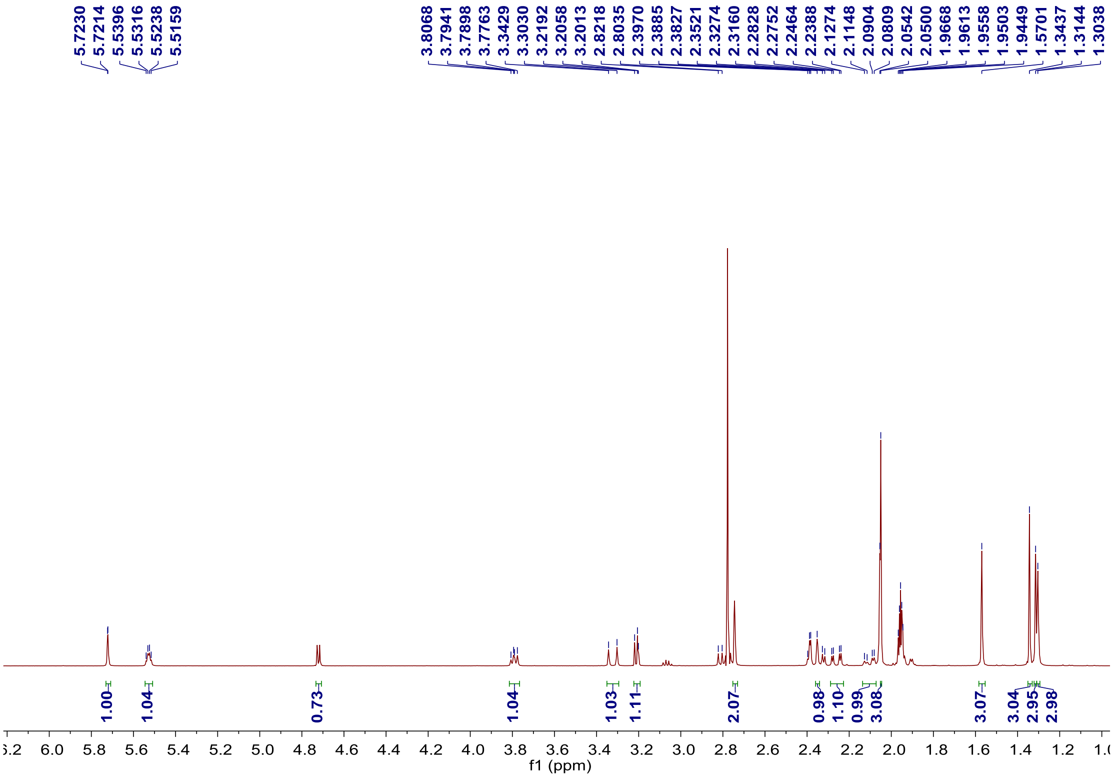

Figure S44. ^{1}H NMR spectrum of asperversin E (**5**) (400 MHz, acetone-d_6).

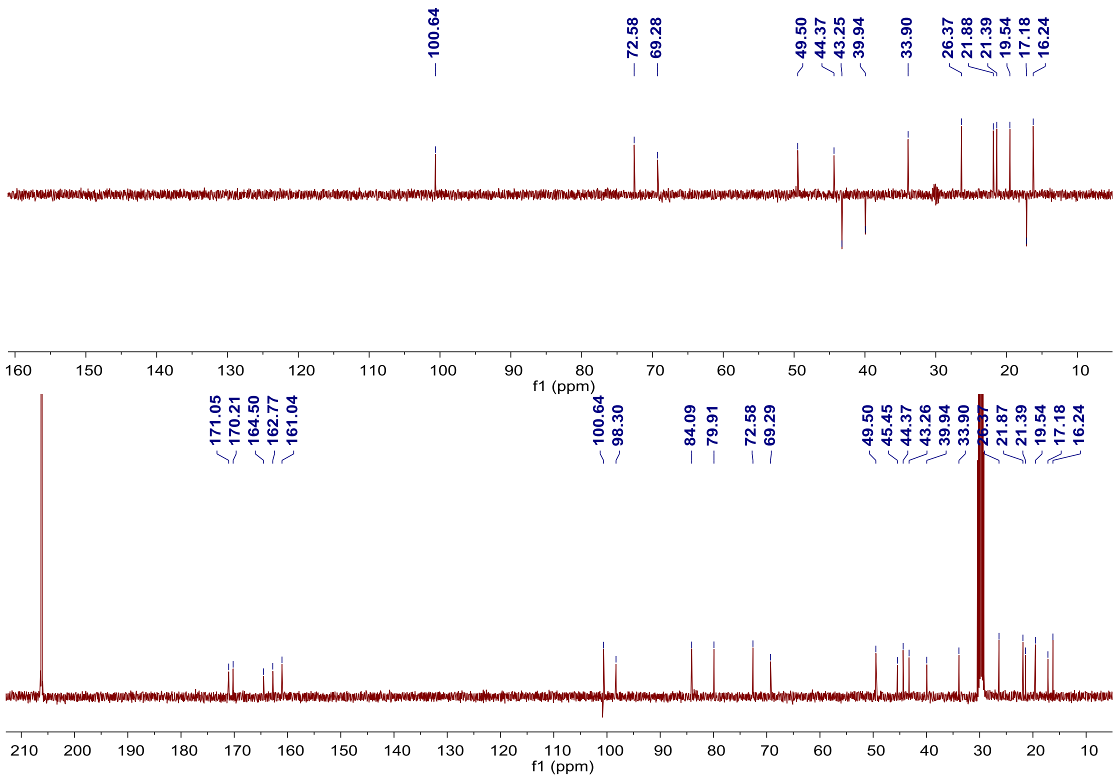

Figure S45. ^{13}C NMR and DEPT spectra of asperversin E (**5**) (100 MHz, acetone-d_6).

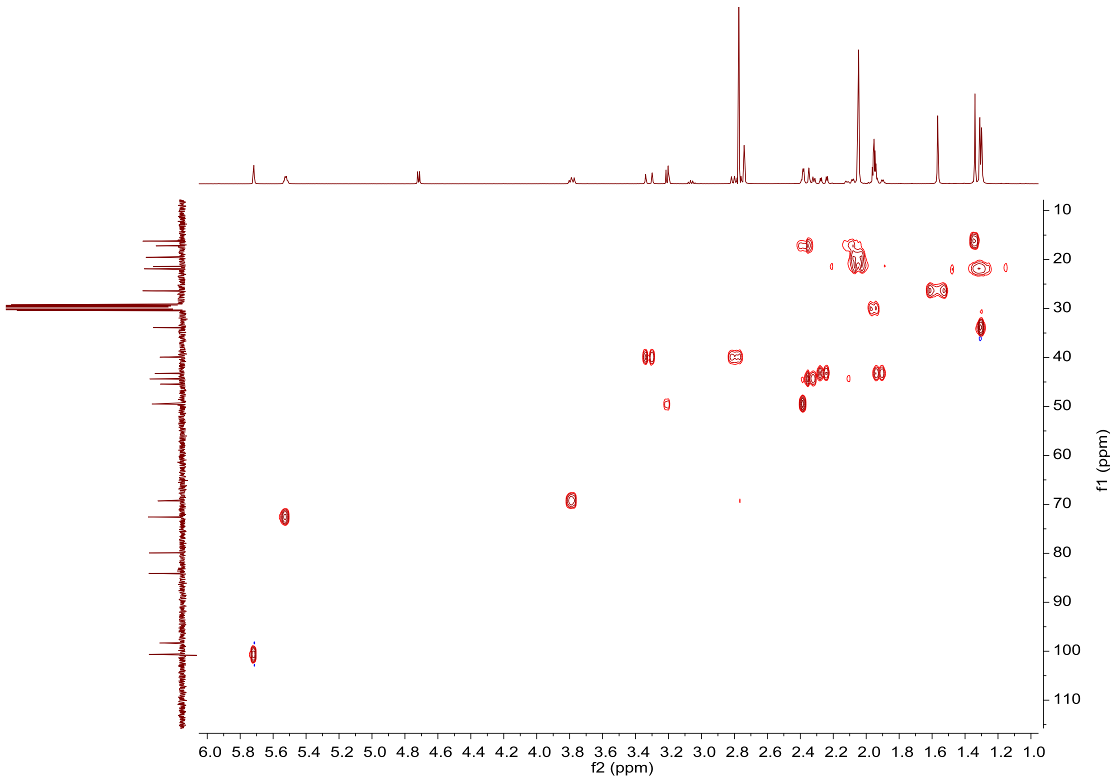

Figure S46. HSQC spectrum of asperversin E (**5**) (acetone-d_6).

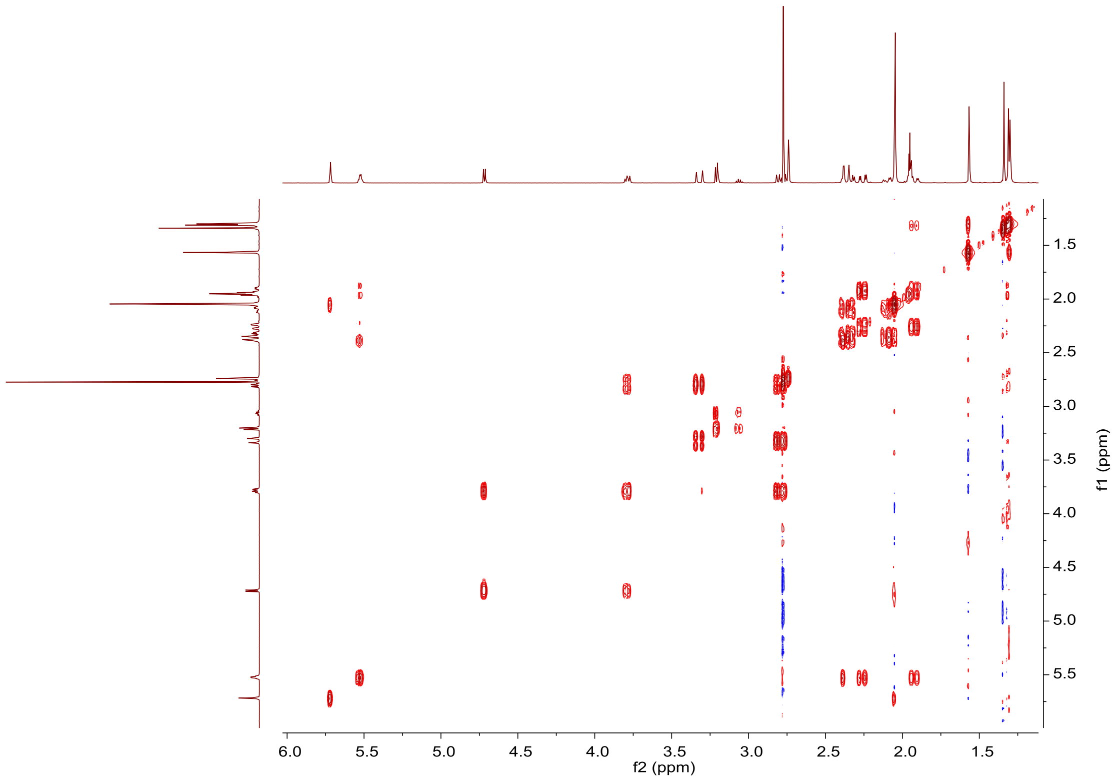

Figure S47. ^{1}H–^{1}H COSY spectrum of asperversin E (**5**) (acetone-d_6).

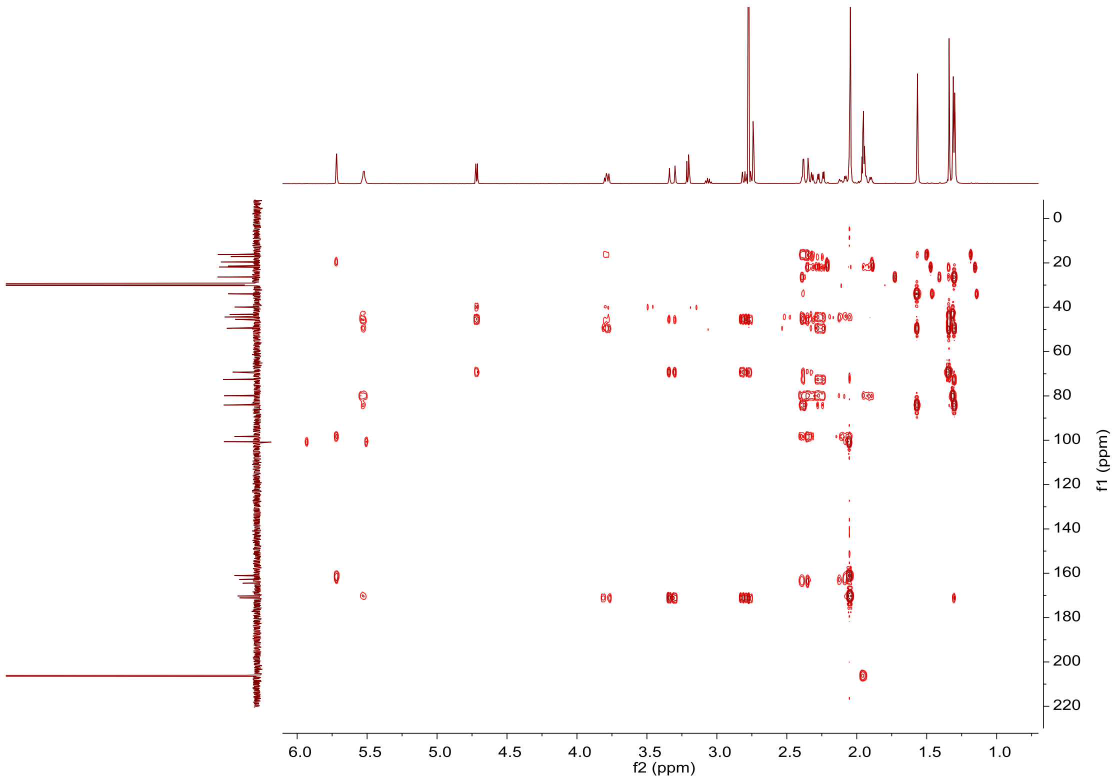

Figure S48. HMBC spectrum of asperversin E (**5**) (acetone-d_6).

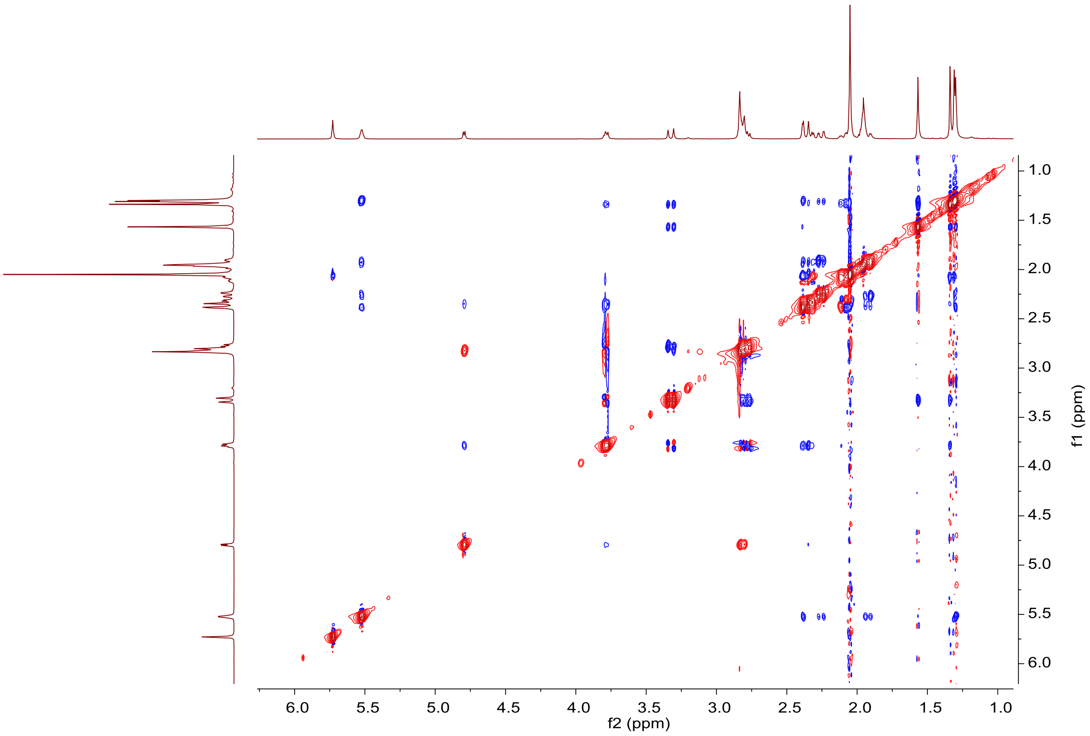

Figure S49. NOESY spectrum of asperversin E (**5**) (acetone-d_6).

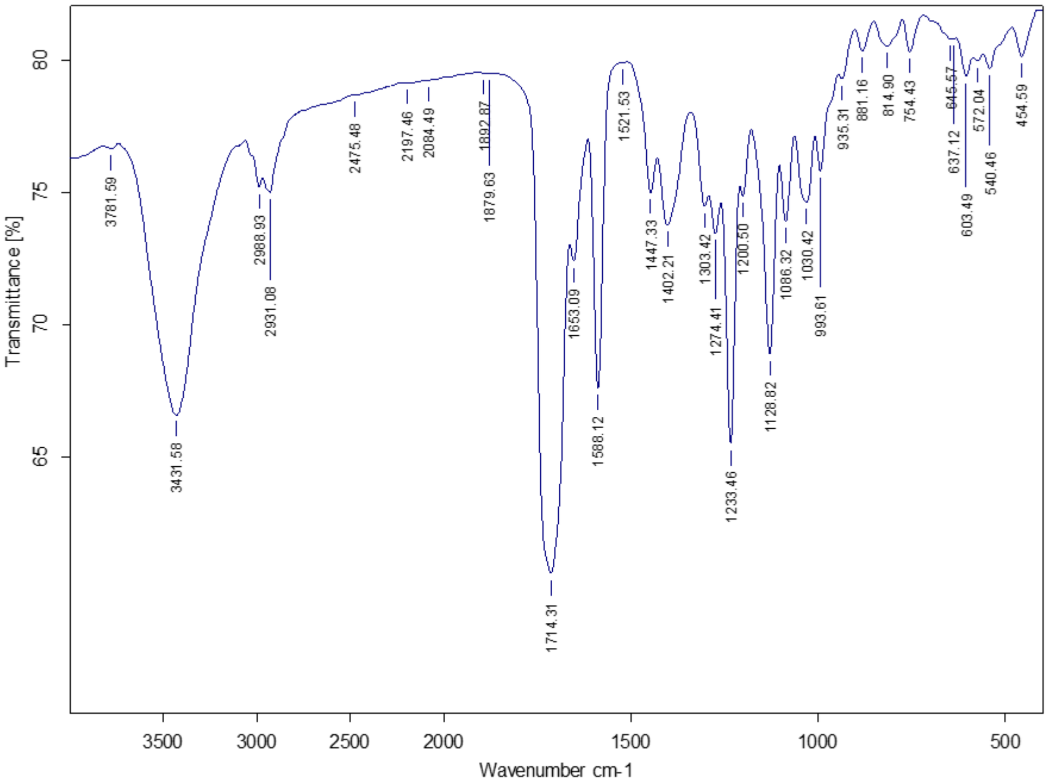

Figure S50. IR spectrum of asperversin E (**5**).

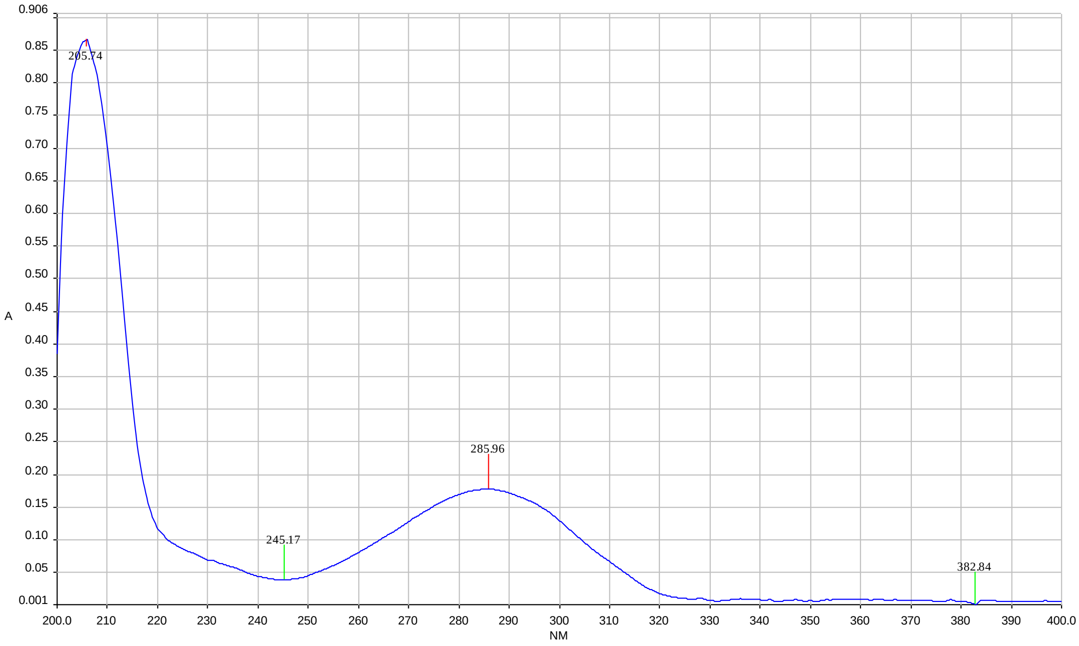

Figure S51. UV spectrum of asperversin E (**5**).

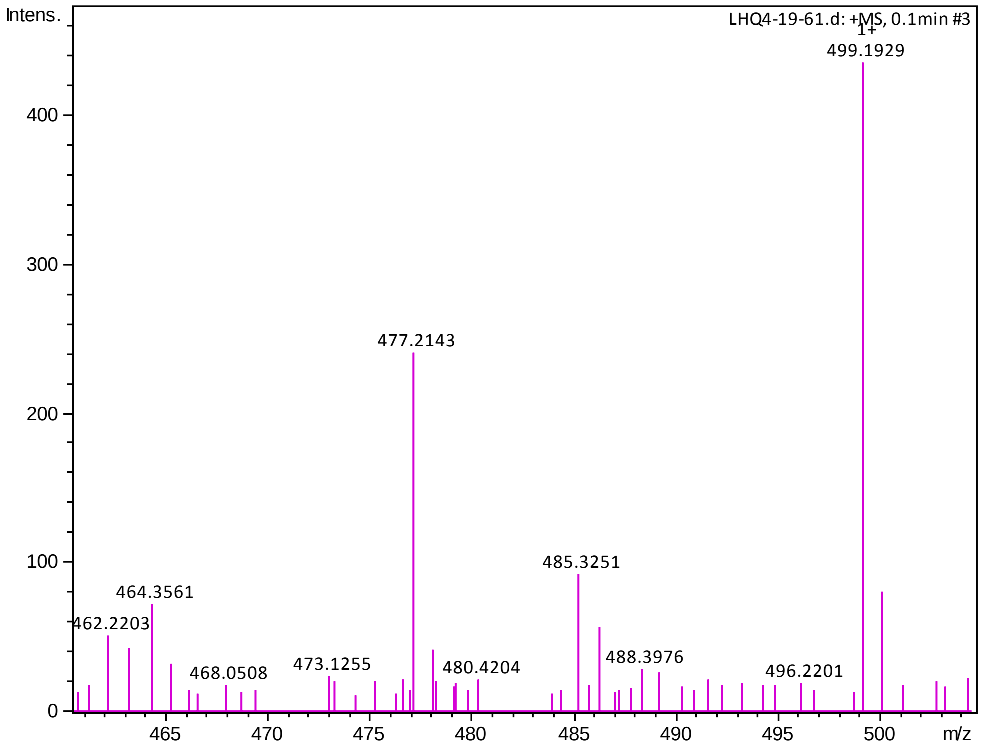

Figure S52. HRESIMS spectrum of asperversin F (**6**).

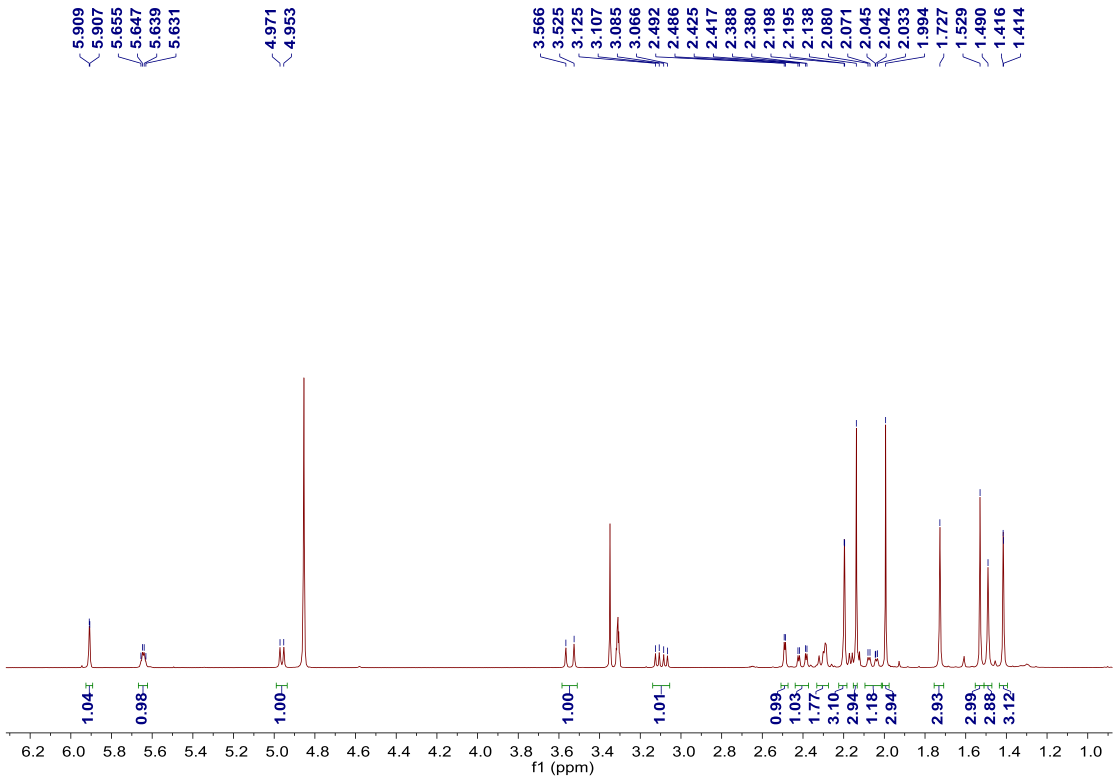

Figure S53. ^{1}H NMR spectrum of asperversin F (**6**) (400 MHz, methanol-d_4).

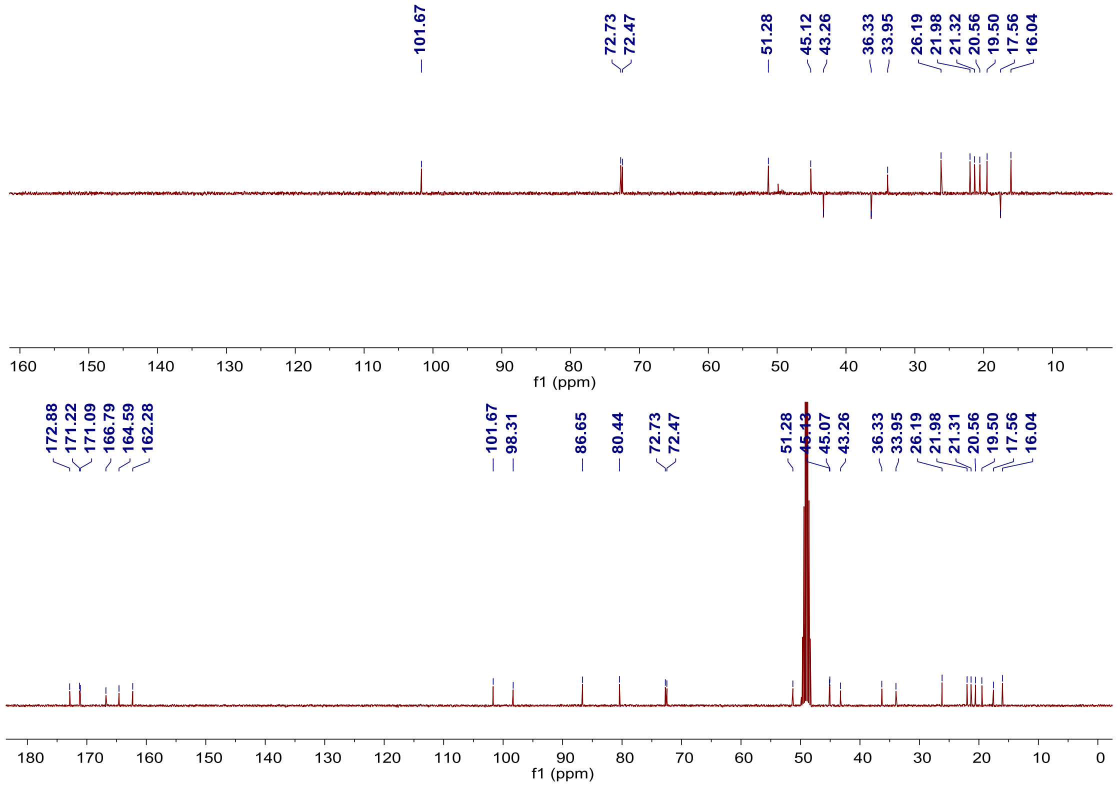

Figure S54. ^{13}C NMR and DEPT spectra of asperversin F (**6**) (100 MHz, methanol-d_4).

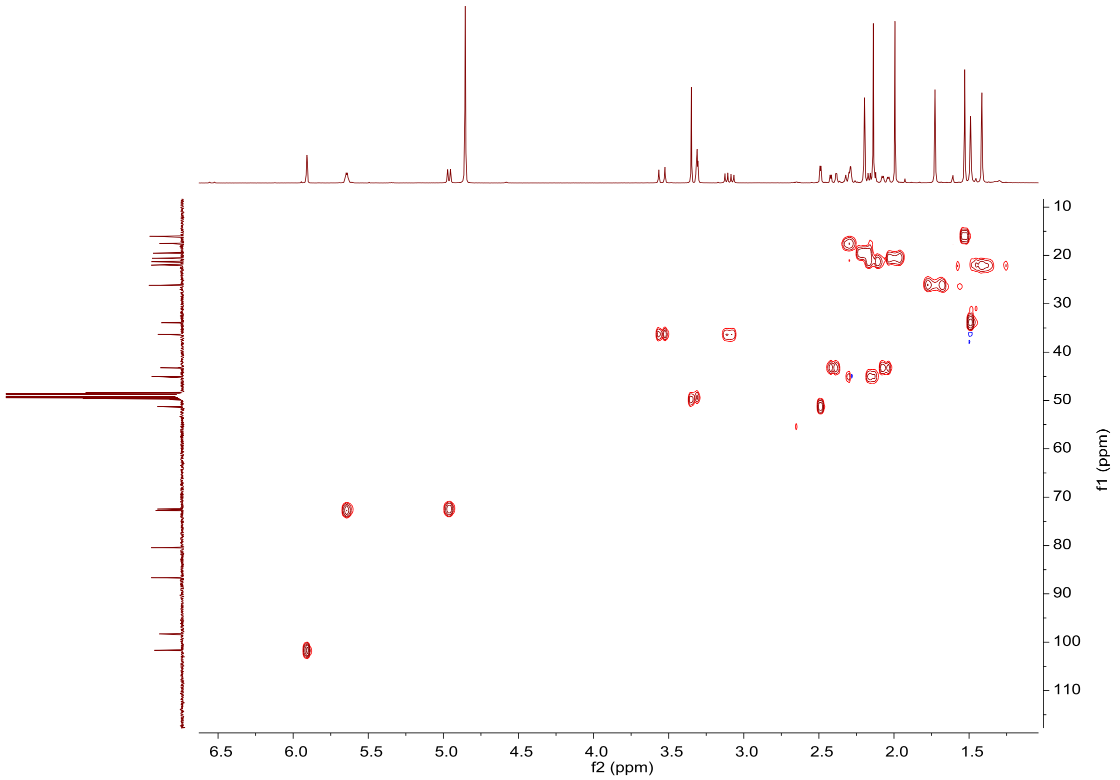

Figure S55. HSQC spectrum of asperversin F (**6**) (methanol-*d*4).

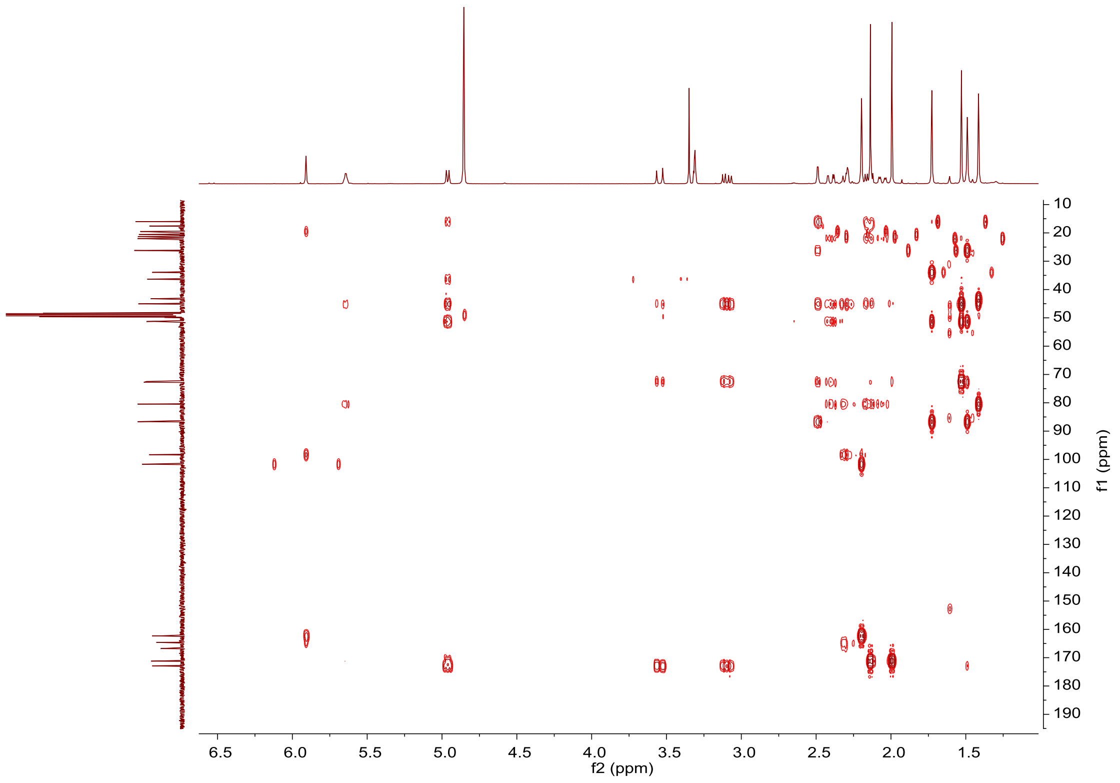

Figure S56. ^{1}H–^{1}H COSY spectrum of asperversin F (**6**) (methanol-*d*4).

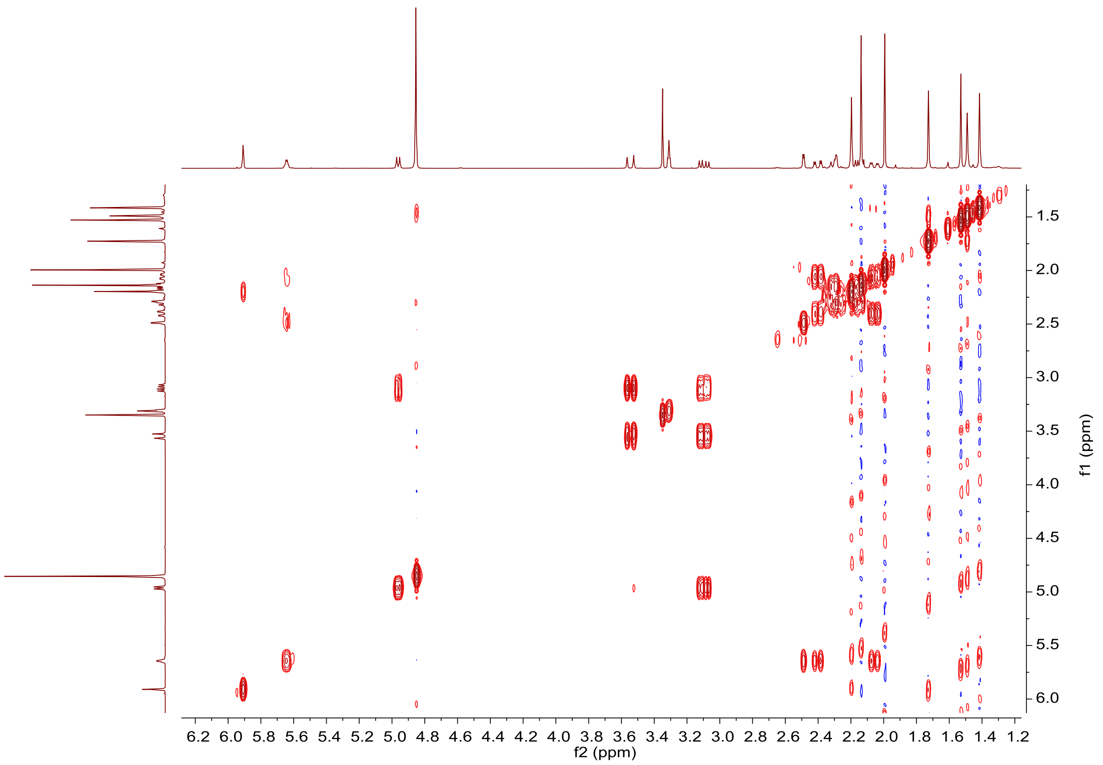

Figure S57. HMBC spectrum of asperversin F (**6**) (methanol-*d*4).

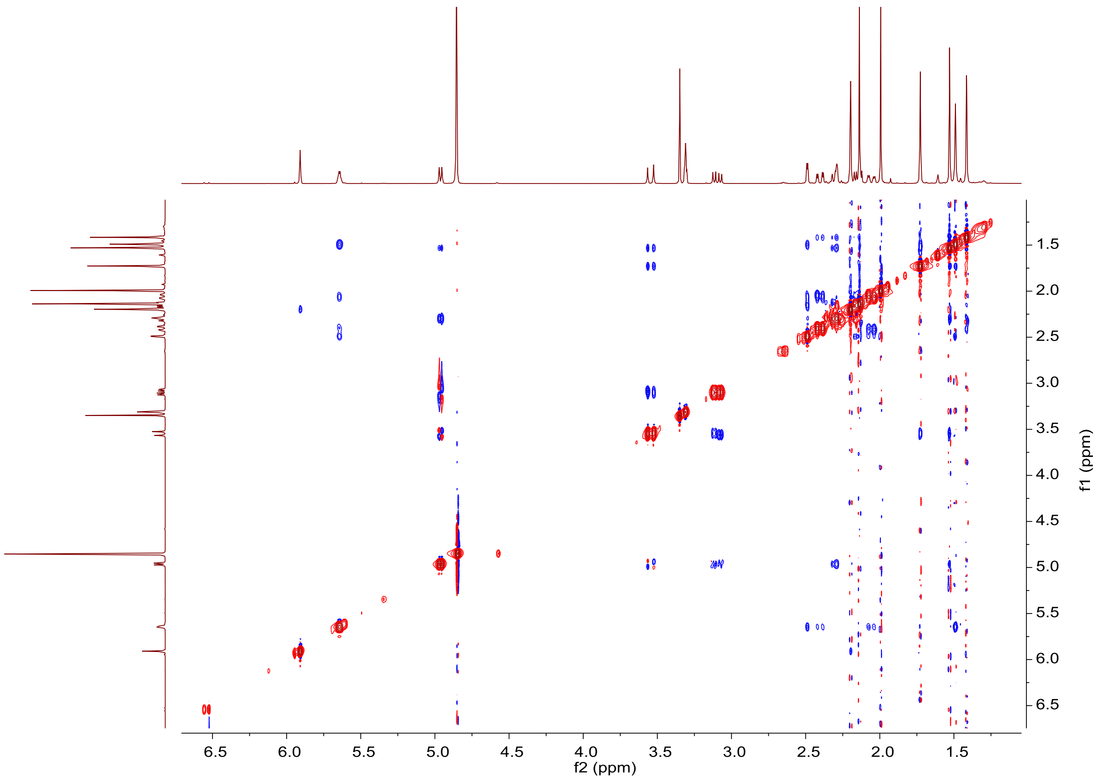

Figure S58. NOESY spectrum of asperversin F (**6**) (methanol-*d*4).

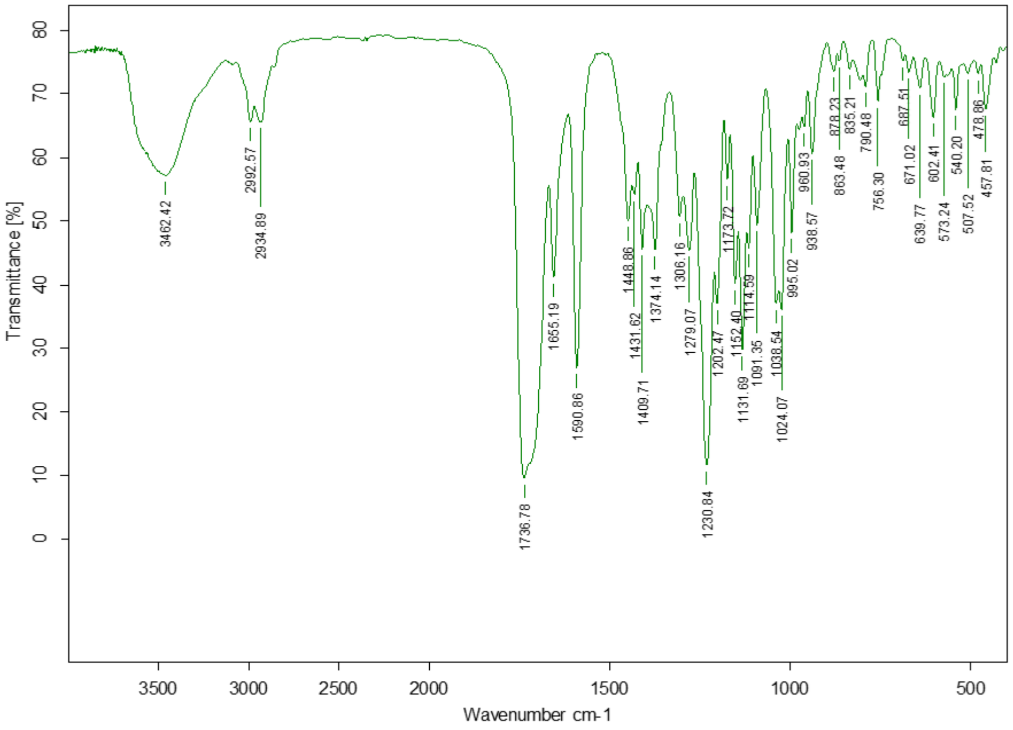

Figure S59. IR spectrum of asperversin F (**6**).

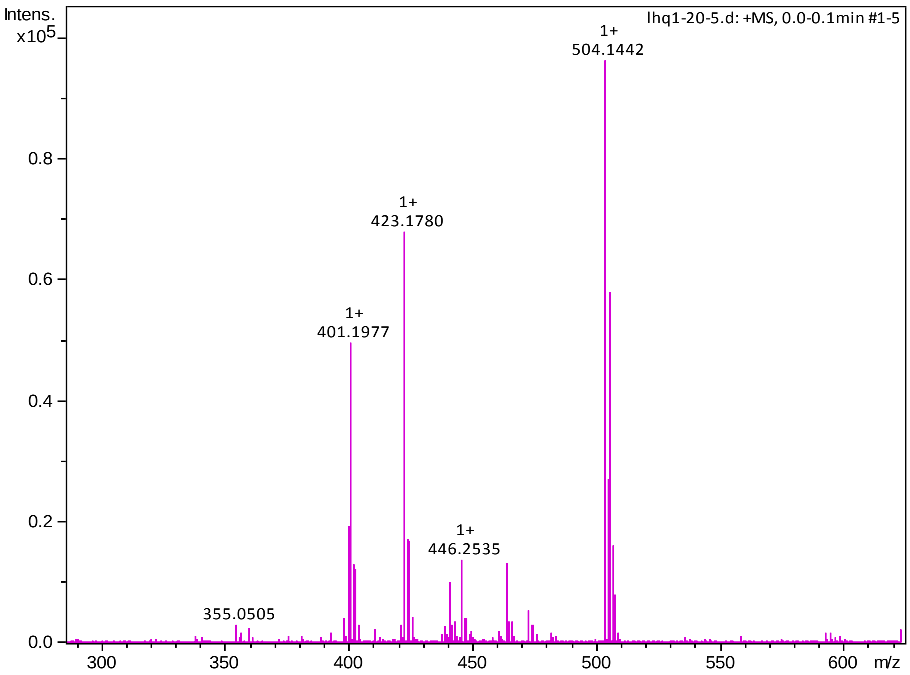

Figure S60. HRESIMS spectrum of asperversin G (**7**).

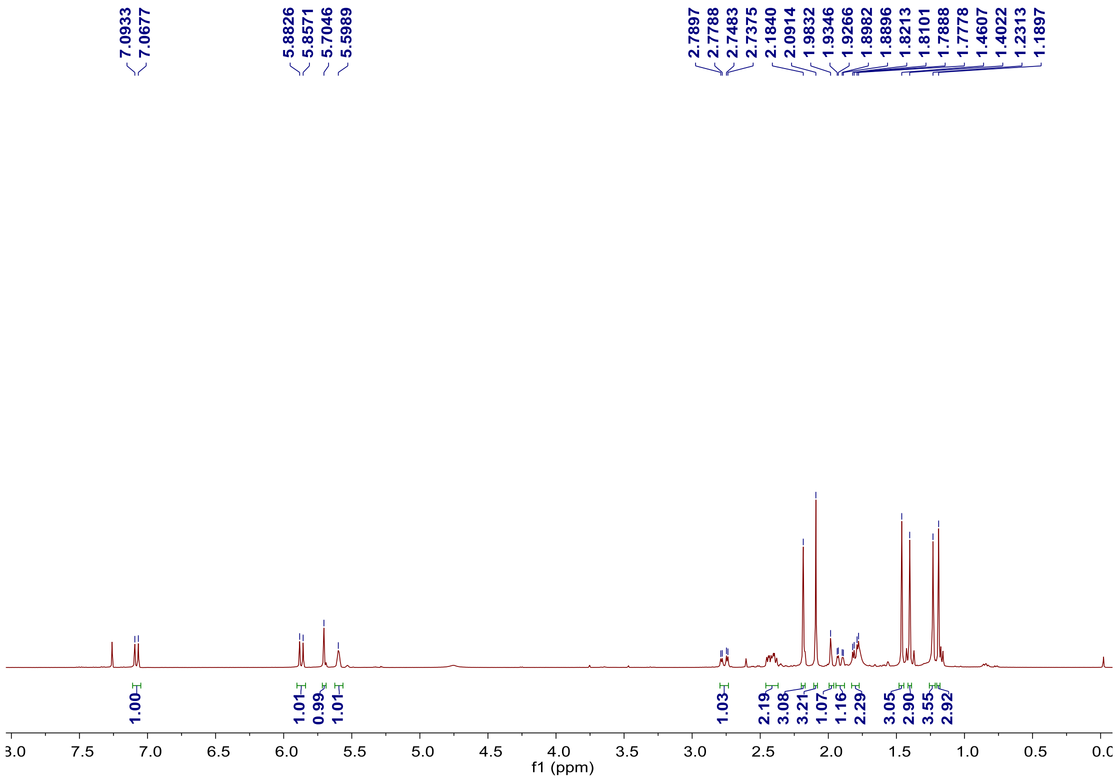

Figure S61. ^{1}H NMR spectrum of asperversin G (**7**) (400 MHz, $CDCl_3$).

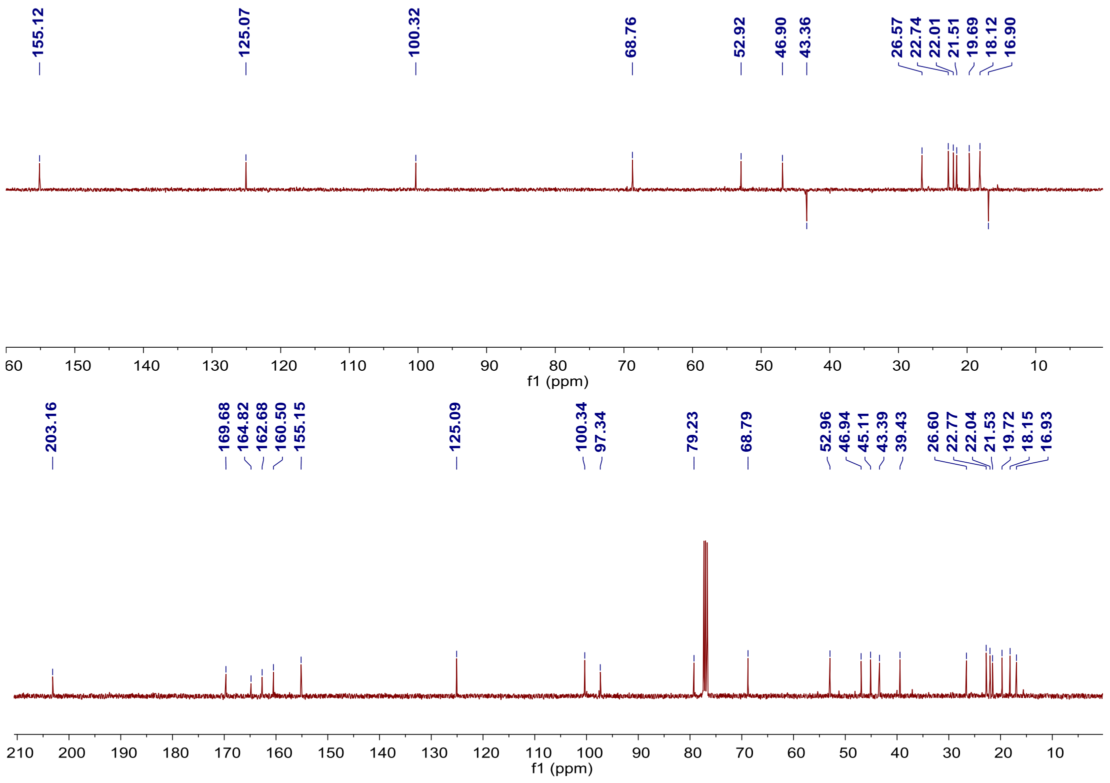

Figure S62. ^{13}C NMR and DEPT spectra of asperversin G (**7**) (100 MHz, $CDCl_3$).

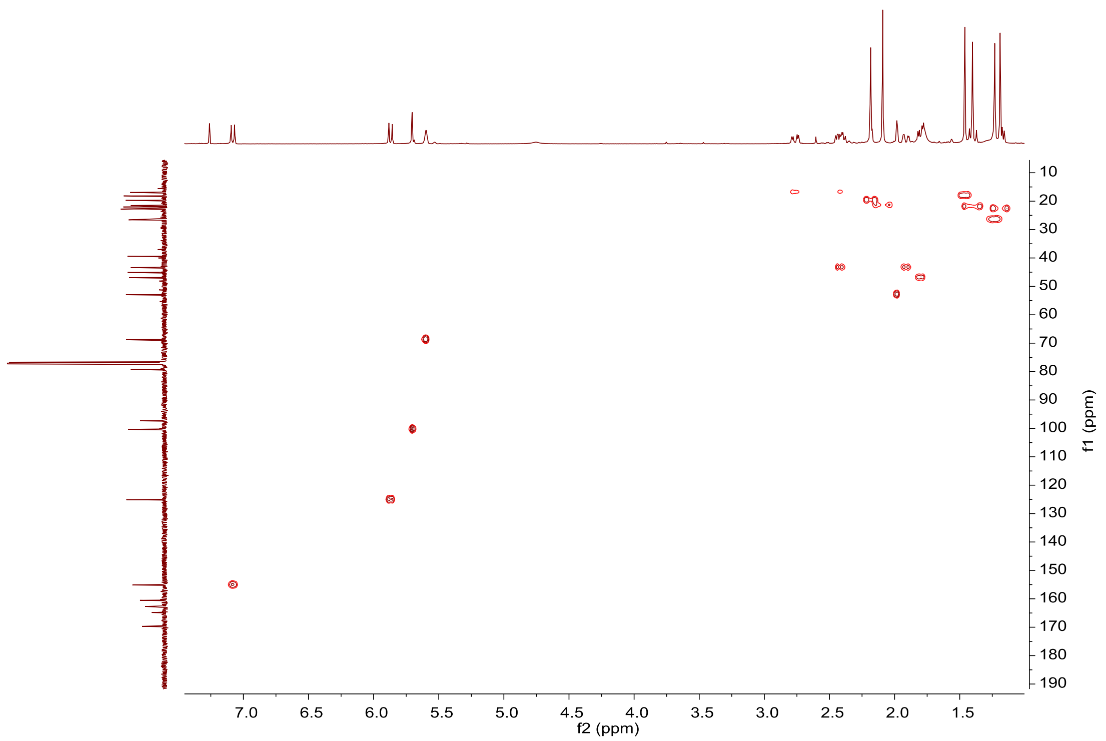

Figure S63. HSQC spectrum of asperversin G (**7**) ($CDCl_3$).

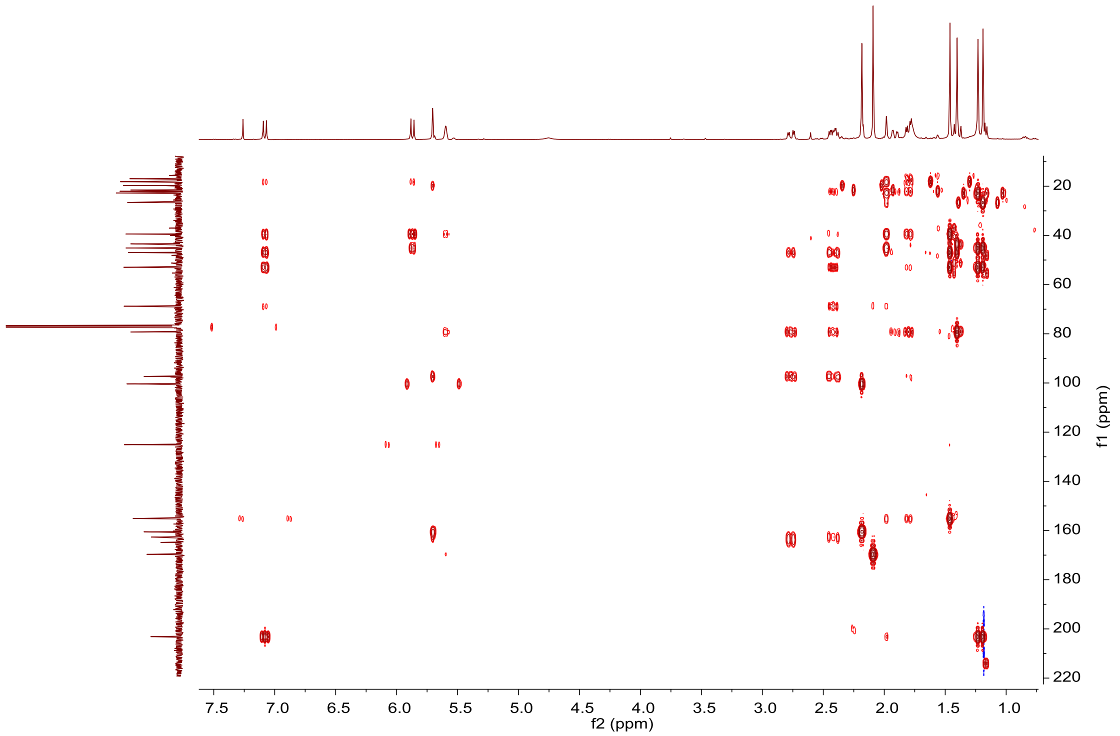

Figure S64. ^1H–^1H COSY spectrum of asperversin G (**7**) ($CDCl_3$).

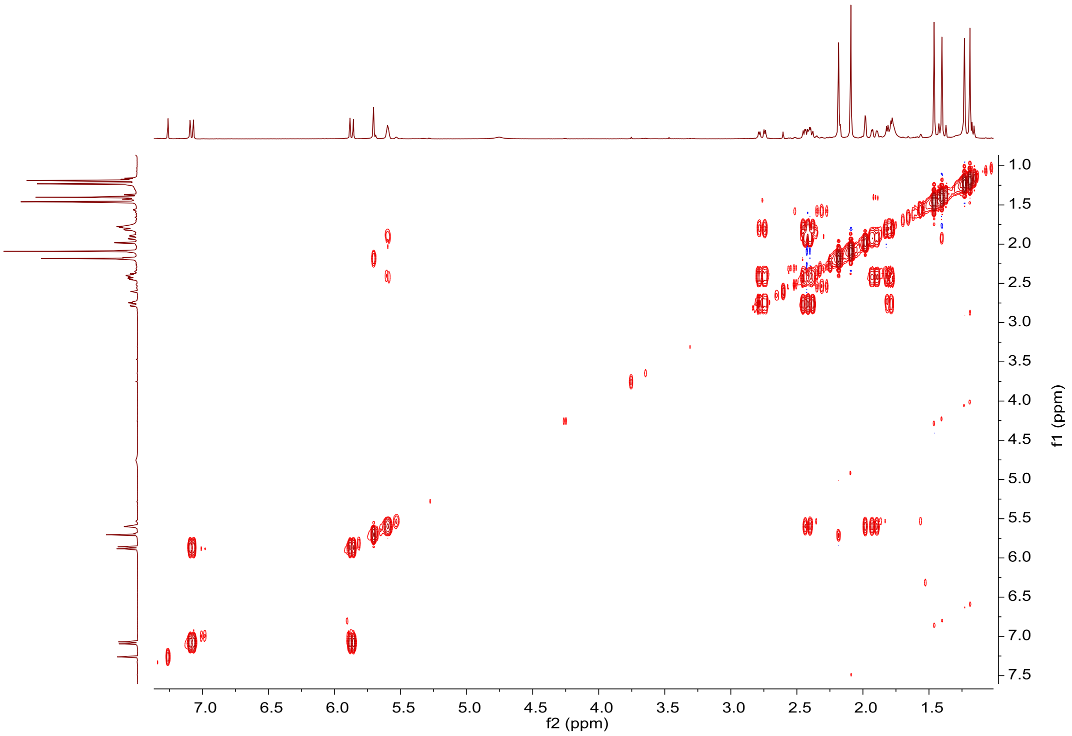

Figure S65. HMBC spectrum of asperversin G (**7**) ($CDCl_3$).

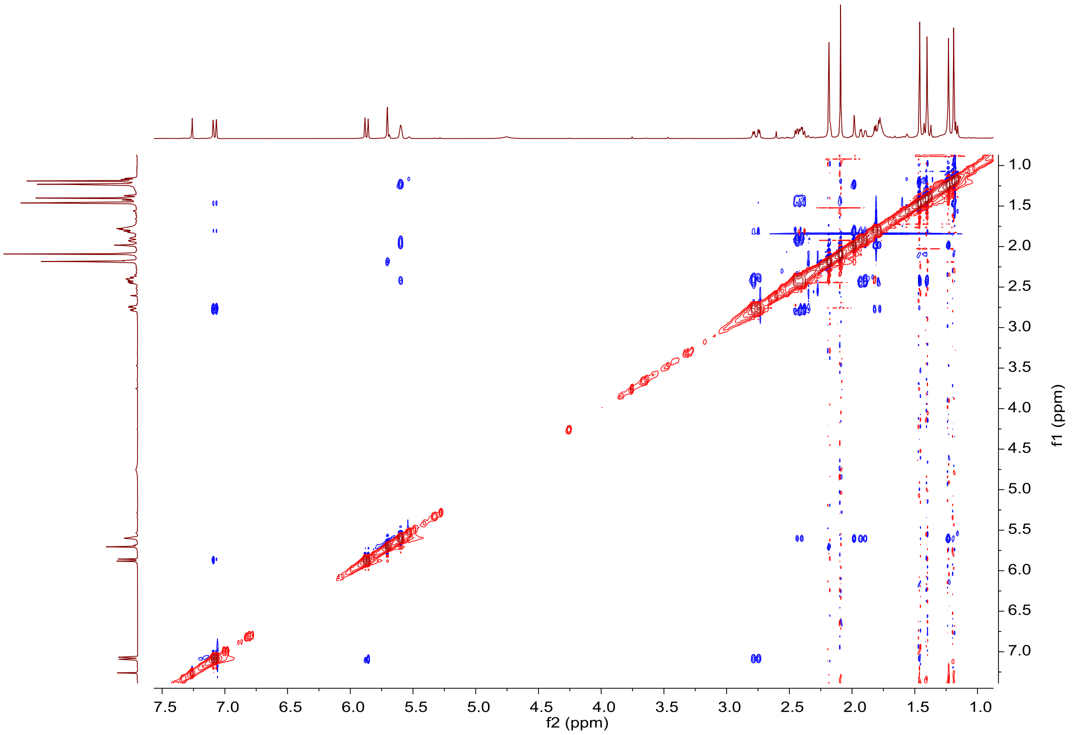

Figure S67. NOESY spectrum of asperversin G (**7**) ($CDCl_3$).

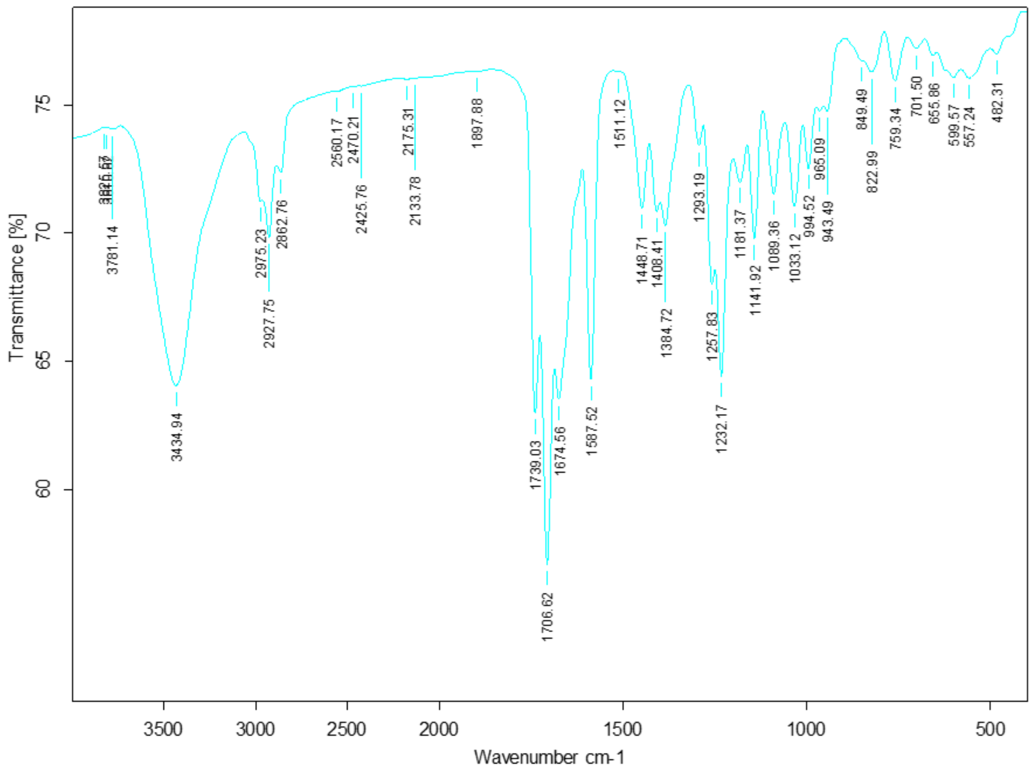

Figure S67. IR spectrum of asperversin G (**7**).

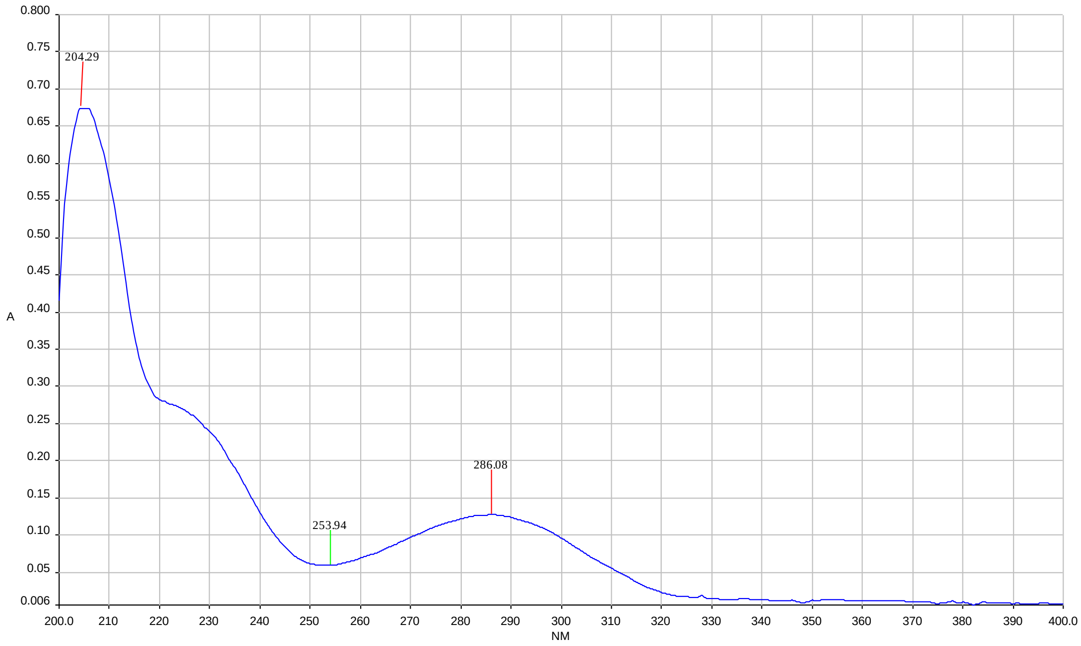

Figure S68. UV spectrum of asperversin G (**7**).
